# Supplementary material for: scConsensus: combining supervised and unsupervised clustering for cell type identification in single-cell RNA sequencing data
Source: BMC Bioinformatics. 2021 Apr 12;22:186. doi: 10.1186/s12859-021-04028-4 (PMC8042883; doi:10.1186/s12859-021-04028-4)
Supplement: Supplementary file 1 — Additional file 1. Supplementary Figures and Tables. [file 12859_2021_4028_MOESM1_ESM.pdf]

Supplement *scConsensus*

Supplementary Figures

Cell type labels based on supervised clustering with RCA (columns); **Manual cell type labels based on unsupervised clustering with Seurat** (rows)

	T cells	B cells	Natural killer cells	Monocytes	Myeloid Progenitors	Lymphoid Progenitors	Natural killer and T cells	DC	Unkonwn
CD4 Naïve T-cell	5440	0	2	1	0	1	33	0	7
CD4 Memory T-cell	4719	0	0	0	0	0	0	0	0
CD8 Naïve T-cell	3988	0	0	0	2	0	16	0	0
B cells	0	2631	0	0	0	0	0	0	0
Natural killer cells	1	0	2548	0	0	0	9	0	4
CD14 Monocytes	0	0	0	2468	0	0	0	85	3
CD34+ cells	0	1	0	40	1234	785	2	2	35
CD8 Cytotoxic T-cells	439	0	156	0	0	0	565	0	0
myeloid DC	0	0	0	0	0	0	0	77	0
plasmacytoid DC	0	0	0	3	0	0	0	56	0
Unkown	0	0	0	0	0	0	0	0	0

Supplementary Figure S1: Consensus matrix of cell type annotation for PBMC data. Columns show cell type labels based on supervised clustering with RCA, rows show cell type labels based on unsupervised clusters using Seurat and a subsequent marker based annotation. The most informative labelling could be obtained by combining both annotations, e.g. the immune cell annotation derived from Seurat clustering and the Progenitor annotation derived by RCA. Figure based on data from Zheng et al. (2017).

A) CBMC

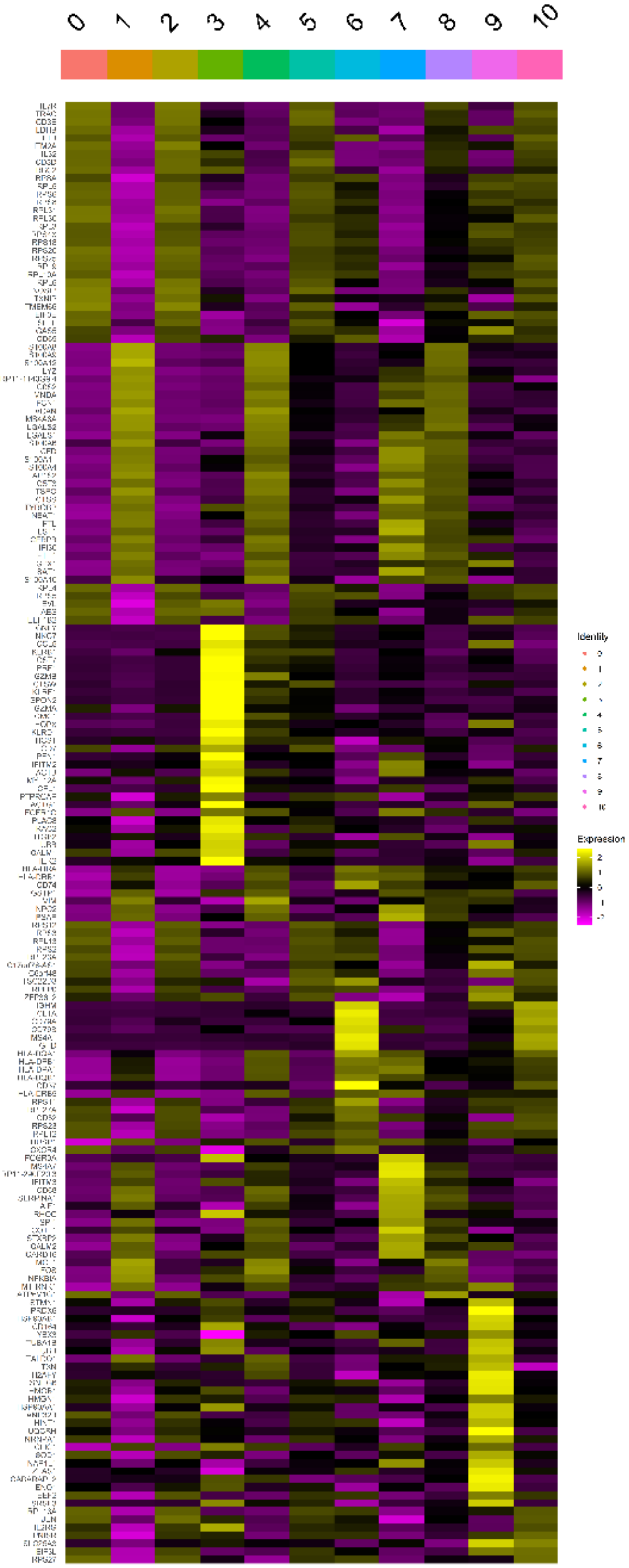

B) PBMC Drop-Seq

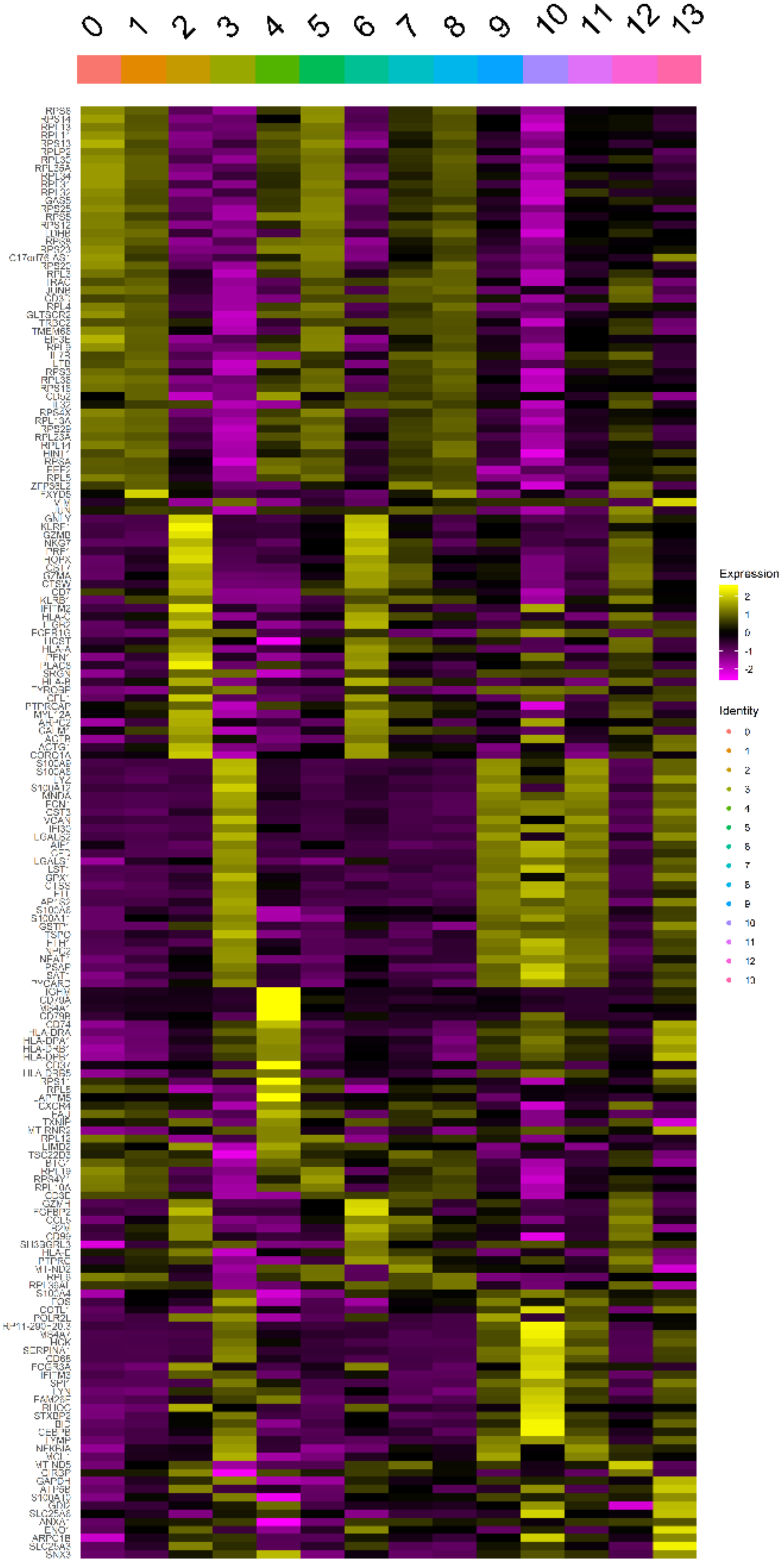

C) MALT

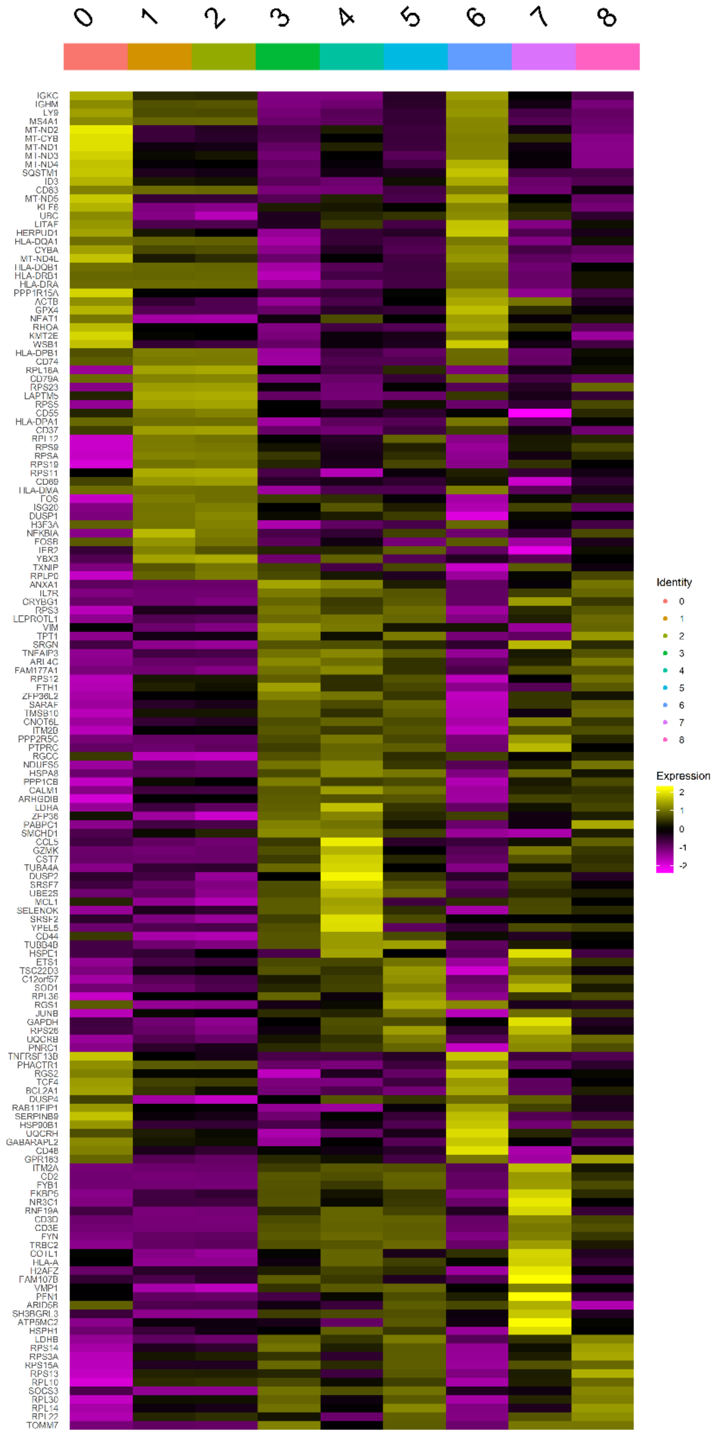

0
1
2
3
4
5
6
7
8
IGKC
IGHM
LY9
MS4A1
MT-ND2
MT-CYB
MT-ND1
MT-ND3
MT-ND4
SQSTM1
ID3
CD83
MT-ND5
KLF6
UBC
LITAF
HERPUD1
HLA-DQA1
CYBA
MT-ND4L
HLA-DQB1
HLA-DRB1
HLA-DRA
PPP1R15A
ACTB
GPX4
NFAT1
RHOA
KMT2E
WSB1
HLA-DPB1
CD74
RPL18A
CD79A
RPS23
LAPTM5
RPS5
CD55
HLA-DPA1
CD37
RPL12
RPS8
RPSA
RPS19
RPS11
CD69
HLA-DMA
FOS
ISG20
DUSP1
H3F3A
NFKBIA
FOSB
IER2
YBX3
TXNIP
RPLP0
ANXA1
IL7R
CRYBG1
RPS3
LEPROTL1
VIM
TPT1
SRGN
TNFAIP3
ARL4C
FAM177A1
RPS12
FTH1
ZFP36L2
SARAF
TMSB10
CNOT6L
ITM2B
PPP2R5C
PTPRC
RGCC
NDUFS5
HSPA8
PPP1CB
CALM1
ARHGDIB
LDHA
ZFP36
PABPC1
SMCHD1
CCL5
GZMK
CST7
TUBA4A
DUSP2
SRSF7
UBE2S
MCL1
SELENOK
SRSF2
YPEL5
CD44
TUBB4B
HSPE1
ETS1
TSC22D3
C12orf57
SOD1
RPL36
RGS1
JUNB
GAPDH
RPS26
UQCRB
PNRC1
TNFRSF13B
PHACTR1
RGS2
TCF4
BCL2A1
DUSP4
RAB11FIP1
SERPINB9
HSP90B1
UQCRH
GABARAPL2
CD48
GPR183
ITM2A
CD2
FYB1
FKBP5
NR3C1
RNF19A
CD3D
CD3E
FYN
TRBC2
COTL1
HLA-A
H2AFZ
FAM107B
VMP1
PFN1
ARID5B
SH3BGRL3
ATP5MC2
HSPH1
LDHB
RPS14
RPS3A
RPS15A
RPS13
RPL10
SOCS3
RPL30
RPL14
RPL22
TOMM7
Identity
0
1
2
3
4
5
6
7
8
Expression
2
1
0
-1
-2

D) PBMC

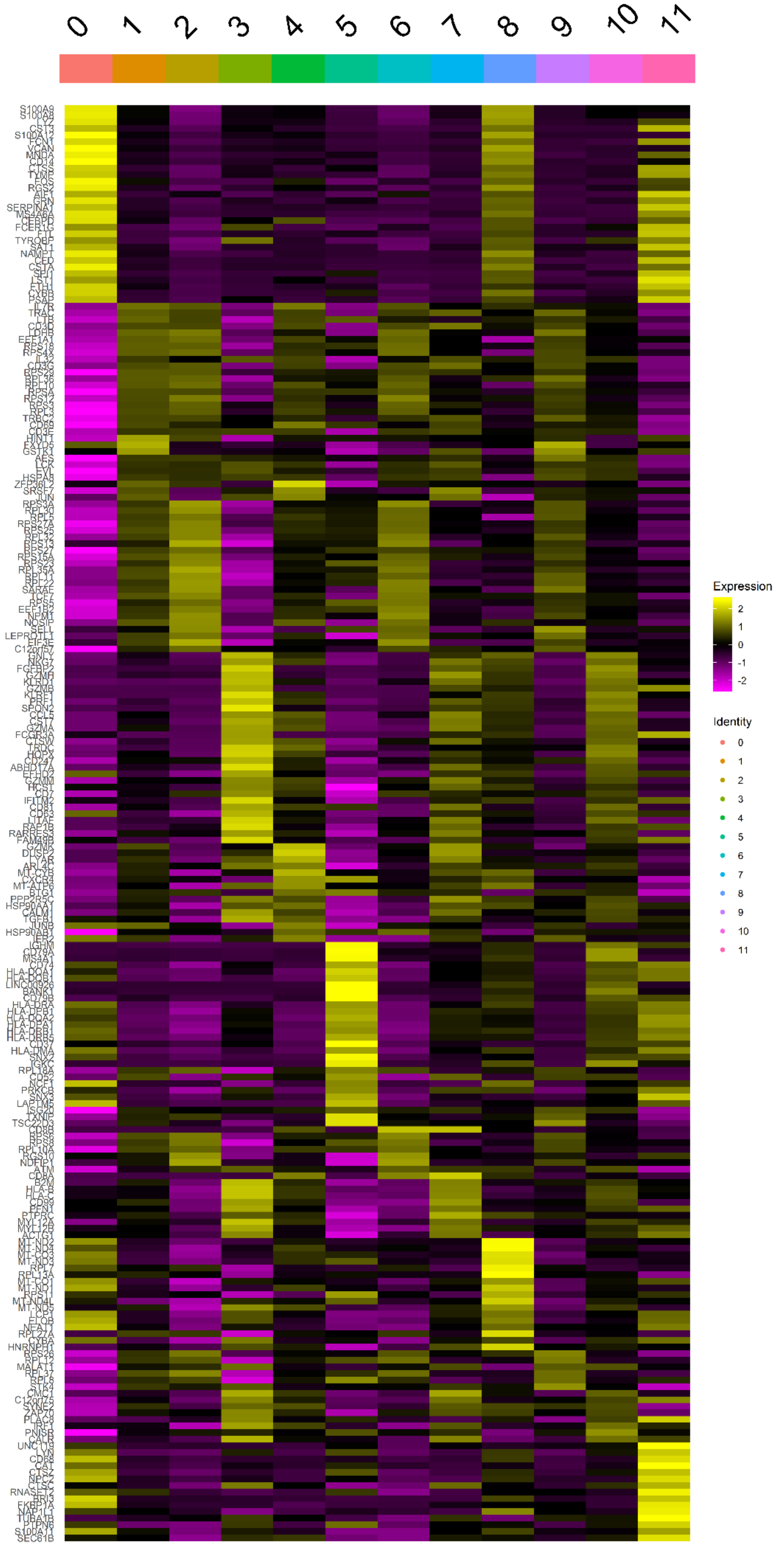
0
1
2
3
4
5
6
7
8
9
10
11
Expression
2
1
0
-1
-2
Identity
0
1
2
3
4
5
6
7
8
9
10
11

E) PBMC-VDJ

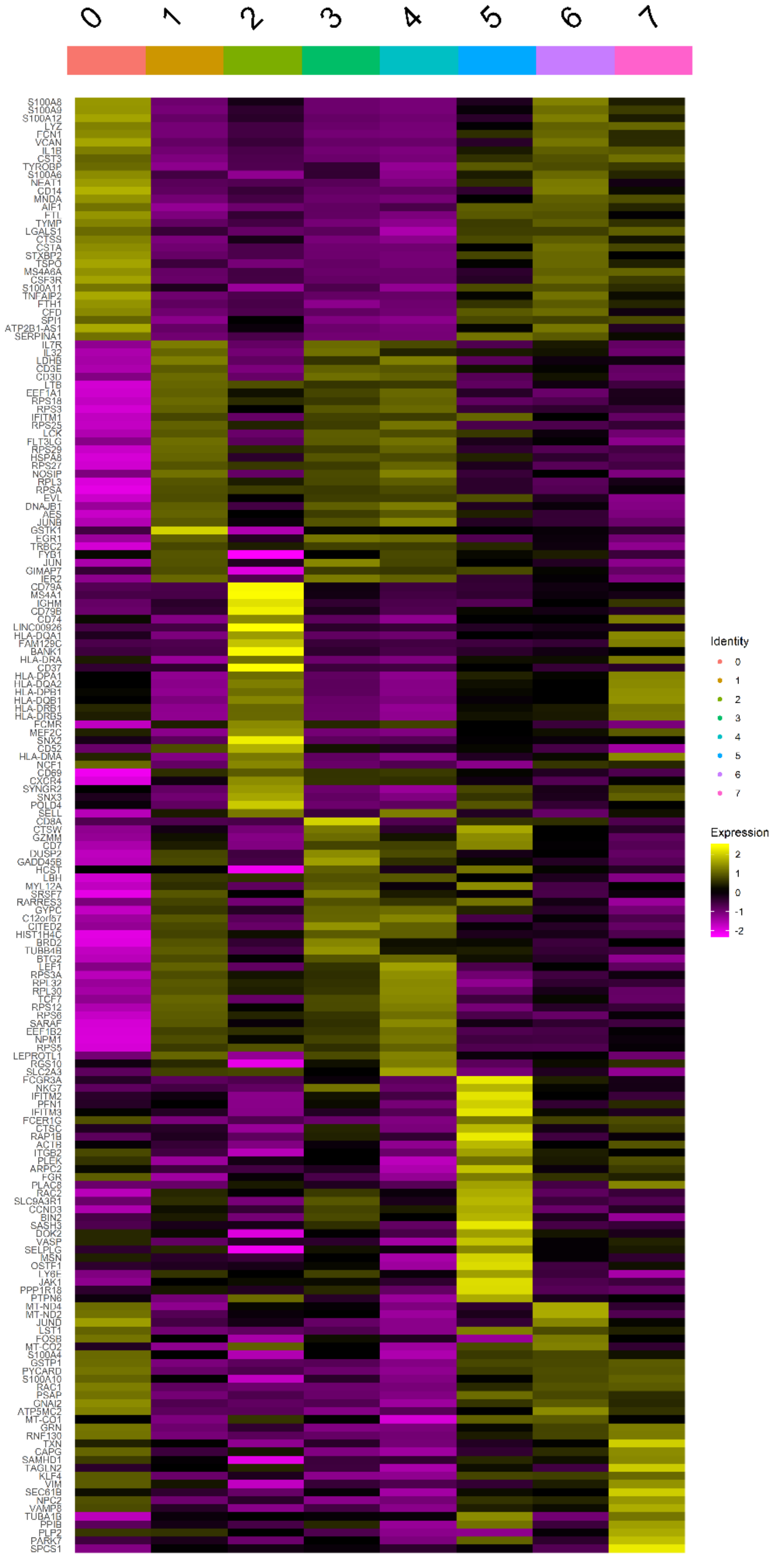

Supplementary Figure S2: Heat maps showing cluster-specific DE gene expression for A) CBMC B) PBMC Drop-Seq C) MALT D) PBMC E) PBMC-VDJ

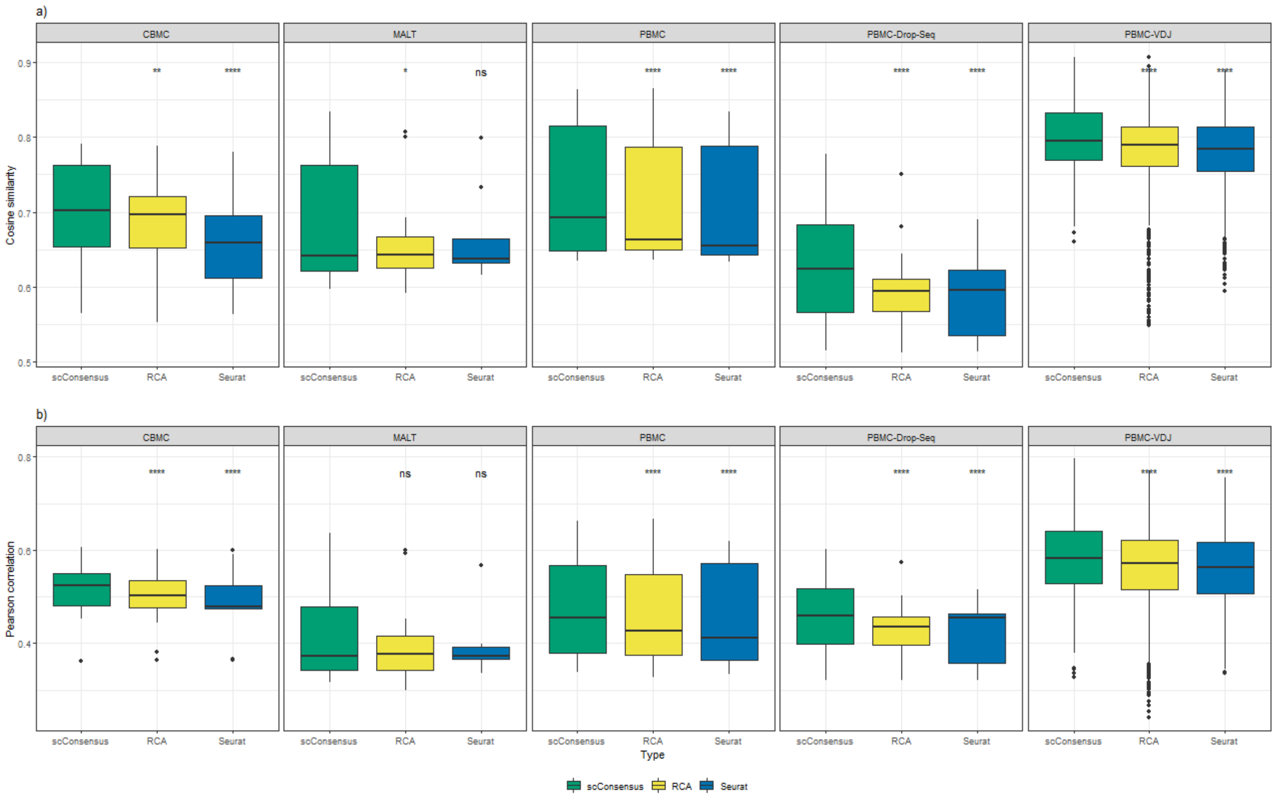

Supplementary Figure S3: Cosine and Pearson correlation for bootstrapping on the gene-expression space using a variance threshold of 0.5

A) Ground Truth

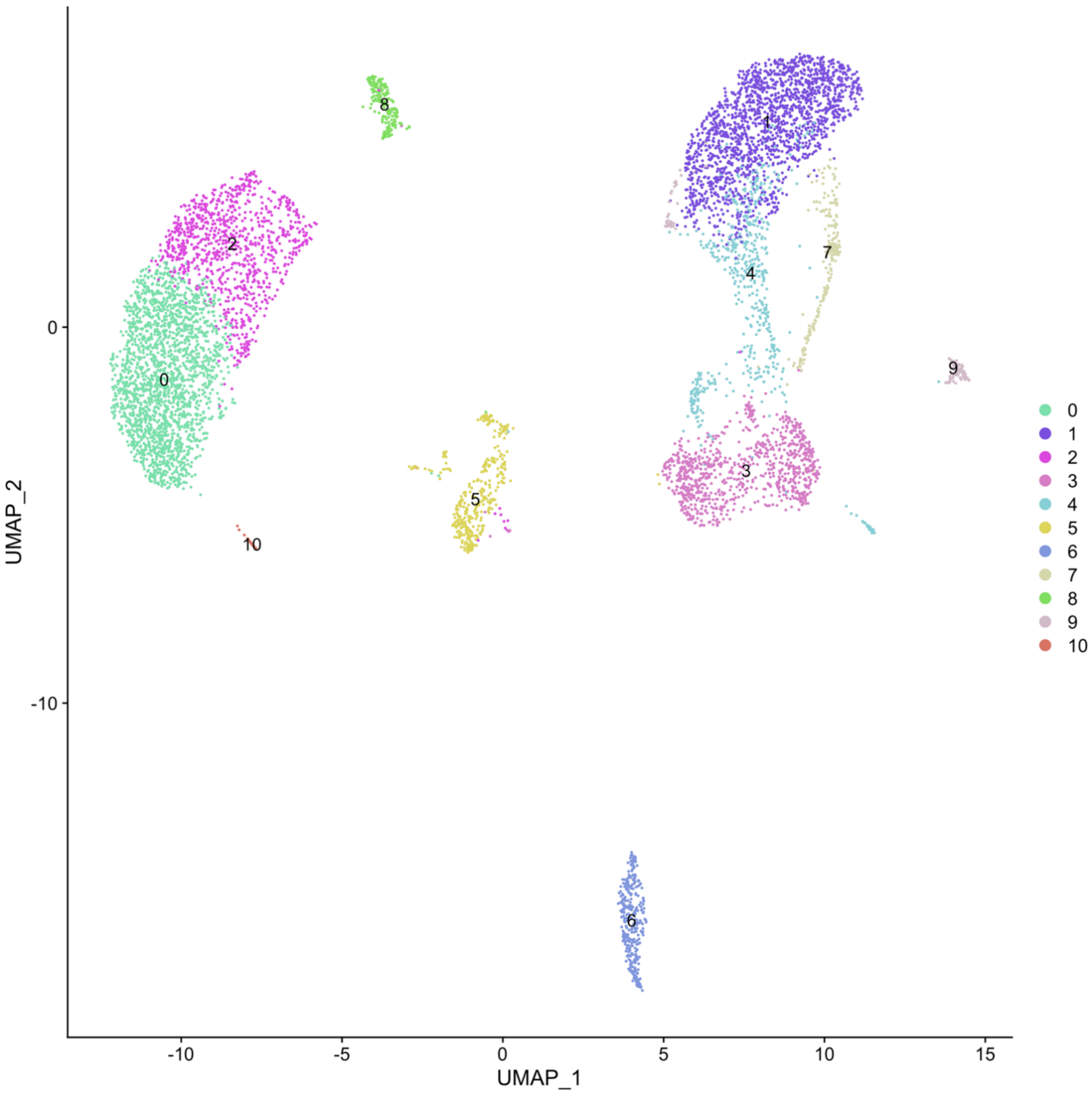

B) scConsensus

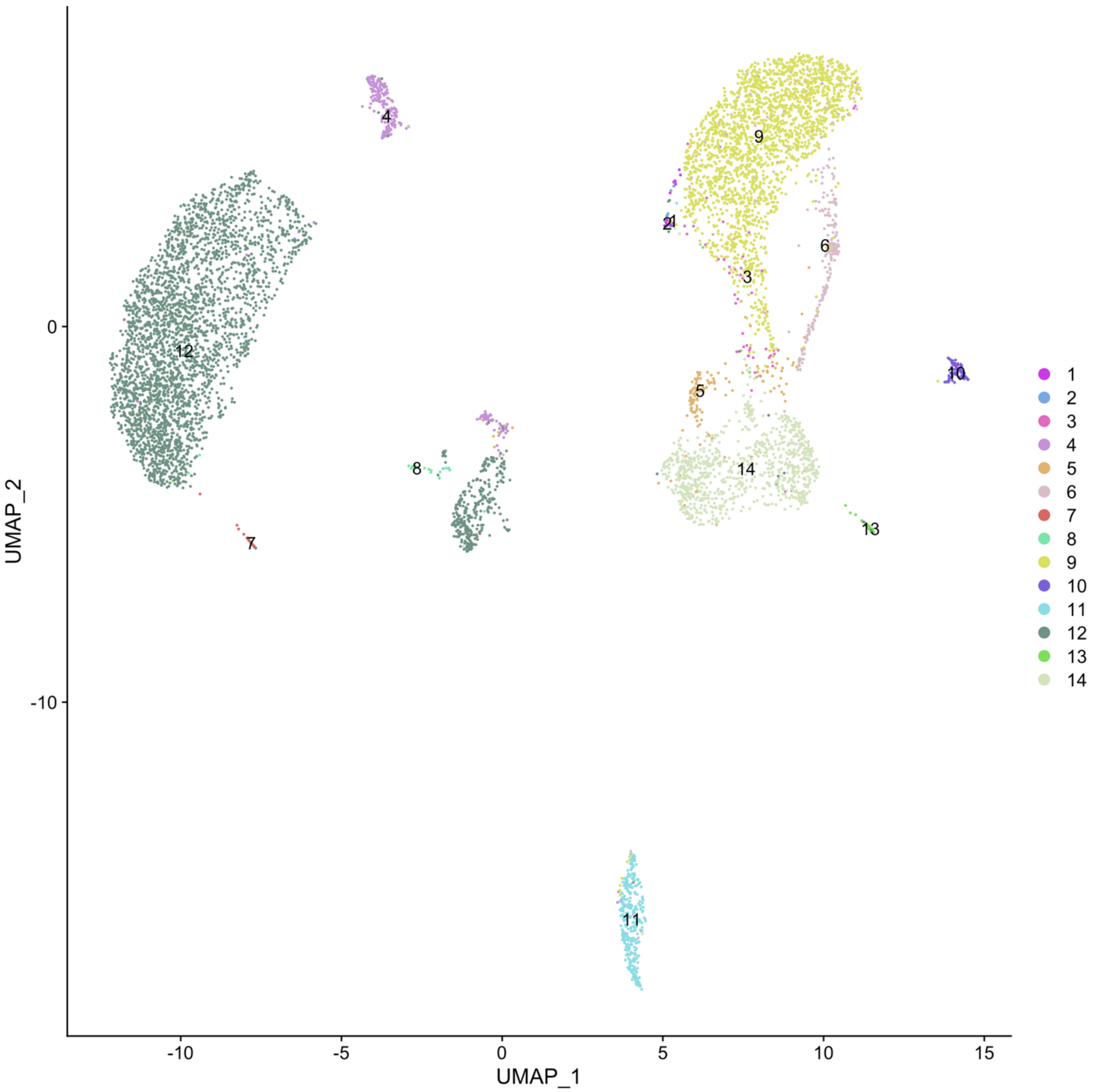

C) Seurat

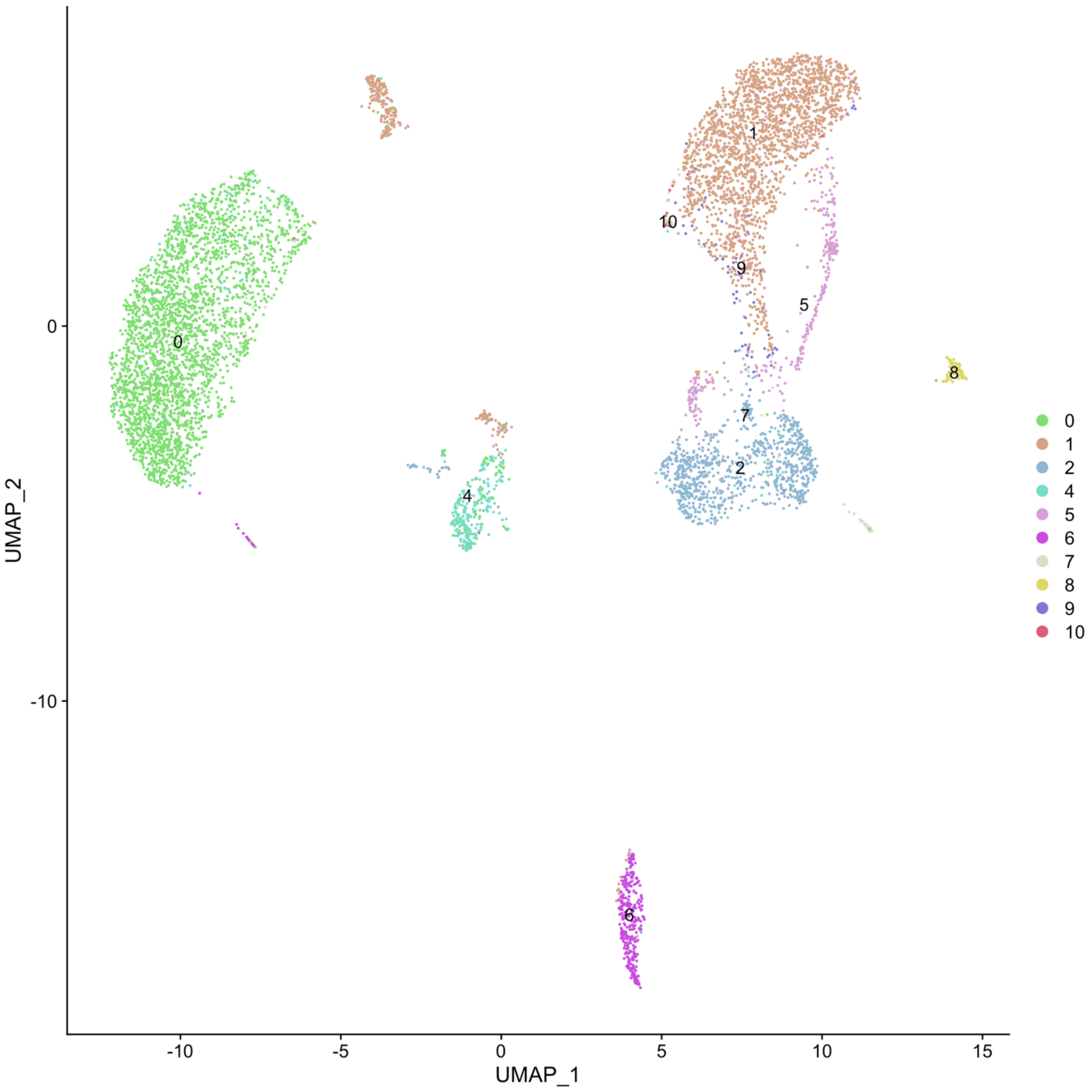

D) RCA

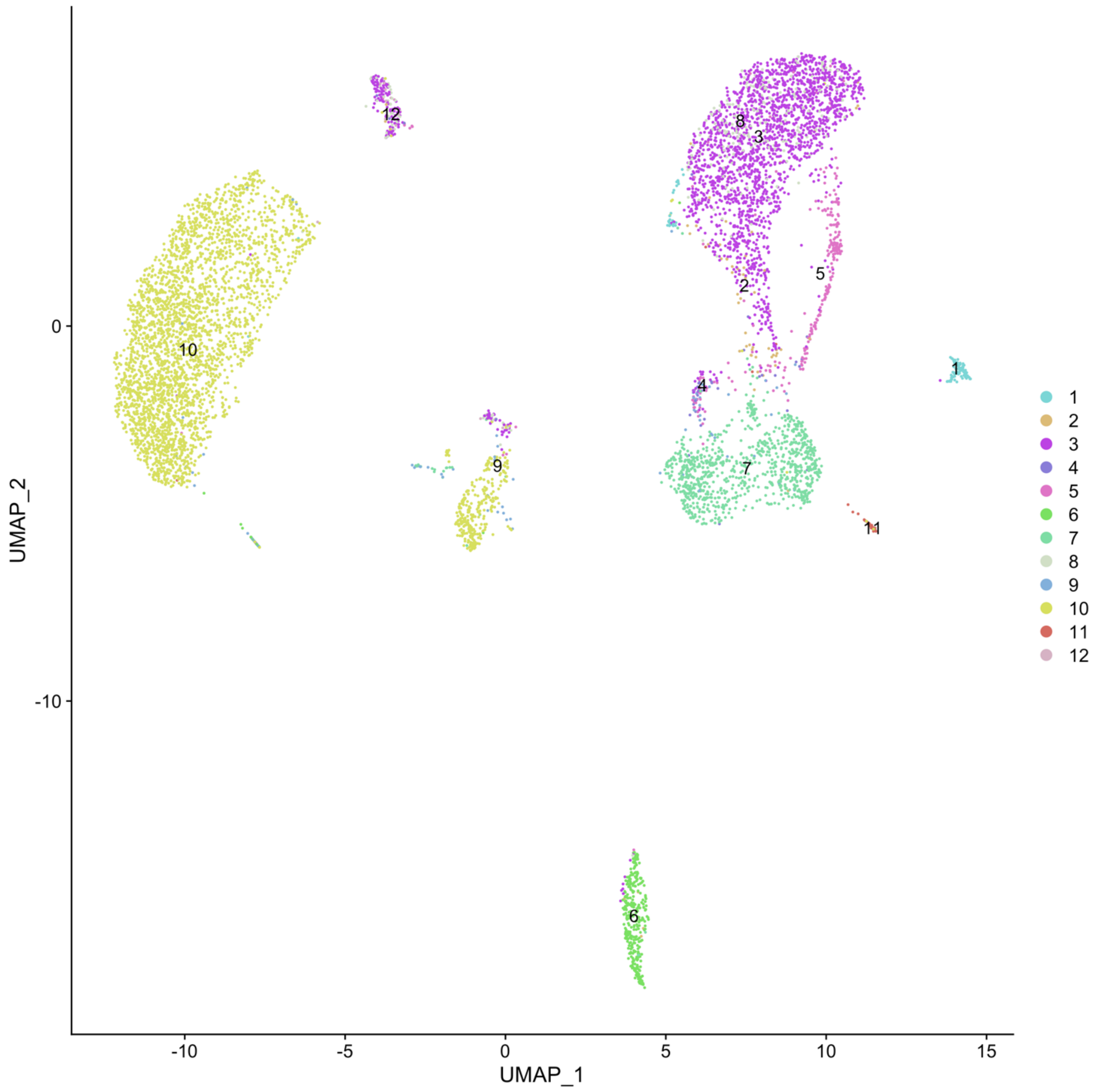

Supplementary Figure S5: UMAP visualizations of cells in ADT space colored by A) Antibody-cluster ground truth B) scConsensus C) Seurat and D) RCA in the antibody expression space for the CBMC Dataset

A) Ground Truth

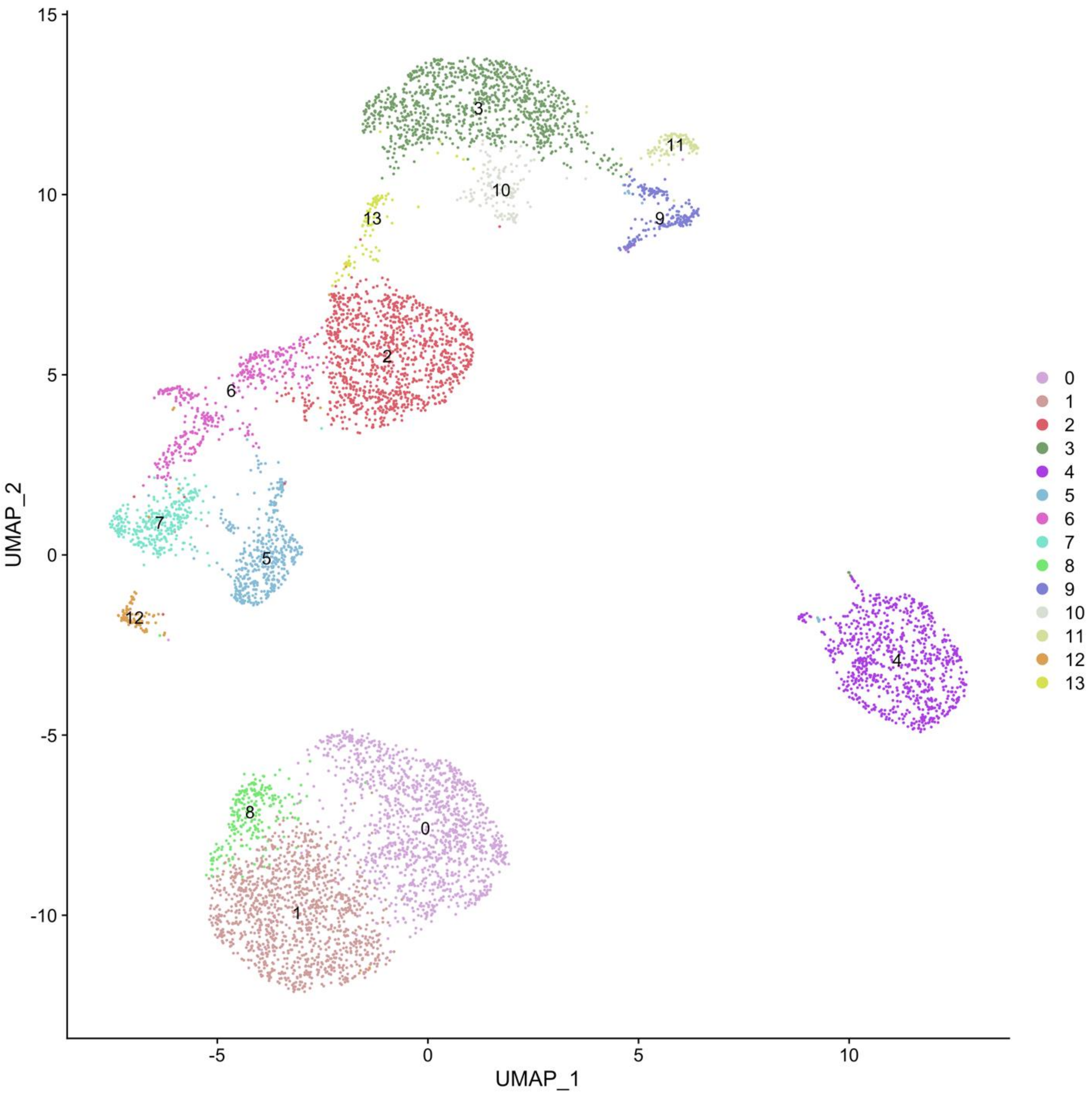

B) scConsensus

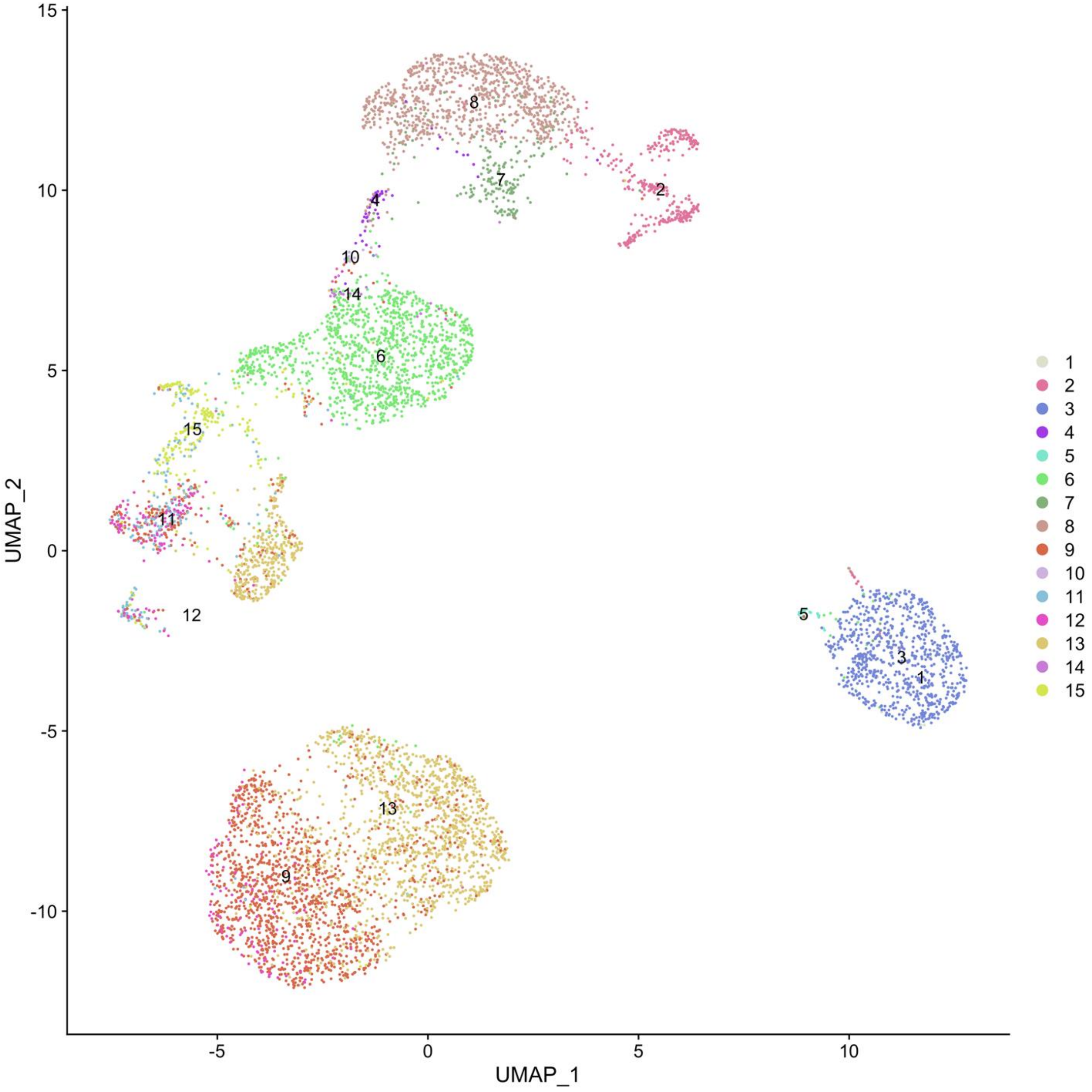

C) Seurat

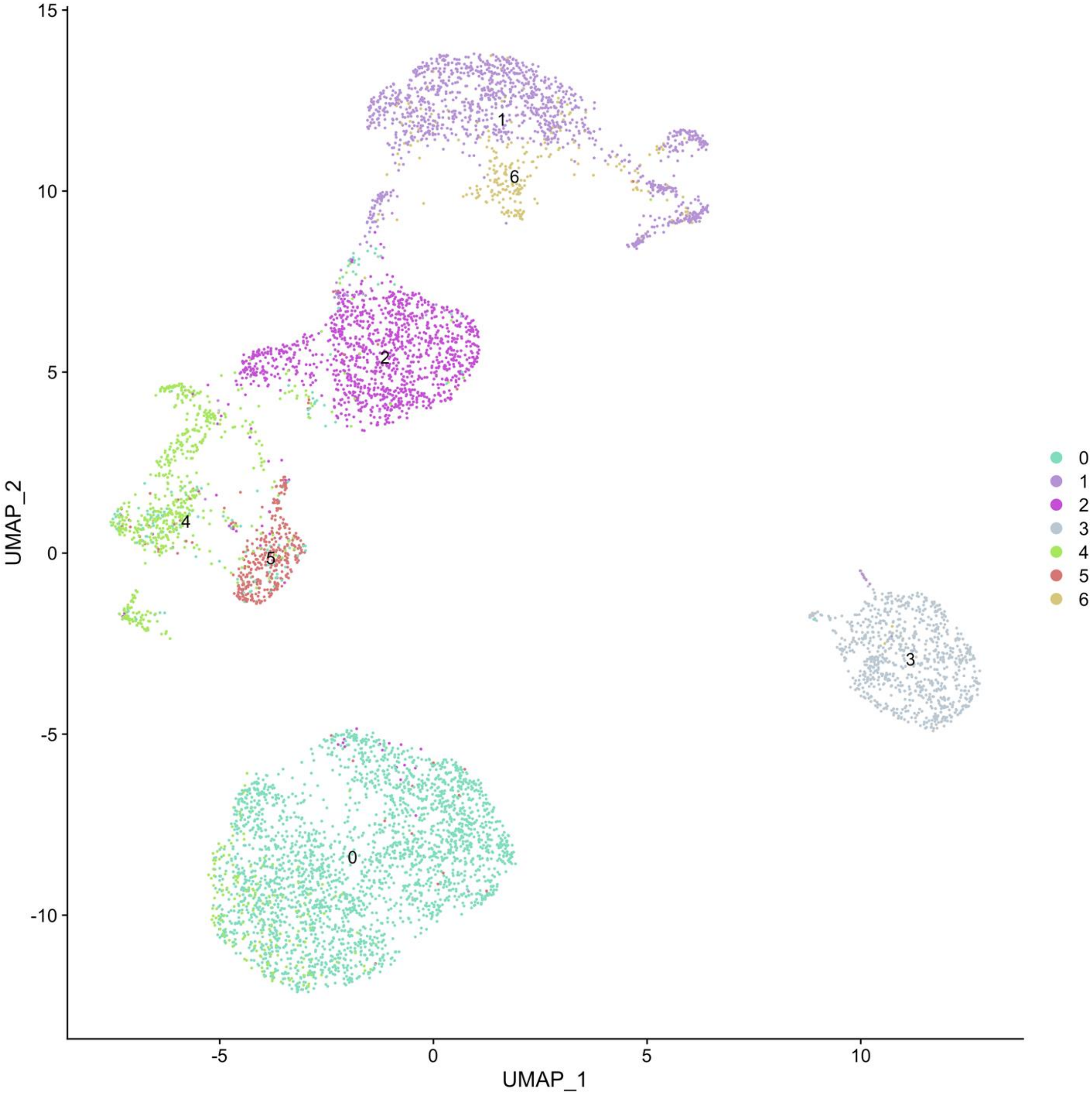

D) RCA

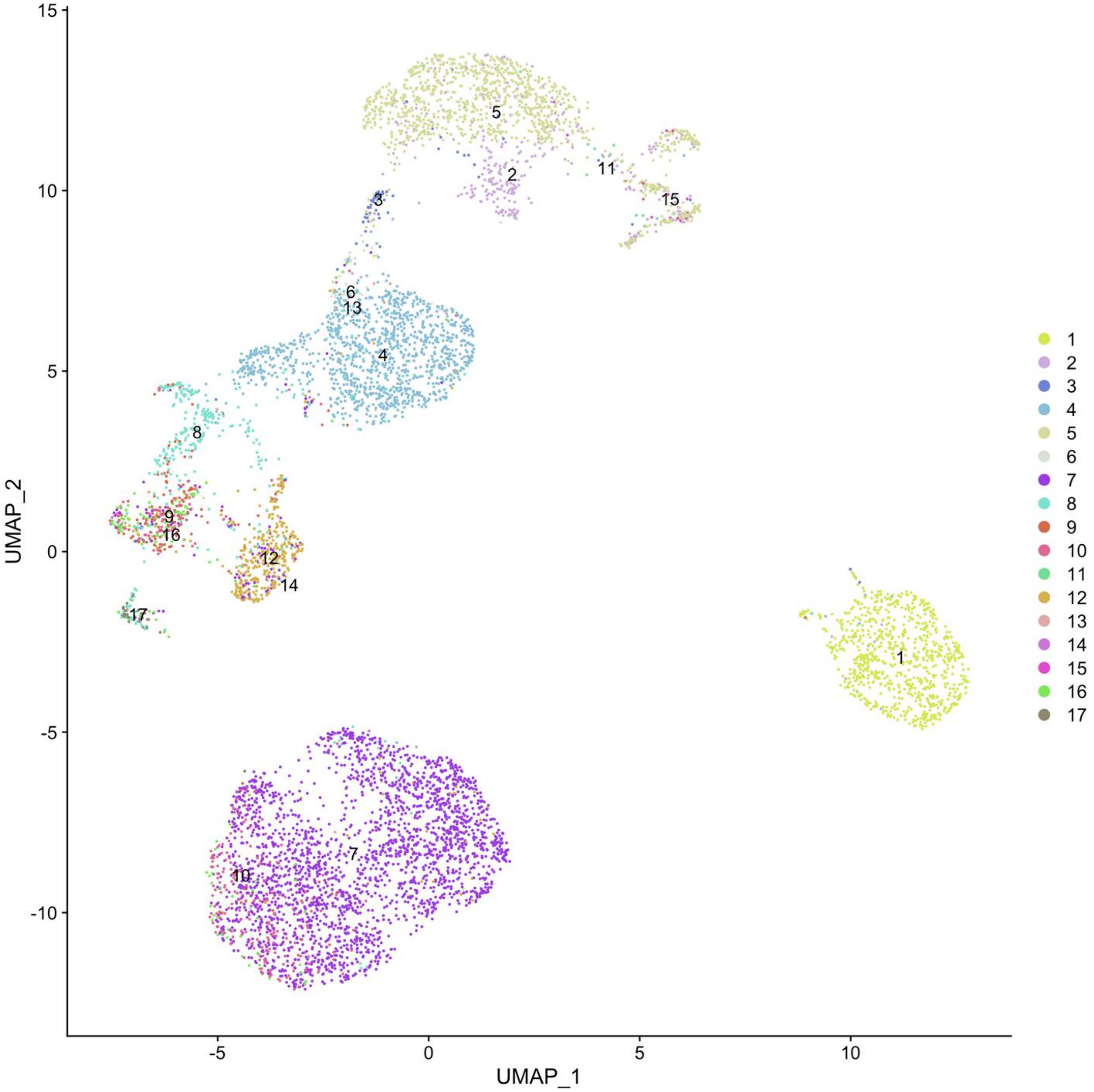

Supplementary Figure S6: UMAP visualizations of cells in ADT space colored by A) Antibody-cluster ground truth B) scConsensus C) Seurat and D) RCA in the antibody expression space for the PBMC Drop-Seq dataset

A) Ground Truth

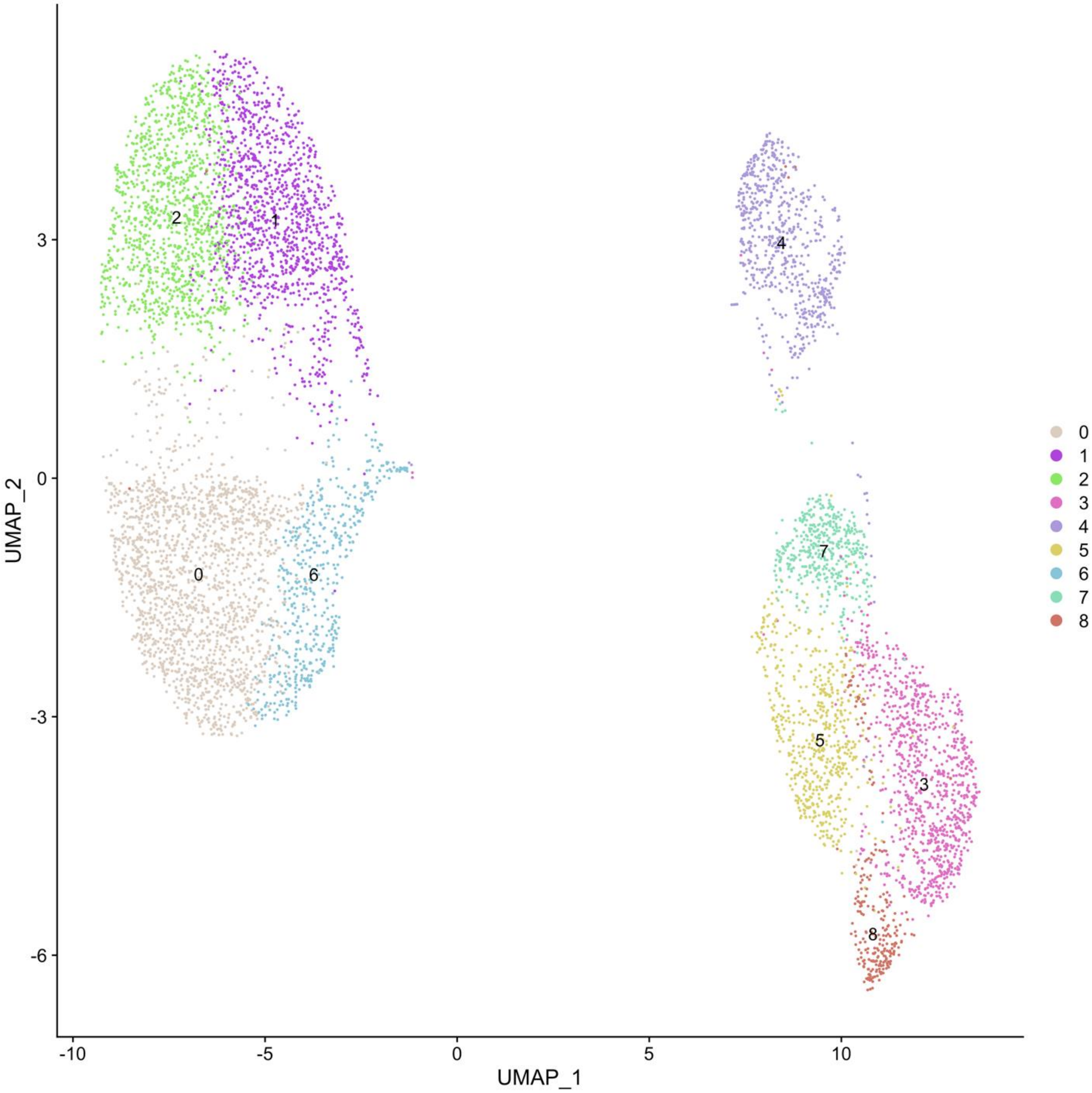

B) scConsensus

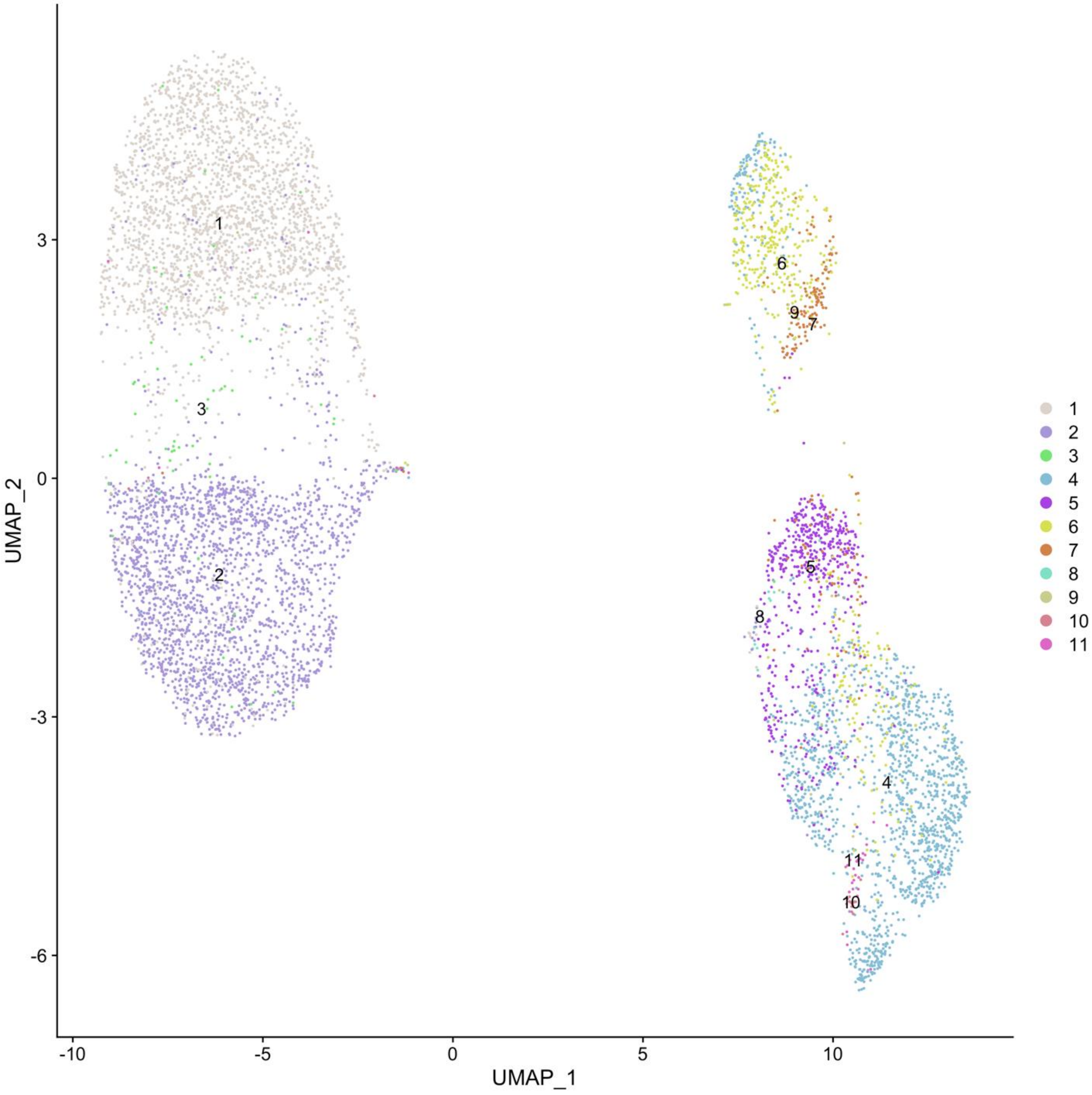

C) Seurat

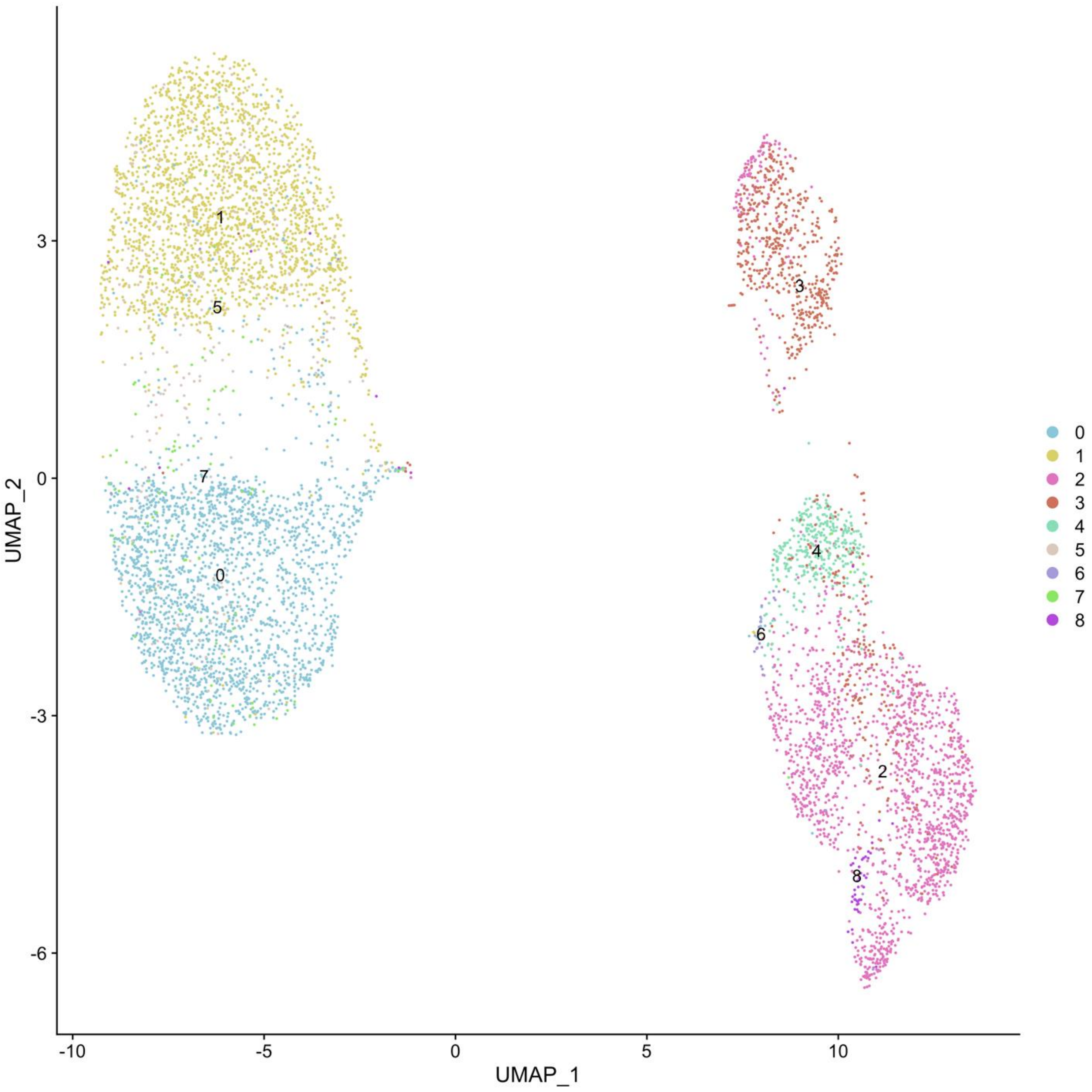

D) RCA

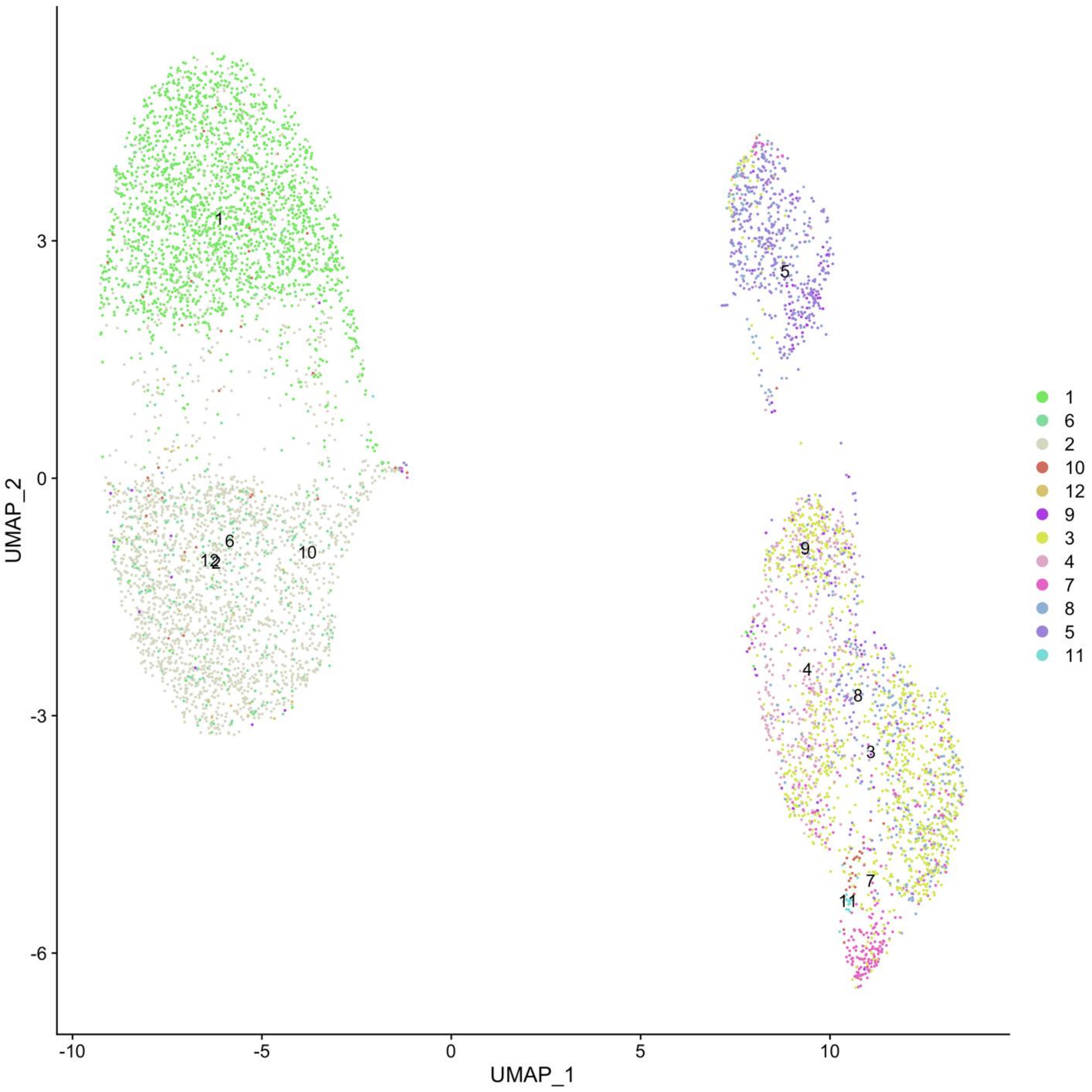

Supplementary Figure S7: UMAP visualizations of cells in ADT space colored by A) Antibody-cluster ground truth B) scConsensus C) Seurat and D) RCA in the antibody expression space for the MALT dataset

A) Ground Truth

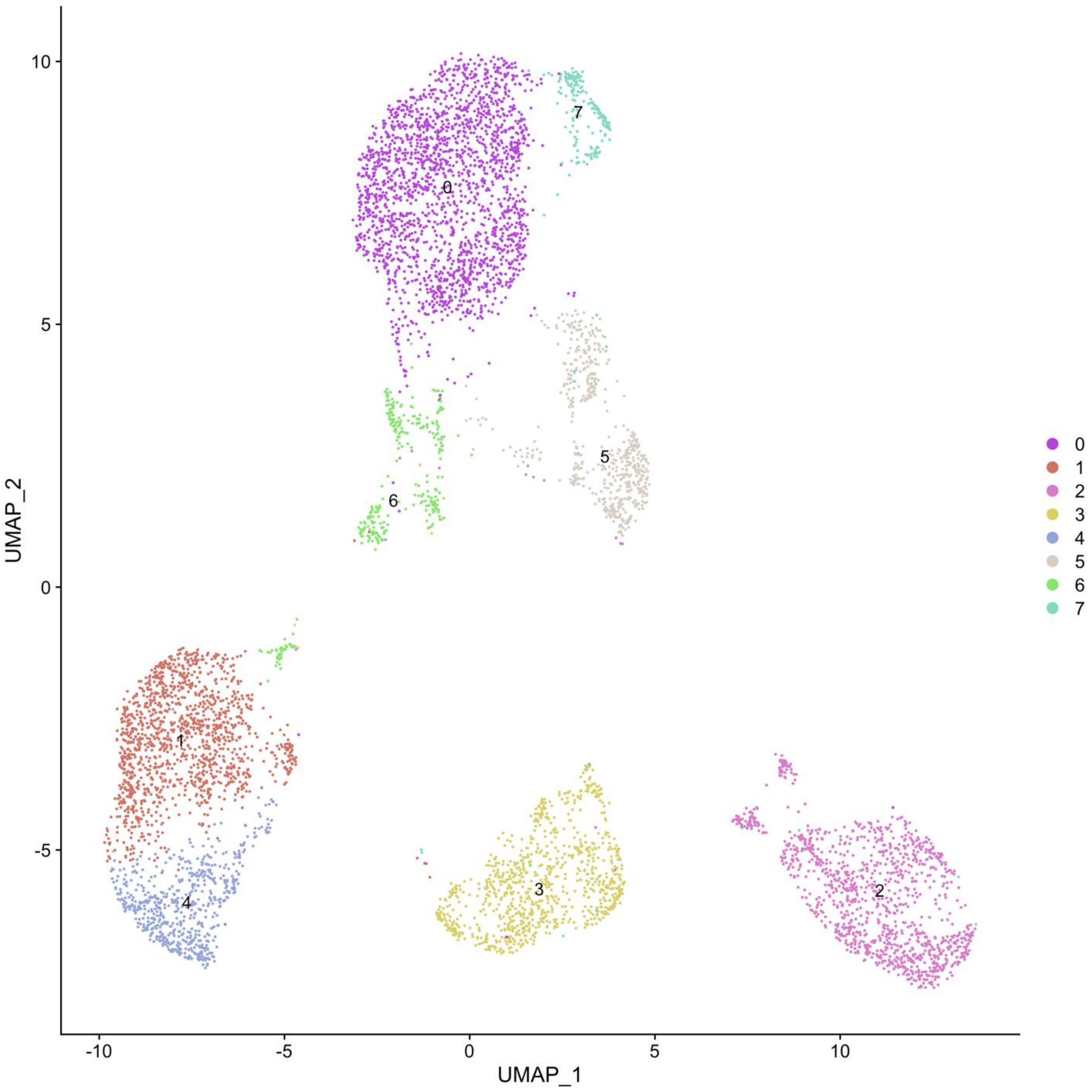

B) scConsensus

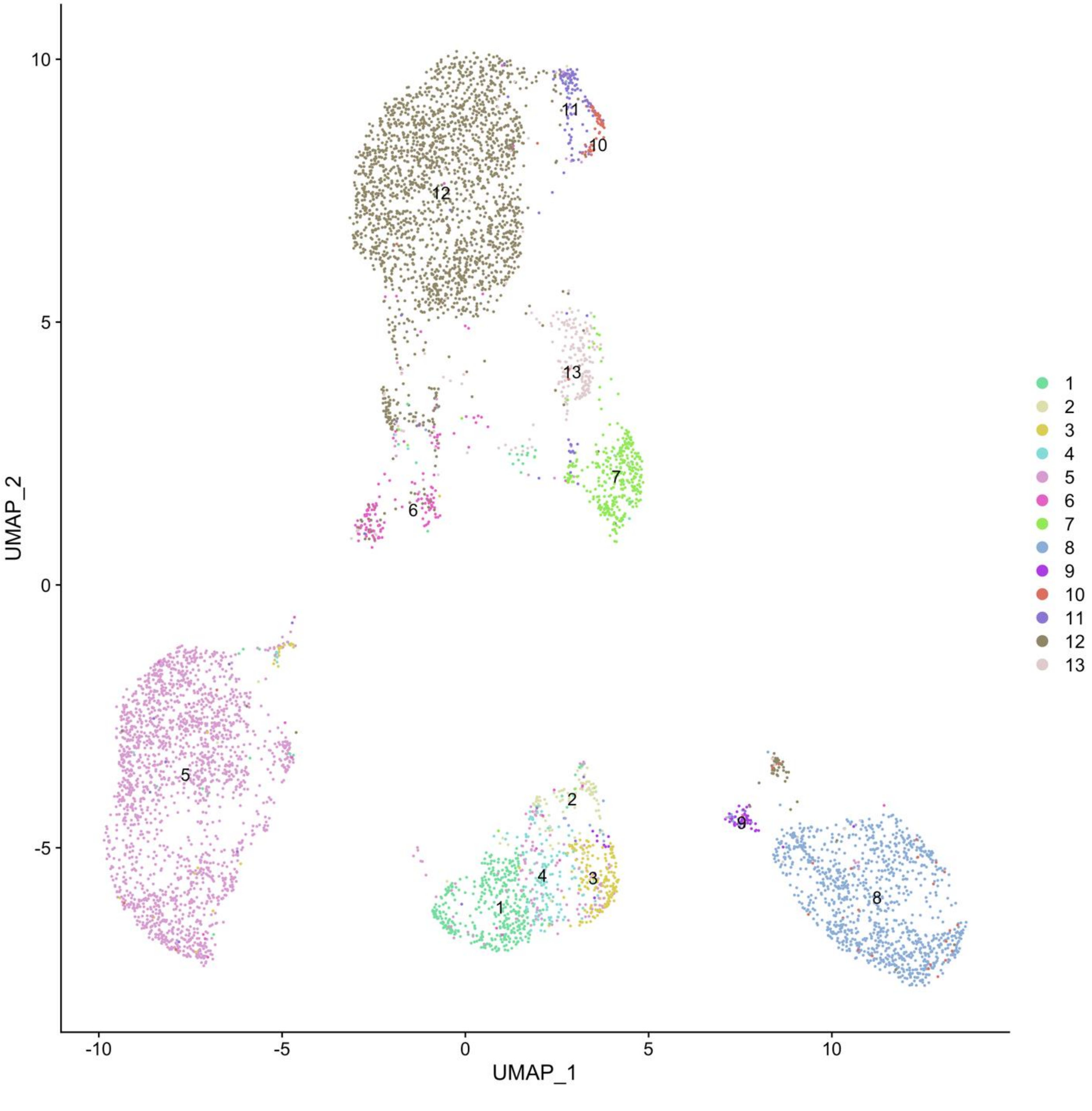

C) Seurat

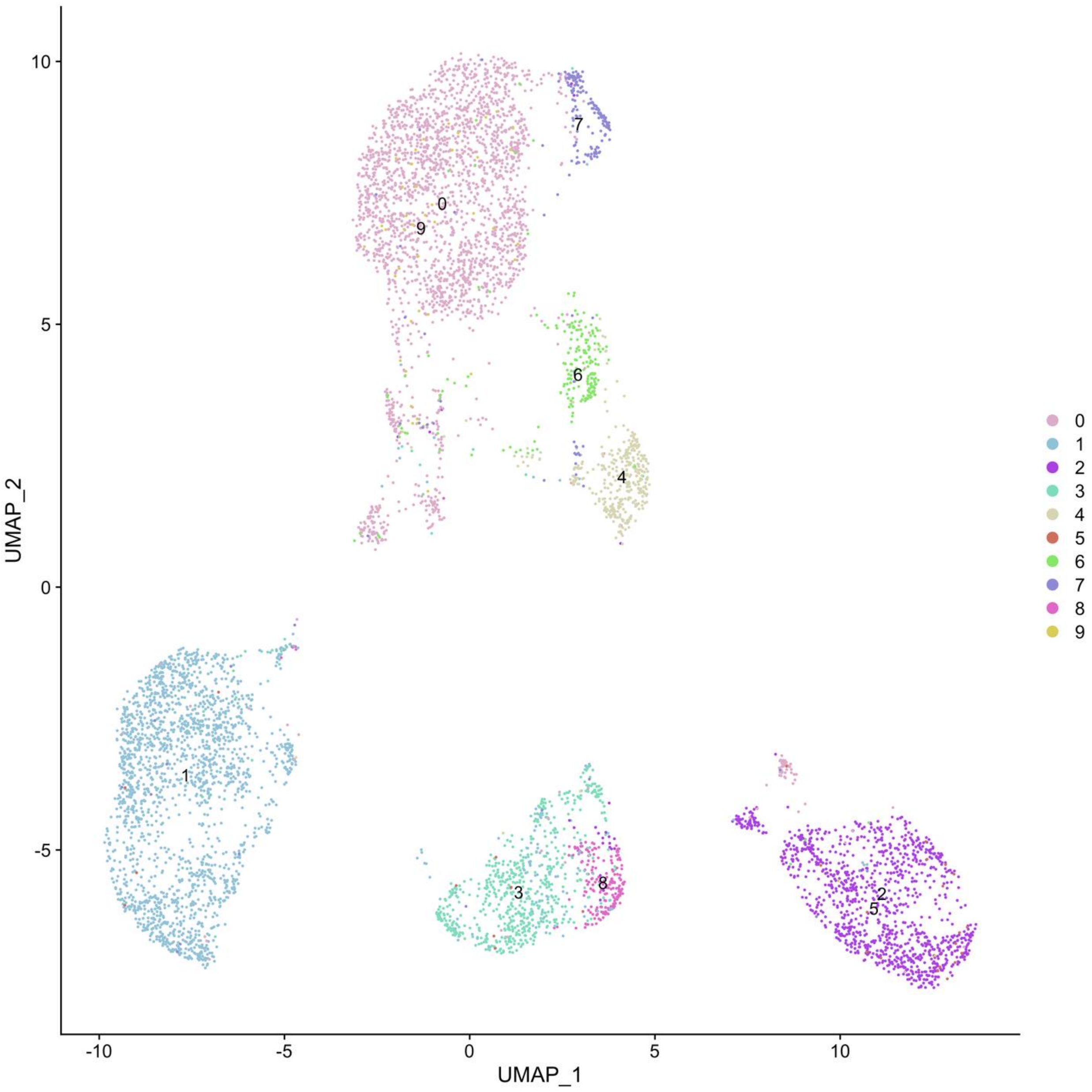

D) RCA

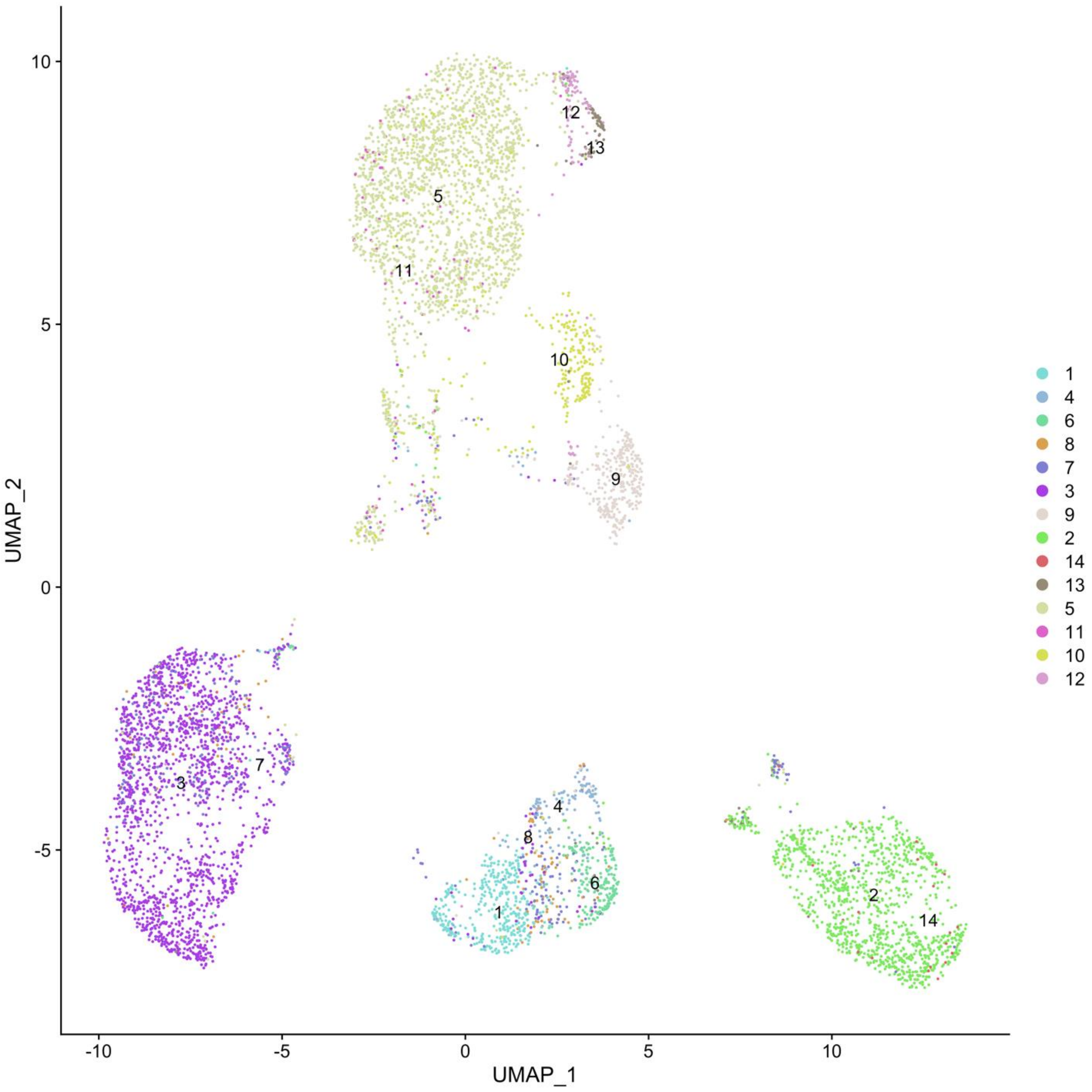

Supplementary Figure S8: UMAP visualizations of cells in ADT space colored by A) Antibody-cluster ground truth B) scConsensus C) Seurat and D) RCA in the antibody expression space for the PBMC-VDJ dataset

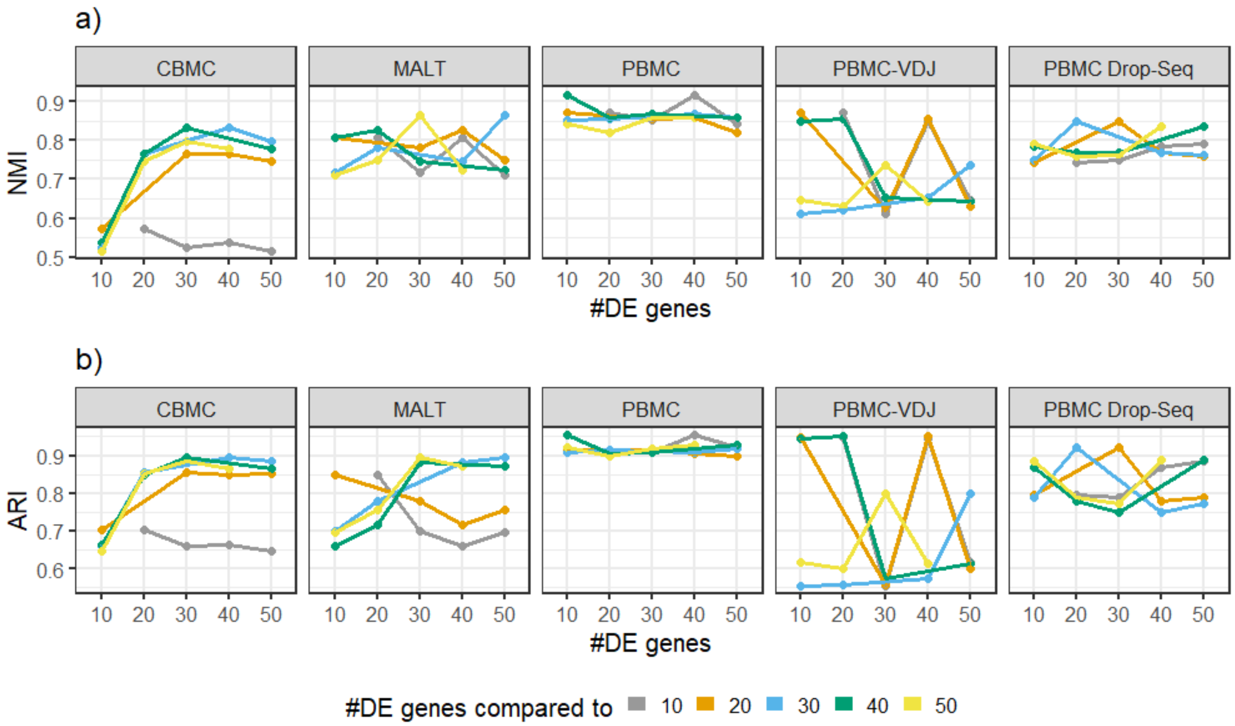

Supplementary Figure S9: Using (a) Normalized Mutual Information (NMI) and (b) Adjusted Rand Index (ARI), we measure the agreement between clustering results obtained using either 10, 20, 30, 40 or 50 DE (x-axis) genes compared to each other (color). Note that self-comparisons are omitted for clarity. For the *CBMC* data set, both scores remain constant from 30 DE genes onwards. For the *MALT* data set, we observe that considering 30 DE genes has a good agreement with a clustering based on 50 and 40 DE genes. Considering the *PBMC* dataset, the number of DE genes does not strongly influence the result at all. Interestingly, there is a lot of variation in the agreement between different clustering results in the *PBMC-VDJ* data. Roughly, clusterings using 10,20 and 40 DE genes agree with each other, while cluster results using 30 and 50 DE genes also agree well. For the PBMC Drop-Seq dataset, the variation is less pronounced, generally we observe that clusters with 10, 40 and 50 DE genes agree well with each other, although the highest scores are obtaine using 20 or 30 DE genes.

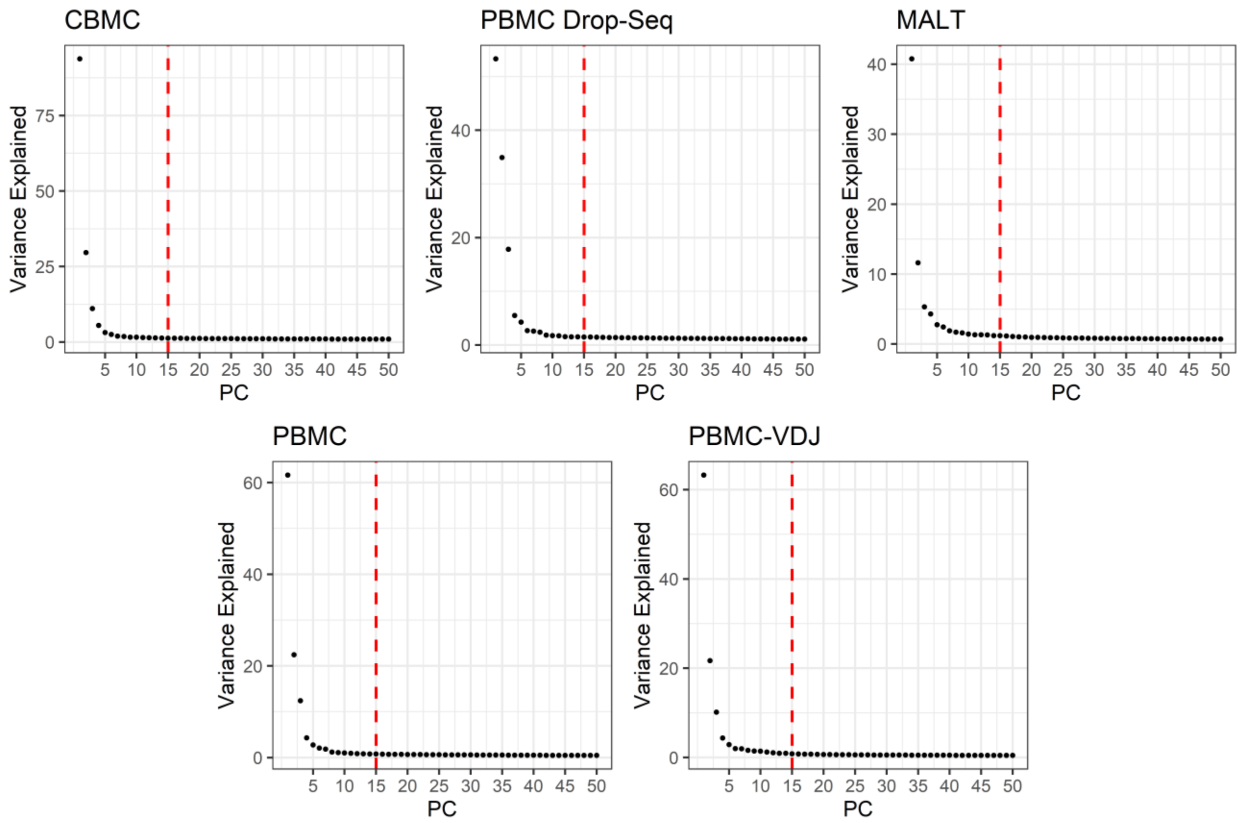

Supplementary Figure S10: Elbow plots showing the variance explained by principal components (PCs) for all datasets. Here, the red dashed line indicates the number of PCs that were selected for each dataset (15 PCs in each case). This figure shows that, for all datasets, the curve flattens out before 15 PCs, and there is little change in the variance explained by the subsequent PCs. Thus, we select 15 as a reasonable and consistent number of PCs for analysis of these datasets.

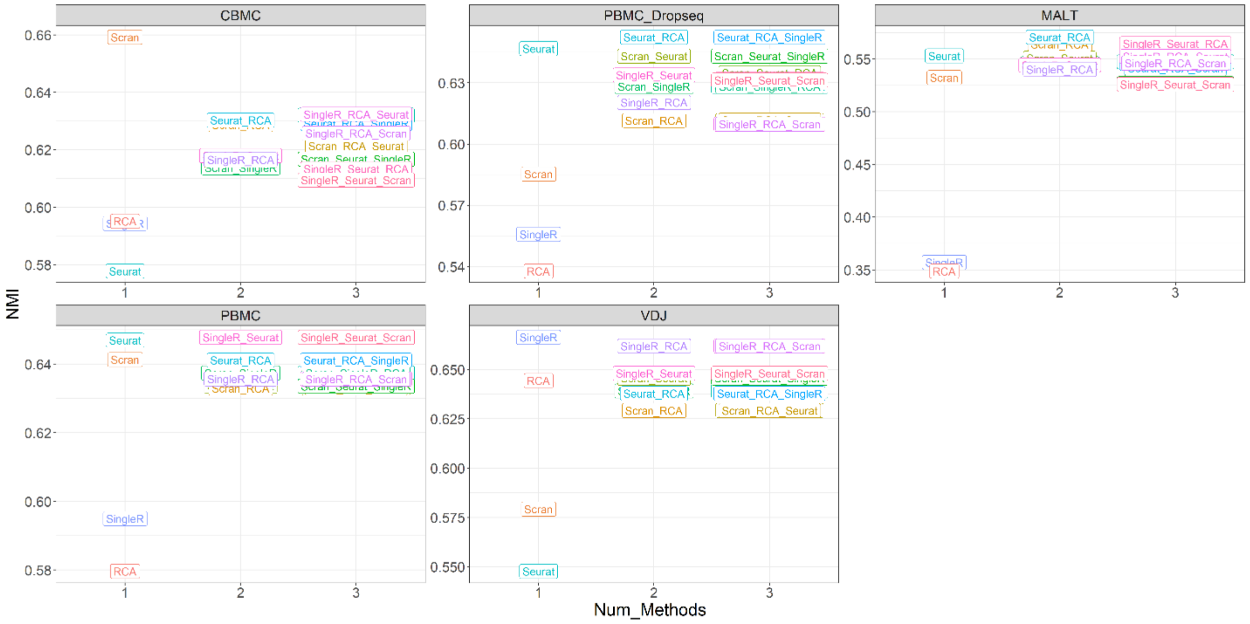

Supplementary Figure S11: Normalized Mutual Information (NMI) of clustering results with antibody-derived ground truth for combinations of two or more clustering methods. X-axis represents the number of methods combined.

Supplementary Tables

Dataset	Data Type	# genes	# cells	Reference
Cord Blood 10X (CBMC)	10X CITE-Seq UMI + ADT (Cord Blood Mononuclear Cells)	4341	7817	*Stoeckius, Marlon, et al. "Simultaneous epitope and transcriptome measurement in single cells." Nature methods 14.9 (2017): 865.*
Peripheral Blood Drop-Seq (PBMC Drop-Seq)	10X CITE-Seq UMI + ADT	3976	7583	*Stoeckius, Marlon, et al. "Simultaneous epitope and transcriptome measurement in single cells." Nature methods 14.9 (2017): 865.*
Mucosa-Associated Lymphoid Tissue 10X (MALT)	10X CITE-Seq UMI + ADT	4713	8242	*https://support.10xgenomics.com/single-cell-gene-expression/datasets/3.0.0/malt_10k_protein_v3*
Peripheral Blood 10X (PBMC)	10X CITE-Seq UMI + ADT	5924	7750	*https://support.10xgenomics.com/single-cell-gene-expression/datasets/3.0.0/pbmc_10k_protein_v3*
Peripheral Blood 10X-VDJ (PBMC-VDJ)	10X CITE-Seq UMI + ADT (5' Gene Expression)	5185	7627	*https://support.10xgenomics.com/single-cell-vdj/datasets/3.0.0/vdj_v1_hs_pbmc2_5gex_protein*
PBMCs FACS	10X GemCode Single-Cell 3' Library	10098	25389	Zheng et al., "Massively parallel digital transcriptional profiling of single cells", Nature Communications 14049 (2017)

Supplementary Table S1: Details on published scRNA-seq data sets used in the study.

Dataset	Minimum NODG	Maximum NODG	Maximum % mito	Minimum number of cells a gene must be expressed in
Cord Blood 10X	500	2000	8	100
Peripheral Blood Drop-Seq	500	2000	8	100
Lymphoid Tissue 10X	500	3000	30	100
Peripheral Blood 10X	500	3000	15	100
Peripheral Blood 10X-VDJ	500	4000	15	100
PBMCs FACS	300	2000	8	100

Supplementary Table S2: QC Metrics for data pre-processing. NODG: Number of Detected Genes

Variance threshold	CBMC	PBMC Drop-Seq	MALT	PBMC	PBMC-VDJ
0.5	1239	1457	826	866	*620*
1	348	471	82	75	*60*

Supplementary Table S3: Number of genes used for bootstrapping depending on the variance threshold.

Supplementary Notes

Supplementary Note 1 – Generation of an Immune Reference Panel

The immune reference panel was generated by downloading the bulk RNA sequencing data from GEO Accession Viewer: GSE107011 (Monaco et al. 2019). Cells derived from PBMCs and CD4_TE s were removed from all donors. In addition, VD2 positive cell from DZQV donor was removed. Quantile normalization was done using the preprocessCore R package. Genes with TPM greater and equal to 10 in at least 3 biological replicates were retained and log normalization with base 10 was performed. Batch correction using COMBAT (SVA R package) was done to remove the Batch effects. Gene expression for each gene was averaged across donors prior to feature gene identification. Lastly, feature genes were selected based on cell type specific expression in the dataset. A gene is included in the feature gene set if the fold change of its expression relative to other cell types is greater or equal to five.

```
options(stringsAsFactors=FALSE)
args=(commandArgs(TRUE))
optional_args <- c()
for(i in 1:length(args)) {
  eval(parse(text=args[[i]]))
  arg_str <- gsub("^(\\S+)=\\S+$", "\\1", args[[i]])
  if(
        arg_str == "with_replicates" ||
        arg_str == "is_bulk" ||
    arg_str == "detected_gene_cutoff" ||
    arg_str == "hq_genes_cutoff" ||
        arg_str == "log_transform" ||
    arg_str == "log_pseudo" ||
    arg_str == "log_base" ||
    arg_str == "log_pseudo_replace" ||
        arg_str == "normalization_method" ||
        arg_str == "fs_fc_cutoff" ||
        arg_str == "fs_quantile_probe"
 ) {
      optional_args <- c(optional_args, args[[i]])
  }
}

# args default value
with_replicates <- FALSE
is_bulk <- TRUE
detected_gene_cutoff <- 0
hq_genes_cutoff <- 2
log_transform <- TRUE
log_pseudo <- 1
log_base <- 10
```

```
log_pseudo_replace <- FALSE
normalization_method <- "quantile"
fs_fc_cutoff <- 10
fs_quantile_probe <- 0.5

# parsing optional args
optional_args_str <- paste0(optional_args, collapse=";")
eval(parse(text=optional_args_str))

# read TPM data
exprs <- readRDS(exprs_file)

######################################################
########## plot violin plot of TPM     #################
######################################################

beforenorm_violin_in <- exprs

if(log_transform) {
	if(log_pseudo_replace) {
		beforenorm_violin_in[beforenorm_violin_in < log_pseudo] <- log_pseudo
		beforenorm_violin_in <- log(beforenorm_violin_in, base=log_base)
	} else {
		beforenorm_violin_in <- log(beforenorm_violin_in + log_pseudo, base=log_base)
	}
}

if(!require("reshape2")) {
	install.packages("reshape2", repos="http://cran.stat.nus.edu.sg")
	require(reshape2)
}
beforenorm_violin_ggplot_in <- melt(as.data.frame(beforenorm_violin_in))
colnames(beforenorm_violin_ggplot_in) <- c("celltype", "value")

if(!require("ggplot2")) {
	install.packages("ggplot2", repos="http://cran.stat.nus.edu.sg")
	require("ggplot2")
}
pdf(paste0(output_dir, "/violin_beforenorm.pdf"), width=14, height=7)
ggplot(beforenorm_violin_ggplot_in, aes(x=celltype, y=value)) + geom_violin() +
geom_boxplot(width=.1, fill="red") + theme_bw() + theme(axis.text.x = element_text(angle = 90, hjust =
1)) + labs(y="log(expression)")
dev.off()

#######################################################
##########    quantile norm   ###########################
#######################################################
```

```
if (normalization_method=="quantile") {
	# quantile normalization
	if(!require("preprocessCore")) {
		source("http://bioconductor.org/biocLite.R")
		biocLite("preprocessCore")
		require(preprocessCore)
	}
	quantile_out <- normalize.quantiles.robust(as.matrix(exprs), use.median=TRUE)
	rownames(quantile_out) <- rownames(exprs)
	colnames(quantile_out) <- colnames(exprs)
} else if (normalization_method=="iQ") {
	quantile_out <- iQ_columnsorted_mediantarget_correctassignback(exprs)
} else {
	cat("Unknown normalization method, use normalize.quantiles.robust(, use.median=TRUE) from
preprocessCore ...\n")
	# quantile normalization
	if(!require("preprocessCore")) {
		source("http://bioconductor.org/biocLite.R")
		biocLite("preprocessCore")
		require(preprocessCore)
	}
	quantile_out <- normalize.quantiles.robust(as.matrix(exprs), use.median=TRUE)
	rownames(quantile_out) <- rownames(exprs)
	colnames(quantile_out) <- colnames(exprs)
}

#####################################################
##########	violin plot after quantile	###############
#####################################################

afternorm_violin_in <- quantile_out

if(log_transform) {
	if(log_pseudo_replace) {
		afternorm_violin_in[afternorm_violin_in < log_pseudo] <- log_pseudo
		afternorm_violin_in <- log(afternorm_violin_in, base=log_base)
	} else {
		afternorm_violin_in <- log(afternorm_violin_in + log_pseudo, base=log_base)
	}
}

afternorm_violin_ggplot_in <- melt(as.data.frame(afternorm_violin_in))
colnames(afternorm_violin_ggplot_in) <- c("celltype", "value")

pdf(paste0(output_dir, "/violin_afternorm.pdf"), width=14, height=7)
ggplot(afternorm_violin_ggplot_in, aes(x=celltype, y=value)) + geom_violin() + geom_boxplot(width=.1,
fill="red") + theme_bw() + theme(axis.text.x = element_text(angle = 90, hjust = 1)) +
labs(y="log(expression)")
```

```
dev.off()

######################################################
######### plot histogram of median         ##########
######### expression of gene across samples ##########
######################################################

median_of_genes<- apply(quantile_out,1,function(x) median(x[x>0]))

# plot the histogram to check the distribution of genes across
pdf(paste0(output_dir,"/histogram_medianofgeneexpressionacross_samples.pdf"), width=14, height=7)
hist(median_of_genes, breaks=10000, xlim=c(0,100), col="pink")
dev.off()

######################################################
########### find expressed genes       ##############
######################################################

print(sample_info_file)
sample_info <- read.table(sample_info_file, sep="\t", header=TRUE, as.is=TRUE, check.names=FALSE)
rownames(sample_info) <- sample_info$sample_id

# make sure that the gene expressed above TPM > detected_gene_cutoff in any cell type in at least 3
biological replicates
check_reps_gene_exprs<-t(apply(as.matrix(quantile_out), 1,function(v){aggregate(v,
by=list(celltype=sample_info[colnames(quantile_out), "celltype"]),
function(x){sum(x>=detected_gene_cutoff) >= hq_genes_cutoff})$x}))
colnames(check_reps_gene_exprs) <- aggregate(as.matrix(quantile_out)[1, ],
by=list(celltype=sample_info[colnames(quantile_out), "celltype"]), mean)$celltype

# get number of cell types
cell_types <- list(celltype=sample_info[colnames(quantile_out), "celltype"])$celltype
number_of_cell_types <- length(unique(cell_types))
print(number_of_cell_types)

# filter those transcripts/genes that fail the above criteria
# rows that have all FALSE across all cell types
# expressed_genes are genes that do not have FALSE for all 28 cell types
matrix_expressed <- quantile_out[which(apply(check_reps_gene_exprs, 1,function(x)sum(x==FALSE))!=
number_of_cell_types),]

#######################################################
##########     log transformation     #######################
#######################################################
if(log_transform) {
	if(log_pseudo_replace) {
		matrix_expressed[matrix_expressed < log_pseudo] <- log_pseudo
		quantile_log <- log(matrix_expressed, base=log_base)
```

```
	} else {
		quantile_log <- log(matrix_expressed + log_pseudo, base=log_base)
	}
}
saveRDS(quantile_log, file=paste0(output_dir, "/After_quantile_normalization.rds"))

######################################################
##########    COMBAT to correct for batch effects ############
#######################################################

library(sva)
batch <- factor(sample_info$sample_type)
celltype<-factor(sample_info$celltype)
modcombat <- model.matrix(~1+celltype, data=sample_info)
combat_edata <- ComBat(dat=quantile_log,batch=batch, mod=modcombat, par.prior =
TRUE,prior.plots=FALSE)
saveRDS(combat_edata, file=paste0(output_dir, "/After_combat_expression_data.rds"))

# plot a dendrogram to see the clsutering of samples after combat

pdf(paste0(output_dir,
"/all_expressed_genes_after_combat_before_choosing_featuregenes_hclust.pdf"), height=7, width=14)
plot(hclust(as.dist(1 - cor(combat_edata)), method="average"), xlab="hclust(as.dist(1 - cor(*)),
method=\"average\")", main=paste0("Number of genes: ", nrow(combat_edata)))
dev.off()

##################################################################################
##########      merge replicates on feature genes ######################################
##################################################################################

if(with_replicates) {
	sample_info <- read.table(sample_info_file, sep="\t", header=TRUE, as.is=TRUE,
check.names=FALSE)
	rownames(sample_info) <- sample_info$sample_id

	expr_gene_repmerge <- t(apply(as.matrix(combat_edata), 1,
		function(v){
			aggregate(v, by=list(celltype=sample_info[colnames(combat_edata), "celltype"]), mean)$x
		}
	))
	colnames(expr_gene_repmerge) <- aggregate(as.matrix(combat_edata)[1, ],
by=list(celltype=sample_info[colnames(combat_edata), "celltype"]), mean)$celltype
	saveRDS(expr_gene_repmerge, file=paste0(output_dir, "/expr_gene_repmerge_average.rds"))
}

#################################################################
##########      DE genes selection for ref panel ##################
#################################################################
```

```
logFC_thr <- log(fs_fc_cutoff, base=log_base)
tmp_binary <- t(apply(expr_gene_repmerge, 1, function(v){(v-quantile(v, probs=fs_quantile_probe)) >
logFC_thr})) + 0

foldchange <- t(apply(expr_gene_repmerge,1,function(v){(v-quantile(v, probs=fs_quantile_probe))}))
saveRDS(foldchange,file=paste0(output_dir, "/fold_change_aftermerge.rds"))

# plot the violin plot for fold change
foldchange_violin_ggplot_in <- melt(as.data.frame(foldchange))
colnames(foldchange_violin_ggplot_in) <- c("celltype", "value")
pdf(paste0(output_dir, "/violin_foldchange_aftermerge.pdf"), width=14, height=7)
ggplot(foldchange_violin_ggplot_in, aes(x=celltype, y=value)) + geom_violin() + geom_boxplot(width=.1,
fill="red") + theme_bw() + theme(axis.text.x = element_text(angle = 90, hjust = 1)) +
labs(y="foldchange")
dev.off()

featuregene_binary <- tmp_binary[apply(tmp_binary, 1, function(v){sum(v) > 0}), ]
expr_featuregene <- expr_gene_repmerge[rownames(featuregene_binary), ]

saveRDS(expr_featuregene, file=paste0(output_dir, "/expr_featuregene_aftermerge.RDS"))
saveRDS(featuregene_binary,  file=paste0(output_dir, "/featuregene_binary_aftermerge.RDS"))

pdf(paste0(output_dir, "/expr_featuregene_aftermerge_hclust.pdf"), height=7, width=14)
plot(hclust(as.dist(1 - cor(expr_featuregene)), method="average"), xlab="hclust(as.dist(1 - cor(*)),
method=\"average\")", main=paste0("Number of feature genes: ", nrow(expr_featuregene)))
dev.off()

library(gplots)
pdf(paste0(output_dir, "/expr_featuregene_aftermerge_selfprojection.pdf"), height=20, width=20)
color_scheme <- colorRampPalette(c("#00007F", "blue", "#007FFF", "cyan", "#7FFF7F", "yellow",
"#FF7F00", "red", "#7F0000"))(100)
heatmap_in <- cor(expr_featuregene)

if(with_replicates) {
	if (!require("WGCNA")) {
		source("http://bioconductor.org/biocLite.R")
		biocLite(c("AnnotationDbi", "impute", "GO.db", "preprocessCore"), suppressUpdates=TRUE)
		install.packages("WGCNA", repos="http://cran.stat.nus.edu.sg")
		require(WGCNA)
	}

	sample_info <- read.table(sample_info_file, sep="\t", header=TRUE, as.is=TRUE,
check.names=FALSE)
	rownames(sample_info) <- sample_info$sample_id

	heatmap.2(heatmap_in,col=color_scheme,
		Colv=as.dendrogram(hclust(as.dist(1 - cor(heatmap_in)), method="average")),
```

```
            Rowv=as.dendrogram(hclust(as.dist(1 - cor(heatmap_in)), method="average")),
            RowSideColors=labels2colors(sample_info[colnames(heatmap_in), "celltype"]),
            scale="none", margins=c(5,20),
            trace="none",
            key = TRUE,
            keysize = 0.5,
            cexCol = 1,cexRow =1,
            labCol = "",
            main = paste0("Number of feature genes: ", nrow(expr_featuregene))
        )
        dev.off()
} else {
        heatmap.2(heatmap_in,col=color_scheme,
            Colv=as.dendrogram(hclust(as.dist(1 - cor(heatmap_in)), method="average")),
            Rowv=as.dendrogram(hclust(as.dist(1 - cor(heatmap_in)), method="average")),
            scale="none", margins=c(5,20),
            trace="none",
            key = TRUE,
            keysize = 0.5,
            cexCol = 1,cexRow =1,
            labCol = "",
            main = paste0("Number of feature genes: ", nrow(expr_featuregene))
        )
        dev.off()
}

library(gplots)
pdf(paste0(output_dir, "/featuregene_binary_aftermerge.pdf"), height=20, width=20)
heatmap.2(featuregene_binary,
        Colv=as.dendrogram(hclust(as.dist(1 - cor(expr_featuregene)), method="average")),
        scale="none", margins=c(20,20),
        trace="none",
        dendrogram="column",
        key = TRUE,
        keysize = 0.5,
        cexCol = 1.5,cexRow = 0.1,
        lhei=c(0.2,2)
)
dev.off()
```

Supplementary Note 2 – Preprocessing and scConsensus Execution for CITE-Seq data

Function to compute DE gene refinement

#'
#'@author: ranjanb
#'
#'@description: ComputeDatasetGroundTruth() takes in a dataMatrix and clusterLabels and outputs the DE gene
#'heatmap along with a data object containing the q-values, log2-fold changes, DE genes, union of DE genes, DE gene
#'counts and the up and down regulated DE genes for each pairwise comparison.
#'
#'@inputs:
#'matrix dataMatrix: genes (rows) x cells (columns) log10 transformed gene expression matrix
#'named character vector clusterLabels: vector of cluster labels, named using corresponding cell IDs (ordered as columns of dataMatrix)
#'character method: defines the method to be used to compute DE genes, can take the value of "Wilcoxon", "LimmaVoom" or "edgeR". default = "Wilcoxon".
#'numeric meanScalingFactor: scale of the mean gene expression across the gene expression matrix to set a minimum threshold of average cluster expression for a gene to be considered a DE gene
#'numeric qValThrs: maximum q-value threshold for Wilcoxon Rank Sum Test
#'numeric fcThrs: minimum fold-change threshold for DE gene criterion
#'vector deepSplitValues: vector of WGCNA tree cutting deepsplit parameters
#'#'character minClusterSizeType: specifies the type of minimum cluster factor the user would like to specify: "scaled" for a fraction of the number of cells OR "absolute" for the absolute value of minimum number of cells. default = "scaled"
#'integer minClusterFactor: factor to determine minimum number of cells in each cluster.
#'character dataType: specifies if the type of data is 10x or not, and hence if it requires additional normalization or not
#'character folderOut: specifies the output directory of the results
#'
#'@output:
#'returnObj = list(
#'list qValueList: list of q-values for each pairwise cluster comparison
#'list log2FCList: list of log2-fold-changes for each pairwise cluster comparison
#'list deGeneList: list of de genes for each pairwise cluster comparison
#'character vector deGeneUnion: union of all DE genes
#'numeric matrix deCountMatrix: matrix with number of DE genes for each pairwise cluster comparison
#'list deGeneRegulationList: list of up and down regulated DE genes
#'list upregulatedDEGeneList: list of cluster-specific upregulated DE genes
#'list downregulatedDEGeneList: list of cluster-specific downregulated DE genes
#'list cellTree: dendrogram of cells clustered in deGeneUnion space
#'list dynamicColors: list of cluster labels based on clustering in deGeneUnion space over all deepSplitValues
#')

```
#'

# source("R:/CSB/CSB6/Private/Bobby/rna-seq-pipelines/cellTypeDEPlot.R")
# source("R:/CSB/CSB6/Private/Bobby/rna-seq-pipelines/plotVolcano.R")

ComputeDatasetGroundTruth <- function(dataMatrix,
                                      clusterLabels,
                                      method = "Wilcoxon",
                                      meanScalingFactor = 5,
                                      qValThrs,
                                      fcThrs,
                                      deepSplitValues = 1:4,
                                      minClusterSizeType = "scaled",
                                      minClusterFactor = 100,
                                      dataType = "10x",
                                      folderOut = ".") {
  ### Check if package dependencies are available; if not, download from CRAN and require those
packages
  # Seurat
  if (!require(Seurat))
    install.packages("Seurat", repos = "http://cran.us.r-project.org")
  require(Seurat)

  # flashClust
  if (!require(flashClust))
    install.packages("flashClust", repos = "http://cran.us.r-project.org")
  require(flashClust)

  # calibrate
  if (!require(calibrate))
    install.packages("calibrate", repos = "http://cran.us.r-project.org")
  require(calibrate)

  # WGCNA
  if (!require(WGCNA)) {
    source("http://bioconductor.org/biocLite.R")

    biocLite(c("impute", "GO.db", "preprocessCore"))

    install.packages("WGCNA")

  }
  require(WGCNA)

  # Limma
  if (!require(limma)) {
    if (!requireNamespace("BiocManager", quietly = TRUE))
      install.packages("BiocManager")
```

```
    BiocManager::install("limma", version = "3.8")
  }
  require(limma)

  # edgeR
  if (!require(edgeR)) {
    if (!requireNamespace("BiocManager", quietly = TRUE))
      install.packages("BiocManager")
    BiocManager::install("edgeR", version = "3.8")
  }
  require(edgeR)

  ### Initialise data input
  dataIn <- as.matrix(dataMatrix)

  ### Initialize minimum cluster size
  if (minClusterSizeType == "scaled") {
    minClusterSize = ceiling(ncol(dataIn) / minClusterFactor)
  } else if (minClusterSizeType == "absolute") {
    minClusterSize = minClusterFactor
  } else {
    minClusterSize = 10
  }


  ### 10x data needs to be log normalized by cell using Seurat's LogNormalize method to reduce ties in
data before calling DE genes
  if (dataType == "10x") {
    ### Log Normalize using Seurat
    seuratObj <- CreateSeuratObject(counts = dataIn)
    seuratObj <-
      NormalizeData(
        object = seuratObj,
        normalization.method = "LogNormalize",
        scale.factor = 10000
      )

    ### Return normalized data to data matrix
    dataIn <- as.matrix(seuratObj@assays$RNA@data)

    ### Convert log base from e to 2
    dataIn <- dataIn / log(2)
  } else {
    ### Convert log base from e to 2
    dataIn <- dataIn / log(2)
  }
```

```
### Initialize mean expression threshold
meanExprsThrs = meanScalingFactor * mean(2^dataIn-1)

### Use only clusters with number of cells > minimum cluster size for DE gene calling
which(table(clusterLabels) > minClusterSize)
colorCounts <-
  table(clusterLabels)

### Extract unique cluster labels
uniqueClusters <-
  names(colorCounts[colorCounts > minClusterSize])

# ignore the grey cluster because it represents unclustered cells
uniqueClusters <-
  uniqueClusters[!grepl("grey", uniqueClusters)]

### If the cluster label vector is unnamed, name it in the order of the data matrix columns
if (is.null(names(clusterLabels))) {
  names(clusterLabels) <- colnames(dataIn)
}

### Initialize nested lists to store q values, log-normalized fold change values and de genes
qValueList <-
  rep(list(list()), length(uniqueClusters))
log2FCList <-
  rep(list(list()), length(uniqueClusters))
deGeneList <- rep(list(list()), length(uniqueClusters))

### Initialise number of comparisons as n(n-1)/2
numComparisons <-
  (length(uniqueClusters) * (length(uniqueClusters) - 1)) / 2

### Conduct pairwise cluster comparison to obtain q-values, log-normalized fold changes and
### DE genes for each comparison
for (i in 1:(length(uniqueClusters) - 1)) {
  for (j in (i + 1):length(uniqueClusters)) {
    ### Get the cell names cell data for cluster i
    cellNamesi <-
      names(clusterLabels)[which(clusterLabels == uniqueClusters[i])]
    cellDatai <- dataIn[, cellNamesi]

    ### Get the cell names cell data for cluster j
    cellNamesj <-
      names(clusterLabels)[which(clusterLabels == uniqueClusters[j])]
    cellDataj <- dataIn[, cellNamesj]

    deCellData <- cbind(cellDatai, cellDataj)
```

```
    ### Declare variables to store p-values, q-values and log2 fold change values for each pairwise
gene comparison
    pval <- NA
    qval <- NA
    log2fc <- NA

    if (method == "Wilcoxon") {
      ### For each gene, conduct Wilcoxon Rank Sum test to obtain q-value and log2-normalized fold
change
      for (k in 1:nrow(cellDatai)) {

        # extract data of gene k for cluster i
        geneDatai <- cellDatai[k, ]

        # extract data of gene k for cluster j
        geneDataj <- cellDataj[k, ]

        # run wilcoxon test
        wcTestOut <-
          wilcox.test(geneDatai, geneDataj)

        # initialize q value and fold change for each gene
        pval[k] <- wcTestOut$p.value
        log2fc[k] <-
          mean(geneDatai) - mean(geneDataj)
      }

      # check if gene satisfies mean expression threshold
      meanExprsLogicalVector <-
        apply(deCellData, 1, function(row) {
          mean(row[cellNamesi]) > log2(meanExprsThrs) |
            mean(row[cellNamesj]) > log2(meanExprsThrs)
        })

      # adjust p-value using Benjamini-Hochberg adjustment
      qval <-
        p.adjust(
          p = pval,
          method = "BH",
          n = nrow(cellDatai)
        )

    } else if (method == "LimmaVoom") {
      # initialise vector of 1s to denote cells of cluster i and -1s to denote cells of cluster j
      limmaGroupi <-
        rep(uniqueClusters[i], ncol(cellDatai))
      limmaGroupj <-
        rep(uniqueClusters[j], ncol(cellDataj))
```

```
    limmaGroup <-
      c(limmaGroupi, limmaGroupj)

    dge <-
      DGEList(deCellData, group = limmaGroup)
    dge <- calcNormFactors(dge)
    design <- model.matrix(~ limmaGroup)
    vm <- voom(dge, design = design, plot = TRUE)

    fit <- lmFit(vm, design = design)
    fit <- eBayes(fit)
    tt <-
      topTable(fit = fit,
               n = Inf,
               adjust.method = "BH")

    # # compute p-value using limma-voom fitting & eBayes method
    # pval <- apply(deCellData, 1, function(v) {
    #     fit <- lmFit(v, limmaGroup)
    #     return(eBayes(fit, trend = TRUE)$p.value)
    # })

    # adjust p-value using Benjamini-Hochberg adjustment
    # qval <-
    #     p.adjust(
    #         p = pval,
    #         method = "BH",
    #         n = numComparisons * nrow(cellDatai)
    #     )

    qval <- tt$adj.P.Val

    # compute q-value in log space by finding difference of means
    log2fc <-
      apply(deCellData, 1, function(row) {
        mean(row[cellNamesi]) - mean(row[cellNamesj])
      })
    # check if gene satisfies mean expression threshold
    meanExprsLogicalVector <-
      apply(deCellData, 1, function(row) {
        mean(row[cellNamesi]) > log2(meanExprsThrs) |
          mean(row[cellNamesj]) > log2(meanExprsThrs)
      })

  } else if (method == "edgeR") {

    # Create DGE object
    dgeObj <- DGEList(counts=deCellData, group = c(rep(1, ncol(cellDatai)), rep(-1, ncol(cellDataj))))
```

```
    # Estimate common and tag-wise dispersion for the pair of clusters
    dgeObj <- estimateCommonDisp(dgeObj)
    dgeObj <- estimateTagwiseDisp(dgeObj)

    # Calculate norm factors for data
    dgeObj <- calcNormFactors(dgeObj)

    # Perform exact test
    etObj <- exactTest(object = dgeObj)

    # Save p-value results
    pval <- etObj$table$PValue

    # adjust p-value using Benjamini-Hochberg adjustment
    qval <-
      p.adjust(
        p = pval,
        method = "BH",
        n = nrow(cellDatai)
      )

    log2fc <- etObj$table$logFC



    # check if gene satisfies mean expression threshold
    meanExprsLogicalVector <-
      apply(deCellData, 1, function(row) {
        mean(row[cellNamesi]) > log2(meanExprsThrs) |
          mean(row[cellNamesj]) > log2(meanExprsThrs)
      })

  } else {
    ### exit from function if no method is chosen
    print("Incorrect method chosen.")
    return(NULL)
  }

  ### determine if a gene is a DE gene based on thresholds
  deGeneLogicalVector <-
    qval < qValThrs &
    abs(log2fc) > log2(fcThrs)

  # filter de genes by mean expression
  deGeneLogicalVector <- deGeneLogicalVector & meanExprsLogicalVector

  print(paste0(
```

```
        uniqueClusters[i],
        ", ",
        uniqueClusters[j],
        " DE genes: ",
        sum(deGeneLogicalVector)
      ))

      ### store q-values, log-normalized fold change and DE genes
      qValueList[[i]][[j]] <- qval
      log2FCList[[i]][[j]] <- log2fc
      deGeneList[[i]][[j]] <-
        rownames(cellDatai)[deGeneLogicalVector]

      if (sum(deGeneLogicalVector) <= 1)
        next

      de_genes <- deGeneList[[i]][[j]]

  }
}

### Name the q-value, log2 fold change and DE gene lists by cluster names
names(qValueList) <- uniqueClusters
names(log2FCList) <- uniqueClusters
names(deGeneList) <- uniqueClusters

### Obtain union of all de genes
# initialize empty DE gene union vector
deGeneUnion <- c()

# For each pair of clusters
for (i in 1:(length(uniqueClusters) - 1)) {
  for (j in (i + 1):length(uniqueClusters)) {
    # obtain the union of the current DE gene union so far with the next DE gene list
    deGeneUnion <-
      union(deGeneUnion, deGeneList[[i]][[j]])


    # Rank the DE genes for each comparison by fold change and take the top 20

    # de_log2fc <- log2FCList[[i]][[j]][which(rownames(dataIn) %in% deGeneList[[i]][[j]])]
    # names(de_log2fc) <- rownames(dataIn)[which(rownames(dataIn) %in% deGeneList[[i]][[j]])]
    #
    # sorted_de_log2fc <- sort(x = abs(de_log2fc), decreasing = T)
    #
    # if(length(sorted_de_log2fc) > 20) {
    #    de_genes <- names(sorted_de_log2fc)[1:20]
    # } else {
```

```
    #     de_genes <- names(sorted_de_log2fc)
    # }
    #
    # deGeneUnion <-
    #     union(deGeneUnion, de_genes)

  }
}

print(str(deGeneUnion))

### Make DE gene matrix symmetric, as it is currrently an upper triangular matrix
# First cover every element barring the last row and last column
for (i in 1:(length(uniqueClusters) - 1)) {
  for (j in 1:(length(uniqueClusters) - 1)) {
    # clusters do not have DE genes with themselves, so skip to next iteration
    if (i == j) {
      next
    }

    # if an (i,j) pair is empty, fill it with the corresponding (j,i) DE genes
    if (is.null(deGeneList[[i]][[j]])) {
      deGeneList[[i]][[j]] <- deGeneList[[j]][[i]]
    }

  }

  # Name the DE gene list with cluster names
  names(deGeneList[[i]]) <- uniqueClusters
}


# For the last row and last column
for (i in 1:(length(uniqueClusters) - 1)) {
  # set the DE genes of (last_row, i) as DE genes (i, last_row)
  deGeneList[[length(uniqueClusters)]][[i]] <-
    deGeneList[[i]][[length(uniqueClusters)]]

  # name the DE gene list with cluster names
  names(deGeneList[[length(uniqueClusters)]])[i] <-
    uniqueClusters[i]
}


## Make the log2 fold change matrix symmetric, as it is currrently an upper triangular matrix
# First cover every element barring the last row and last column
for (i in 1:(length(uniqueClusters) - 1)) {
  for (j in 1:(length(uniqueClusters) - 1)) {
```

```
        # clusters do not have DE genes with themselves, so skip to next iteration
        if (i == j) {
            next
        }

        # if an (i,j) pair is empty, fill it with negation of the corresponding (j,i) fold change values
        if (is.null(log2FCList[[i]][[j]])) {
            log2FCList[[i]][[j]] <- -log2FCList[[j]][[i]]
        }

    }

    # name the log2 fold change list with cluster names
    names(log2FCList[[i]]) <- uniqueClusters
}

# For the last row and last column
for (i in 1:(length(uniqueClusters) - 1)) {
    # set the log2 fold changes of (last_row, i) as the negation of log2 fold changes of (i, last_row)
    log2FCList[[length(uniqueClusters)]][[i]] <-
        -log2FCList[[i]][[length(uniqueClusters)]]

    # name the log2 fold change list with cluster names
    names(log2FCList[[length(uniqueClusters)]])[i] <-
        uniqueClusters[i]
}


## Make the q values matrix symmetric, as it is currrently an upper triangular matrix
# First cover every element barring the last row and last column
for (i in 1:(length(uniqueClusters) - 1)) {
    for (j in 1:(length(uniqueClusters) - 1)) {
        # clusters do not have DE genes with themselves, so skip to next iteration
        if (i == j) {
            next
        }

        # if an (i,j) pair is empty, fill it with the corresponding (j,i) q-values
        if (is.null(qValueList[[i]][[j]])) {
            qValueList[[i]][[j]] <- qValueList[[j]][[i]]
        }

    }

    # name the q-value list with cluster names
    names(qValueList[[i]]) <- uniqueClusters
}
```

```
# For the last row and last column
for (i in 1:(length(uniqueClusters) - 1)) {
  # set the q-values of (last_row, i) as the q-values of (i, last_row)
  qValueList[[length(uniqueClusters)]][[i]] <-
    qValueList[[i]][[length(uniqueClusters)]]

  # name the q-value list with cluster names
  names(qValueList[[length(uniqueClusters)]])[i] <-
    uniqueClusters[i]
}


### Construct de gene count matrix
# initialise zeroes matrix
deCountMatrix <-
  matrix(
    data = 0,
    nrow = length(uniqueClusters),
    ncol = length(uniqueClusters)
  )

# name the de gene count matrix with cluster names
rownames(deCountMatrix) <- uniqueClusters
colnames(deCountMatrix) <- uniqueClusters

# for each pair of clusters
for (i in 1:length(uniqueClusters)) {
  for (j in 1:length(uniqueClusters)) {
    # clusters do not have DE genes with themselves, so count = 0
    if (i == j) {
      deCountMatrix[i, j] <- 0
    }
    # number of DE genes in (last_row, j) = number of elements in the (j, last_row)
    else if (i == length(uniqueClusters)) {
      deCountMatrix[i, j] <- length(deGeneList[[j]][[i]])
    }
    # if DE gene list is empty, count = 0
    else if (is.null(deGeneList[[i]][[j]])) {
      deCountMatrix[i, j] <- 0
    }
    # else, DE gene count (i,j) = number of DE genes at (i,j)
    else {
      deCountMatrix[i, j] <- length(deGeneList[[i]][[j]])
    }
  }
}

### Construct de gene list with up/down regulatory information
```

```
# initialize empty list of lists
deGeneRegulationList <-
  rep(list(list()), length(uniqueClusters))

# name DE gene regulation list with cluster names
names(deGeneRegulationList) <- uniqueClusters

# For each pair of clusters
for (i in 1:length(uniqueClusters)) {
  for (j in 1:length(uniqueClusters)) {
    # clusters do not have DE genes with themselves, so skip to next iteration
    if (i == j) {
      next
    }

    # store fold change vector for clusters (i,j)
    fcVec <- log2FCList[[i]][[j]]

    # name fold change vector with gene names
    names(fcVec) <- rownames(dataIn)

    # extract fold changes for DE genes of (i,j)
    fcVec <- fcVec[deGeneList[[i]][[j]]]

    # obtain up and down regulated DE genes from DE gene fold change vector
    upDEGenes <- names(which(fcVec > 0))
    downDEGenes <- names(which(fcVec < 0))

    # save up and down regulated genes in list
    deGeneRegulationList[[i]][[j]] <-
      list(
        "upregulated_de_genes" = upDEGenes,
        "downregulated_de_genes" = downDEGenes
      )

    # name DE gene regulation list with cluster names
    names(deGeneRegulationList[[i]])[j] <-
      uniqueClusters[j]
  }
}

### For each cluster, obtain intersection of upregulated and downregulated DE genes against every
other cluster
upregulatedDEGeneList <-
  rep(list(), length(deGeneRegulationList))

downregulatedDEGeneList <-
  rep(list(), length(deGeneRegulationList))
```

```
    for (i in 1:length(deGeneRegulationList)) {
        # initialise empty list for each cluster
        upregulatedDEGeneList[[names(deGeneRegulationList)[i]]] <-
          c()

        downregulatedDEGeneList[[names(deGeneRegulationList)[i]]] <-
          c()

        for (j in 1:length(deGeneRegulationList[[i]])) {
          # clusters do not have DE genes with themselves, so skip to next iteration
          if (i == j)
            next

          # obtain the union of existing upregulated DE genes for that cluster with upregulated DE genes
  for cluster pair (i,j)
          upregulatedDEGeneList[[names(deGeneRegulationList)[i]]] <-
            union(upregulatedDEGeneList[[names(deGeneRegulationList)[i]]],
                  deGeneRegulationList[[i]][[j]]$upregulated_de_genes)

          # obtain the union of existing upregulated DE genes for that cluster with upregulated DE genes
  for cluster pair (i,j)

          downregulatedDEGeneList[[names(deGeneRegulationList)[i]]] <-
            union(
              downregulatedDEGeneList[[names(deGeneRegulationList)[i]]],
              deGeneRegulationList[[i]][[j]]$downregulated_de_genes
            )
        }

        for (j in 1:length(deGeneRegulationList[[i]])) {
          # clusters do not have DE genes with themselves, so skip to next iteration
          if (i == j)
            next

          # obtain the intersection of existing upregulated DE genes for that cluster with upregulated DE
  genes for cluster pair (i,j)
          upregulatedDEGeneList[[names(deGeneRegulationList)[i]]] <-
            intersect(upregulatedDEGeneList[[names(deGeneRegulationList)[i]]],
                      deGeneRegulationList[[i]][[j]]$upregulated_de_genes)

          # obtain the intersection of existing upregulated DE genes for that cluster with upregulated DE
  genes for cluster pair (i,j)
          downregulatedDEGeneList[[names(deGeneRegulationList)[i]]] <-
            intersect(
              downregulatedDEGeneList[[names(deGeneRegulationList)[i]]],
              deGeneRegulationList[[i]][[j]]$downregulated_de_genes
            )
```

```
    }

    # name upregulated DE gene list with cluster names
    upregulatedDEGeneList[[names(deGeneRegulationList)[i]]] <-
      unique(upregulatedDEGeneList[[names(deGeneRegulationList)[i]]])

    # name upregulated DE gene list with cluster names
    downregulatedDEGeneList[[names(deGeneRegulationList)[i]]] <-
      unique(downregulatedDEGeneList[[names(deGeneRegulationList)[i]]])
  }


  ### Create and initialise object to return to function caller
  returnObj = list(
    "qValueList" = qValueList,
    "log2FCList" = log2FCList,
    "deGeneList" = deGeneList,
    "deGeneUnion" = deGeneUnion,
    "deCountMatrix" = deCountMatrix,
    "deGeneRegulationList" = deGeneRegulationList,
    "upregulatedDEGeneList" = upregulatedDEGeneList,
    "downregulatedDEGeneList" = downregulatedDEGeneList
    # "cellTree" = cellTree,
    # "dynamicColors" = dynamicColorsList
  )

  save(returnObj, file = paste0(folderOut, "/", "de_gene_object.rds"))

  ### Return object
  return(returnObj)

}
```

```
# Redefine groundtruth
# Seurat 3.1

setwd("R:/CSB/CSB6/Private/Bobby/rna-seq-pipelines/")

library(stringr)
library(gplots)
library(RColorBrewer)
library(irlba)
library(Seurat)
library(RCAv2)
library(dplyr)
library(mclust)

##################################################CBMC#############################
##############################################
# load the original data
data_original = read.csv("../feature_selection/raw_datasets/GSE100866_CBMC_8K_13AB_10X-
RNA_umi.csv.gz",sep=",",header=T,row.names=1)
cbmc.rna.collapsed <- CollapseSpeciesExpressionMatrix(data_original)
cbmc <- CreateSeuratObject(cbmc.rna.collapsed, min.cells = 100, min.features = 500, project = "CBMC")
mito.features <- grep(pattern = "^MT-", x = rownames(x = cbmc), value = TRUE)
percent.mito <- Matrix::colSums(x = GetAssayData(object = cbmc, slot = 'counts')[mito.features, ]) /
Matrix::colSums(x = GetAssayData(object = cbmc, slot = 'counts'))
cbmc[['percent.mito']] <- percent.mito
VlnPlot(object = cbmc, features = c("nFeature_RNA", "nCount_RNA", "percent.mito"), ncol = 3)
cbmc <- subset(x = cbmc, subset = nFeature_RNA > 500 & nFeature_RNA < 2000 & percent.mito < 0.08)
cbmc <- NormalizeData(cbmc)
cbmc <- FindVariableFeatures(cbmc,selection.method = "vst",nfeatures = 1000)
cbmc <- ScaleData(cbmc, display.progress = FALSE)
cbmc <- RunPCA(cbmc,features = VariableFeatures(object = cbmc))
ElbowPlot(cbmc)
cbmc <- FindNeighbors(object = cbmc, reduction = "pca", dims = 1:15)
cbmc <- FindClusters(cbmc, resolution = 0.2)
cbmc <- RunTSNE(cbmc, dims = 1:15)

cbmc.markers <- FindAllMarkers(object = cbmc, only.pos = TRUE, min.pct = 0.25, logfc.threshold = 0.25)
cbmc.markers %>% group_by(cluster) %>% top_n(n = 2, wt = avg_logFC) %>% print(n=26)

new.cluster.ids <- c("CD4 T", "CD14+ Mono", "NK", "Mouse","CD8 T", "FCGR3A+ Mono","B",
                     "Mk", "Precursors", "DC","Eryth")
names(x = new.cluster.ids) <- levels(x = cbmc)
cbmc <- RenameIdents(object = cbmc, new.cluster.ids)
DimPlot(object = cbmc, reduction = 'tsne', label = TRUE, pt.size = 0.5)
# DoHeatmap(object = cbmc, features = cbmc.markers$gene, slot = "data", size = 3)
table(cbmc@active.ident)
cbmc[["rna_11clusters"]] <- Idents(object = cbmc)
```

```
# load the ADT UMI data
cbmc.adt <- read.csv("../feature_selection/raw_datasets/GSE100866_CBMC_8K_13AB_10X-
ADT_umi.csv",sep = ",", header = TRUE, row.names = 1)
cbmc.adt = cbmc.adt[,names(cbmc@active.ident)]
cbmc.adt = cbmc.adt[setdiff(rownames(cbmc.adt),c("CCR5","CCR7","CD10")),]
cbmc.cite <- CreateSeuratObject(counts = cbmc.adt)
cbmc.cite = NormalizeData(object = cbmc.cite, method = "CLR")
cbmc.cite <- ScaleData(cbmc.cite, features = NULL, display.progress = FALSE)

###### cluster based on ADT data
# Cluster based on PCA
cbmc.cite <- RunPCA(cbmc.cite, features = rownames(cbmc.cite@assays$RNA))
cbmc.cite <- FindNeighbors(object = cbmc.cite, reduction = "pca", dims = 1:9)
cbmc.cite <- FindClusters(cbmc.cite, resolution = 0.3, Reduction.type = "pca")
cbmc.cite[["rna_11clusters"]] <- Idents(object = cbmc)
adt_pca_labels = cbmc.cite@active.ident
cbmc.cite <- RunTSNE(cbmc.cite, dims = 1:9)
DimPlot(object = cbmc.cite, reduction = 'tsne', label = TRUE, pt.size = 0.5)
cbmc.small <- SubsetData(cbmc.cite, max.cells.per.ident = 300)
adt.markers <- FindAllMarkers(cbmc.small, only.pos = TRUE)
DoHeatmap(cbmc.small, features = unique(adt.markers$gene),size=3)
DoHeatmap(cbmc.small, features = unique(adt.markers$gene),size=3,group.by = "rna_11clusters")

# Cluser based on UMAP
cbmc.cite <- RunUMAP(cbmc.cite,features = rownames(cbmc.cite@assays$RNA))
cbmc.cite <- FindNeighbors(object = cbmc.cite, reduction = "umap", dims = 1:2)
cbmc.cite <- FindClusters(cbmc.cite, resolution = 0.05, Reduction.type = "umap")
DimPlot(object = cbmc.cite, reduction = 'umap', label = TRUE, pt.size = 0.5)
cbmc.small <- SubsetData(cbmc.cite, max.cells.per.ident = 300)
adt.markers <- FindAllMarkers(cbmc.small, only.pos = TRUE)
DoHeatmap(cbmc.small, features = unique(adt.markers$gene),size=3)

# load data after filtering
data=benchmarkDataList$CBMC # in normal domain after filtering and normalization

# Intersection of data and the original data
data = data[, colnames(data) %in% colnames(cbmc@assays$RNA@data)]

# Run RCA global panel to label cells
# data_obj = dataConstruct(as.matrix(data))
# data_obj = geneFilt(obj_in = data_obj)
# data_obj = cellNormalize(data_obj)
# data_obj = dataTransform(data_obj)
# data_obj = featureConstruct(data_obj,method = "GlobalPanel",power = 4)
#
# data_obj = cellClust(data_obj,deepSplit_wgcna=0)
# RCAPlot(data_obj)
```

```
# Run RCA Monaco panel to label cells
data_obj = createRCAObject(rawData = log(1+data))
data_obj = dataProject(rca.obj = data_obj, method = "Custom", customPath = "../immune
panels/Custom_panel_Monaco_TPM10_absolutefc5_20190320/global_custom/global_monaco_panel.r
ds")
data_obj = dataClust(rca.obj = data_obj, deepSplitValues = 1:4, minClustSize = 10)

plotRCAHeatmap(data_obj, folderpath = "../CSHL Single Cell Analyses/CBMC/")
plotRCAUMAP(data_obj, folderpath = "../CSHL Single Cell Analyses/CBMC/")

#get the seurat label for filtered data
groundtruth = data.frame(RCA_labels = data_obj$clustering.out$dynamicColorsList$`deepSplit 2`)
groundtruth$seurat_labels = cbmc@active.ident[colnames(data)]
groundtruth$adt_umap_labels = cbmc.cite@active.ident[colnames(data)]
groundtruth$adt_pca_labels = adt_pca_labels[colnames(data)]

#Umap using RCA global panel clustering
seed = 42
# seurat_obj = CreateSeuratObject(data_obj$fpkm_for_clust)
# seurat_obj <- ScaleData(object = seurat_obj, display.progress = FALSE)
#
# seurat_obj = RunUMAP(obj=seurat_obj,features = rownames(seurat_obj@assays$RNA@data))
# seurat_obj@active.ident = as.factor(data_obj$group_labels_color$groupLabel)
# names(seurat_obj@active.ident) = colnames(data)
#
# colors = as.character(unique(data_obj$group_labels_color$dynamicColors))
# labels = unique(data_obj$group_labels_color$groupLabel)
# colors_sorted = colors[sort.int(labels,index.return = T)$ix]
# DimPlot(object = seurat_obj, reduction = 'umap',group.by = "ident",cols = colors_sorted)

# label using seurat labels
seed = 42
seurat_obj@active.ident = as.factor(groundtruth$seurat_labels)
names(seurat_obj@active.ident) = colnames(data)

colors = unique(labels2colors(groundtruth$seurat_labels))
DimPlot(object = seurat_obj, reduction = 'umap',group.by = "ident",cols = colors)

# label using ADT labels
seurat_obj@active.ident = as.factor(groundtruth$adt_umap_labels)
names(seurat_obj@active.ident) = colnames(data)

colors = unique(labels2colors(groundtruth$adt_umap_labels))
DimPlot(object = seurat_obj, reduction = 'umap',group.by = "ident",cols = colors)

# source('/mnt/volume1/project/plotHeatmap.R')
```

```
# cellTypeDEPlot(data_obj$fpkm_for_clust,cellTree = data_obj$cellTree,clusterLabels =
data_obj$group_labels_color$groupLabel,dynamicColors = data_obj$dynamicColors,filename =
"heatmap_RCA_Monacopanel_deepsplit2")

# Find the consensus groundtruth based on RCA and Seurat clustering
groundtruth = as.data.frame(data_obj$clustering.out$dynamicColorsList$`deepSplit 2`)
rownames(groundtruth) = colnames(data)
colnames(groundtruth) = "RCA"
groundtruth$RNA = data_obj$seurat_labels
groundtruth$ADT_Umap = data_obj$adt_umap_labels
groundtruth$ADT_pca = data_obj$adt_pca_labels

groundtruth$consensus = 0
groundtruth[groundtruth$RCA == 1,]$consensus = "T"
groundtruth[groundtruth$ADT_pca==0 & groundtruth$RCA == 1,]$consensus = "CD4 T Naive"
groundtruth[groundtruth$ADT_pca==3 & groundtruth$RCA == 1,]$consensus = "CD4 T Memory"
groundtruth[groundtruth$ADT_pca==6 & groundtruth$RCA == 1,]$consensus = "CD8 T"

groundtruth[groundtruth$RCA == 2,]$consensus = "Mono"
groundtruth[groundtruth$ADT_pca==1 & groundtruth$RCA == 2,]$consensus = "Mono"
groundtruth[groundtruth$ADT_pca==8 & groundtruth$RCA == 2,]$consensus = "CD16 Mono"
groundtruth[groundtruth$ADT_pca==9 & groundtruth$RCA == 2,]$consensus = "CD14 Mono"

groundtruth[groundtruth$RCA == 3,]$consensus = "NK"
groundtruth[groundtruth$ADT_Umap==2 & groundtruth$RCA == 3,]$consensus = "NK"
groundtruth[groundtruth$ADT_Umap==11 & groundtruth$RCA == 3,]$consensus = "NK T"

groundtruth[groundtruth$RCA == 4,]$consensus = "B"
groundtruth[groundtruth$ADT_pca==7 & groundtruth$RCA == 4,]$consensus = "B"
groundtruth[groundtruth$ADT_pca==11 & groundtruth$RCA == 4,]$consensus = "CD3+ B"

groundtruth[groundtruth$RCA == 5,]$consensus = "Precursors"
groundtruth[groundtruth$ADT_pca==10 & groundtruth$RCA == 5,]$consensus = "Precursors"

groundtruth[groundtruth$RCA == 6,]$consensus = "DC"

groundtruth[groundtruth$RCA == 7,]$consensus = "Myeloid-like NK"
groundtruth[groundtruth$ADT_Umap==8 & groundtruth$RCA == 7,]$consensus = "Myeloid-like NK"

groundtruth[groundtruth$RCA == 8,]$consensus = "Eryth"
groundtruth[groundtruth$RCA == 10,]$consensus = "Mk"
dim(groundtruth[groundtruth$consensus==0,])

groundtruthDataList = list()
groundtruthDataList[["CBMC"]] = data[,rownames(groundtruth[groundtruth$consensus!=0,])]
groundtruthGroupList = list()
groundtruthGroupList[["CBMC"]] = groundtruth[groundtruth$consensus!=0,]$consensus
```

```
save(groundtruthDataList,groundtruthGroupList,file="groundtruth_CBMC.RData")


# Find the consensus groundtruth based on RCA and Seurat clustering
groundtruth = as.data.frame(data_obj$group_labels_color$groupLabel)
rownames(groundtruth) = colnames(data)
colnames(groundtruth) = "RCA"
groundtruth$RNA = data_obj$seurat_labels
groundtruth$ADT_Umap = data_obj$adt_umap_labels
groundtruth$ADT_pca = data_obj$adt_pca_labels

groundtruth$consensus = 0
groundtruth[groundtruth$RCA == 1,]$consensus = "T1"
groundtruth[groundtruth$ADT_pca==0 & groundtruth$RCA == 1,]$consensus = "CD4 T Naive"
groundtruth[groundtruth$ADT_pca==3 & groundtruth$RCA == 1,]$consensus = "CD4 T Memory"
groundtruth[groundtruth$ADT_pca==6 & groundtruth$RCA == 1,]$consensus = "CD8 T"

groundtruth[groundtruth$RCA == 2,]$consensus = "Mono"
groundtruth[groundtruth$ADT_pca==1 & groundtruth$RCA == 2,]$consensus = "Mono"
groundtruth[groundtruth$ADT_pca==8 & groundtruth$RCA == 2,]$consensus = "CD16 Mono"
groundtruth[groundtruth$ADT_pca==9 & groundtruth$RCA == 2,]$consensus = "CD14 Mono"

groundtruth[groundtruth$RCA == 3,]$consensus = "NK"
groundtruth[groundtruth$ADT_Umap==2 & groundtruth$RCA == 3,]$consensus = "NK"
groundtruth[groundtruth$ADT_Umap==11 & groundtruth$RCA == 3,]$consensus = "NK T"

groundtruth[groundtruth$RCA == 4,]$consensus = "B"
groundtruth[groundtruth$ADT_pca==7 & groundtruth$RCA == 4,]$consensus = "B"
groundtruth[groundtruth$ADT_pca==11 & groundtruth$RCA == 4,]$consensus = "CD3+ B"

groundtruth[groundtruth$RCA == 5 | groundtruth$RCA == 15,]$consensus = "CD16 Mono"
groundtruth[groundtruth$ADT_pca==8 & groundtruth$consensus == "CD16 Mono",]$consensus =
"CD16 Mono"
groundtruth[groundtruth$ADT_pca==4 & groundtruth$consensus == "CD16 Mono",]$consensus =
"Myeloid-like NK"

groundtruth[groundtruth$RCA == 6,]$consensus = "Progenitor"

groundtruth[groundtruth$RCA == 7,]$consensus = "mDC"

groundtruth[groundtruth$RCA == 8,]$consensus = "pDC"

groundtruth[groundtruth$RCA == 9,]$consensus = "T2"

groundtruth[groundtruth$RCA == 10,]$consensus = "Neutrophils"

groundtruth[groundtruth$RCA == 11 | groundtruth$RCA == 12,]$consensus = "Myeloid-like NK"
```

```
groundtruth[groundtruth$RCA == 13,]$consensus = "Progenitor_B"

groundtruth[groundtruth$RCA == 14,]$consensus = "CD3+ B"

dim(groundtruth[groundtruth$consensus==0,])

sort(table(groundtruth$consensus))

groundtruthDataList = list()
groundtruthDataList[["CBMC"]] = data[,rownames(groundtruth[groundtruth$consensus!=0,])]
groundtruthGroupList = list()
groundtruthGroupList[["CBMC"]] = groundtruth[groundtruth$consensus!=0,]$consensus

save(groundtruthDataList,groundtruthGroupList,file="groundtruth_CBMC_Monaco.RData")

##################################################PBMC
Dropseq#############################################################
# load the original data
data_original = read.csv("../feature_selection/raw_datasets/GSE100866_PBMC_vs_flow_10X-
RNA_umi.csv.gz",sep=",",header=T,row.names=1)
pbmc.rna.collapsed <- CollapseSpeciesExpressionMatrix(data_original)
pbmc <- CreateSeuratObject(pbmc.rna.collapsed,min.cells = 100, min.features = 500, project = "pbmc")
mito.features <- grep(pattern = "^MT-", x = rownames(x = pbmc), value = TRUE)
percent.mito <- Matrix::colSums(x = GetAssayData(object = pbmc, slot = 'counts')[mito.features, ]) /
Matrix::colSums(x = GetAssayData(object = pbmc, slot = 'counts'))
pbmc[['percent.mito']] <- percent.mito
VlnPlot(object = pbmc, features = c("nFeature_RNA", "nCount_RNA", "percent.mito"), ncol = 3)
pbmc <- subset(x = pbmc, subset = nFeature_RNA > 500 & nFeature_RNA < 2000 & percent.mito < 0.08)
pbmc <- NormalizeData(pbmc)
pbmc <- FindVariableFeatures(pbmc,selection.method = "vst",nfeatures = 1000)
pbmc <- ScaleData(pbmc, display.progress = FALSE)
pbmc <- RunPCA(pbmc,features = VariableFeatures(object = pbmc))
ElbowPlot(pbmc)
pbmc <- FindNeighbors(object = pbmc, reduction = "pca", dims = 1:15)
pbmc <- FindClusters(pbmc, resolution = 0.2)
pbmc <- RunTSNE(pbmc, dims = 1:15)

pbmc.markers <- FindAllMarkers(object = pbmc, only.pos = TRUE, min.pct = 0.25, logfc.threshold = 0.25)
pbmc.markers %>% group_by(cluster) %>% top_n(n = 5, wt = avg_logFC) %>% print(n=26)

new.cluster.ids <- c("CD4 T", "CD14+ Mono", "NK", "B","DC", "CD8 T","FCGR3A+ Mono", "Mouse")
names(x = new.cluster.ids) <- levels(x = pbmc)
pbmc <- RenameIdents(object = pbmc, new.cluster.ids)
DimPlot(object = pbmc, reduction = 'tsne', label = TRUE, pt.size = 0.5)
table(pbmc@active.ident)
pbmc[["rna_8clusters"]] <- Idents(object = pbmc)

# load the ADT UMI data
```

```
pbmc.adt <- read.csv("/mnt/volume1/project/pbmc_10x_8k/GSE100866_PBMC_vs_flow_10X-
ADT_umi.csv",sep = ",", header = TRUE, row.names = 1)
pbmc.adt = pbmc.adt[,names(pbmc@active.ident)]
pbmc.cite <- CreateSeuratObject(counts = pbmc.adt)
pbmc.cite = NormalizeData(object = pbmc.cite, method = "CLR")
pbmc.cite <- ScaleData(pbmc.cite, features = NULL, display.progress = FALSE)

###### cluster based on ADT data
# Cluster based on PCA
pbmc.cite <- RunPCA(pbmc.cite, features = rownames(pbmc.cite@assays$RNA))
pbmc.cite <- FindNeighbors(object = pbmc.cite, reduction = "pca", dims = 1:9)
pbmc.cite <- FindClusters(pbmc.cite, resolution = 0.5, Reduction.type = "pca")
pbmc.cite[["rna_8clusters"]] <- Idents(object = pbmc)
adt_pca_labels = pbmc.cite@active.ident
pbmc.cite <- RunTSNE(pbmc.cite, dims = 1:9)
DimPlot(object = pbmc.cite, reduction = 'tsne', label = TRUE, pt.size = 0.5)
pbmc.small <- SubsetData(pbmc.cite, max.cells.per.ident = 300)
adt.markers <- FindAllMarkers(pbmc.small, only.pos = TRUE)
DoHeatmap(pbmc.small, features = unique(adt.markers$gene),size=3)
DoHeatmap(pbmc.small, features = unique(adt.markers$gene),size=3,group.by = "rna_8clusters")

# Cluser based on UMAP
pbmc.cite <- RunUMAP(pbmc.cite,features = rownames(pbmc.cite@assays$RNA))
pbmc.cite <- FindNeighbors(object = pbmc.cite, reduction = "umap", dims = 1:2)
pbmc.cite <- FindClusters(pbmc.cite, resolution = 0.16, Reduction.type = "umap")
DimPlot(object = pbmc.cite, reduction = 'umap', label = TRUE, pt.size = 0.5)
pbmc.small <- SubsetData(pbmc.cite, max.cells.per.ident = 300)
adt.markers <- FindAllMarkers(pbmc.small, only.pos = TRUE)
DoHeatmap(pbmc.small, features = unique(adt.markers$gene),size=3)

# load data after filtering
data=benchmarkDataList$`PBMC 10X` # in normal domain after filtering

# Intersection of data and the original data
data = data[, colnames(data) %in% colnames(pbmc@assays$RNA@data)]

# Run RCA global panel to label cells
data_obj = dataConstruct(as.matrix(data))
data_obj = geneFilt(obj_in = data_obj)
data_obj = cellNormalize(data_obj)
data_obj = dataTransform(data_obj)
data_obj = featureConstruct(data_obj,method = "GlobalPanel",power = 4)

data_obj = cellClust(data_obj,deepSplit_wgcna=0)
RCAPlot(data_obj)

# Run RCA Monaco panel to label cells
data_obj = dataConstruct(as.matrix(data))
```

```
data_obj = geneFilt(obj_in = data_obj)
data_obj = cellNormalize(data_obj)
data_obj = dataTransform(data_obj)
source('/mnt/volume1/project/code/featureConstructMonaco.R')
data_obj = featureConstructMonaco(data_obj,method = "MonacoPanel",power = 4)

data_obj = cellClust(data_obj,deepSplit_wgcna=3)
RCAPlot(data_obj)

#get the seurat label for filtered data
data_obj$seurat_labels = pbmc@active.ident[colnames(data)]
data_obj$adt_umap_labels = pbmc.cite@active.ident[colnames(data)]
data_obj$adt_pca_labels = adt_pca_labels[colnames(data)]

#Umap using RCA global panel clustering
seed = 42
seurat_obj = CreateSeuratObject(data_obj$fpkm_for_clust)
seurat_obj <- ScaleData(object = seurat_obj, display.progress = FALSE)

seurat_obj = RunUMAP(obj=seurat_obj,features = rownames(seurat_obj@assays$RNA@data))
seurat_obj@active.ident = as.factor(data_obj$group_labels_color$groupLabel)
names(seurat_obj@active.ident) = colnames(data)

colors = as.character(unique(data_obj$group_labels_color$dynamicColors))
labels = unique(data_obj$group_labels_color$groupLabel)
colors_sorted = colors[sort.int(labels,index.return = T)$ix]
DimPlot(object = seurat_obj, reduction = 'umap',group.by = "ident",cols = colors_sorted)

# label using seurat labels
seurat_obj@active.ident = as.factor(data_obj$seurat_labels)
names(seurat_obj@active.ident) = colnames(data)

colors = unique(labels2colors(data_obj$seurat_labels))
DimPlot(object = seurat_obj, reduction = 'umap',group.by = "ident",cols = colors)

# label using ADT labels
seurat_obj@active.ident = as.factor(data_obj$adt_pca_labels)
names(seurat_obj@active.ident) = colnames(data)

colors = unique(labels2colors(data_obj$adt_pca_labels))
DimPlot(object = seurat_obj, reduction = 'umap',group.by = "ident",cols = colors)

source('/mnt/volume1/project/plotHeatmap.R')
cellTypeDEPlot(data_obj$fpkm_for_clust,cellTree = data_obj$cellTree,clusterLabels =
data_obj$group_labels_color$groupLabel,dynamicColors = data_obj$dynamicColors,filename =
"heatmap_RCA_Monacopanel_deepsplit3")

# Find the consensus groundtruth based on RCA and Seurat clustering
```

```
groundtruth = as.data.frame(data_obj$group_labels_color$groupLabel)
rownames(groundtruth) = colnames(data)
colnames(groundtruth) = "RCA"
groundtruth$RNA = data_obj$seurat_labels
groundtruth$ADT_Umap = data_obj$adt_umap_labels
groundtruth$ADT_pca = data_obj$adt_pca_labels

groundtruth$consensus = 0
groundtruth[groundtruth$RCA == 1,]$consensus = "T"
groundtruth[groundtruth$ADT_pca==0 & groundtruth$RCA == 1,]$consensus = "CD4 T Memory"
groundtruth[groundtruth$ADT_pca==1 & groundtruth$RCA == 1,]$consensus = "CD4 T Naive"
groundtruth[groundtruth$ADT_pca==5 & groundtruth$RCA == 1,]$consensus = "CD8 T Naive"
groundtruth[groundtruth$ADT_pca==6 & groundtruth$RCA == 1,]$consensus = "CD8 T Memory"
groundtruth[groundtruth$ADT_pca==8 & groundtruth$RCA == 1,]$consensus = "NK T"

groundtruth[groundtruth$RCA == 2,]$consensus = "Mono"
groundtruth[groundtruth$ADT_pca==3 & groundtruth$RCA == 2,]$consensus = "Mono"
groundtruth[groundtruth$ADT_pca==7 & groundtruth$RCA == 2,]$consensus = "CD14 Mono"
groundtruth[groundtruth$ADT_pca==9 & groundtruth$RCA == 2,]$consensus = "CD14 Mono"

groundtruth[groundtruth$RCA == 3,]$consensus = "NK"
groundtruth[groundtruth$ADT_pca==2 & groundtruth$RCA == 3,]$consensus = "NK"
groundtruth[groundtruth$ADT_pca==5 & groundtruth$RCA == 3,]$consensus = "CD8 T Naive"
groundtruth[groundtruth$ADT_pca==6 & groundtruth$RCA == 3,]$consensus = "CD8 T Memory"
groundtruth[groundtruth$ADT_pca==8 & groundtruth$RCA == 3,]$consensus = "NK T"

groundtruth[groundtruth$RCA == 4,]$consensus = "B"
groundtruth[groundtruth$ADT_pca==4 & groundtruth$RCA == 4,]$consensus = "B"
groundtruth[groundtruth$ADT_pca==11 & groundtruth$RCA == 4,]$consensus = "CD3+ B"

groundtruth[groundtruth$RCA == 5,]$consensus = "DC"

groundtruth[groundtruth$RCA == 6,]$consensus = "Myeloid-like NK"

groundtruth[groundtruth$RCA == 7,]$consensus = "Precursors"

groundtruth[groundtruth$RCA == 8,]$consensus = "Plasma"

dim(groundtruth[groundtruth$consensus==0,])

groundtruthDataList = list()
groundtruthDataList[["pbmc_dropseq"]] = data[,rownames(groundtruth[groundtruth$consensus!=0,])]
groundtruthGroupList = list()
groundtruthGroupList[["pbmc_dropseq"]] = groundtruth[groundtruth$consensus!=0,]$consensus

save(groundtruthDataList,groundtruthGroupList,file="groundtruth_PBMC_dropseq.RData")

# Find the consensus groundtruth based on RCA and Seurat clustering
```

```
groundtruth = as.data.frame(data_obj$group_labels_color$groupLabel)
rownames(groundtruth) = colnames(data)
colnames(groundtruth) = "RCA"
groundtruth$RNA = data_obj$seurat_labels
groundtruth$ADT_Umap = data_obj$adt_umap_labels
groundtruth$ADT_pca = data_obj$adt_pca_labels

groundtruth$consensus = 0
groundtruth[groundtruth$RCA == 1,]$consensus = "T"
groundtruth[groundtruth$ADT_pca==0 & groundtruth$RCA == 1,]$consensus = "CD4 T Memory"
groundtruth[groundtruth$ADT_pca==1 & groundtruth$RCA == 1,]$consensus = "CD4 T Naive"
groundtruth[groundtruth$ADT_pca==5 & groundtruth$RCA == 1,]$consensus = "CD8 T Naive"
groundtruth[groundtruth$ADT_pca==6 & groundtruth$RCA == 1,]$consensus = "CD8 T Memory"
groundtruth[groundtruth$ADT_pca==8 & groundtruth$RCA == 1,]$consensus = "NK T"

groundtruth[groundtruth$RCA == 2,]$consensus = "NK"
groundtruth[groundtruth$ADT_pca==2 & groundtruth$RCA == 2,]$consensus = "NK"
groundtruth[groundtruth$ADT_pca==5 & groundtruth$RCA == 2,]$consensus = "CD8 T Naive"
groundtruth[groundtruth$ADT_pca==6 & groundtruth$RCA == 2,]$consensus = "CD8 T Memory"
groundtruth[groundtruth$ADT_pca==1 & groundtruth$RCA == 2,]$consensus = "CD4 T Naive"
groundtruth[groundtruth$ADT_pca==8 & groundtruth$RCA == 2,]$consensus = "NK T"

groundtruth[groundtruth$RCA == 3 | groundtruth$RCA == 17,]$consensus = "C_Mono"

groundtruth[groundtruth$RCA == 4 | groundtruth$RCA == 26,]$consensus = "CD4 T_1"
groundtruth[groundtruth$ADT_pca==0 & groundtruth$consensus == "CD4 T_1",]$consensus = "CD4 T
Memory"
groundtruth[groundtruth$ADT_pca==1 & groundtruth$consensus == "CD4 T_1",]$consensus = "CD4 T
Naive"
groundtruth[groundtruth$ADT_pca==6 & groundtruth$consensus == "CD4 T_1",]$consensus = "CD8 T
Memory"
groundtruth[groundtruth$ADT_pca==8 & groundtruth$consensus == "CD4 T_1",]$consensus = "NK T"

groundtruth[groundtruth$RCA == 5 | groundtruth$RCA == 25 | groundtruth$RCA == 28,]$consensus =
"B"
groundtruth[groundtruth$ADT_pca==11 & groundtruth$consensus == "B",]$consensus = "CD3+ B"

groundtruth[groundtruth$RCA == 6 | groundtruth$RCA == 27,]$consensus = "I_Mono"

groundtruth[groundtruth$RCA == 7,]$consensus = "CD8 T_1"
groundtruth[groundtruth$ADT_pca==6 & groundtruth$consensus == "CD8 T_1",]$consensus = "CD8 T
Memory"
groundtruth[groundtruth$ADT_pca==8 & groundtruth$consensus == "CD8 T_1",]$consensus = "NK T"
groundtruth[groundtruth$ADT_pca==5 & groundtruth$consensus == "CD8 T_1",]$consensus = "CD8 T
Naive"

groundtruth[groundtruth$RCA == 8 | groundtruth$RCA == 22,]$consensus = "CD4 T_2"
```

```
groundtruth[groundtruth$ADT_pca==0 & groundtruth$consensus == "CD4 T_2",]$consensus = "CD4 T Memory"
groundtruth[groundtruth$ADT_pca==6 & groundtruth$consensus == "CD4 T_2",]$consensus = "CD8 T Memory"
groundtruth[groundtruth$ADT_pca==8 & groundtruth$consensus == "CD4 T_2",]$consensus = "NK T"
groundtruth[groundtruth$ADT_pca==5 & groundtruth$consensus == "CD4 T_2",]$consensus = "CD8 T Naive"
groundtruth[groundtruth$ADT_pca==1 & groundtruth$consensus == "CD4 T_2",]$consensus = "CD4 T Naive"

groundtruth[groundtruth$RCA == 9,]$consensus = "CD8 T Naive"
groundtruth[groundtruth$ADT_pca==1 & groundtruth$consensus == "CD8 T Naive",]$consensus = "CD4 T Naive"
groundtruth[groundtruth$ADT_pca==6 & groundtruth$consensus == "CD8 T Naive",]$consensus = "CD8 T Memory"

groundtruth[groundtruth$RCA == 10,]$consensus = "MAIT"

groundtruth[groundtruth$RCA == 11 | groundtruth$RCA ==15,]$consensus = "CD8 T_2"
groundtruth[groundtruth$ADT_pca==6 & groundtruth$consensus == "CD8 T_2",]$consensus = "CD8 T Memory"
groundtruth[groundtruth$ADT_pca==5 & groundtruth$consensus == "CD8 T_2",]$consensus = "CD8 T Naive"
groundtruth[groundtruth$ADT_pca==8 & groundtruth$consensus == "CD8 T_2",]$consensus = "NK T"
groundtruth[groundtruth$ADT_pca==0 & groundtruth$consensus == "CD8 T_2",]$consensus = "CD4 T Memory"

groundtruth[groundtruth$RCA == 12 | groundtruth$RCA ==21,]$consensus = "mDC"

groundtruth[groundtruth$RCA == 13,]$consensus = "CD8 T_3"
groundtruth[groundtruth$ADT_pca==8 & groundtruth$consensus == "CD8 T_3",]$consensus = "NK T"
groundtruth[groundtruth$ADT_pca==1 & groundtruth$consensus == "CD8 T_3",]$consensus = "CD4 T Naive"
groundtruth[groundtruth$ADT_pca==0 & groundtruth$consensus == "CD8 T_3",]$consensus = "CD4 T Memory"
groundtruth[groundtruth$ADT_pca==5 & groundtruth$consensus == "CD8 T_3",]$consensus = "CD8 T Naive"
groundtruth[groundtruth$ADT_pca==6 & groundtruth$consensus == "CD8 T_3",]$consensus = "CD8 T Memory"

groundtruth[groundtruth$RCA == 14 | groundtruth$RCA == 23,]$consensus = "CD8 T_4"
groundtruth[groundtruth$ADT_pca==5 & groundtruth$consensus == "CD8 T_4",]$consensus = "CD8 T Naive"
groundtruth[groundtruth$ADT_pca==6 & groundtruth$consensus == "CD8 T_4",]$consensus = "CD8 T Memory"
groundtruth[groundtruth$ADT_pca==8 & groundtruth$consensus == "CD8 T_4",]$consensus = "NK T"
groundtruth[groundtruth$ADT_pca==1 & groundtruth$consensus == "CD8 T_4",]$consensus = "CD4 T Naive"
```

```
groundtruth[groundtruth$ADT_pca==0 & groundtruth$consensus == "CD8 T_4",]$consensus = "CD4 T
Memory"

groundtruth[groundtruth$RCA == 16,]$consensus = "pDC"

groundtruth[groundtruth$RCA == 18,]$consensus = "Neutrophils"

groundtruth[groundtruth$RCA == 19,]$consensus = "Myeloid-like NK"

groundtruth[groundtruth$RCA == 20,]$consensus = "Progenitor"

groundtruth[groundtruth$RCA == 24,]$consensus = "Plasma"

groundtruth[groundtruth$consensus == "CD8 T_3" | groundtruth$consensus == "T" |
groundtruth$consensus == "CD4 T_1"|
        groundtruth$consensus == "CD4 T_2" | groundtruth$consensus == "CD8 T_1",]$consensus =
"T"

dim(groundtruth[groundtruth$consensus==0,])

sort(table(groundtruth$consensus))

groundtruthDataList = list()
groundtruthDataList[["pbmc_dropseq"]] = data[,rownames(groundtruth[groundtruth$consensus!=0,])]
groundtruthGroupList = list()
groundtruthGroupList[["pbmc_dropseq"]] = groundtruth[groundtruth$consensus!=0,]$consensus

save(groundtruthDataList,groundtruthGroupList,file="groundtruth_PBMC_dropseq_Monaco.RData")

######################MALT#################################################
# load the original data
data_original = readMM("/mnt/volume1/project/malt_10k/matrix.mtx.gz")
data_genes =
read.csv("/mnt/volume1/project/malt_10k/features.tsv.gz",header=F,sep="\t",stringsAsFactors = F)
data_cells =
read.csv("/mnt/volume1/project/malt_10k/barcodes.tsv.gz",header=F,sep="\t",stringsAsFactors = F)

data_original@Dimnames[[1]] = data_genes$V2
data_original@Dimnames[[2]] = data_cells$V1

malt.rna.collapsed <- CollapseSpeciesExpressionMatrix(data_original[1:33538,])
malt <- CreateSeuratObject(malt.rna.collapsed,min.cells = 100, min.features = 500, project = "malt")
mito.features <- grep(pattern = "^MT-", x = rownames(x = malt), value = TRUE)
percent.mito <- Matrix::colSums(x = GetAssayData(object = malt, slot = 'counts')[mito.features, ]) /
Matrix::colSums(x = GetAssayData(object = malt, slot = 'counts'))
malt[['percent.mito']] <- percent.mito
VlnPlot(object = malt, features = c("nFeature_RNA", "nCount_RNA", "percent.mito"), ncol = 3)
malt <- subset(x = malt, subset = nFeature_RNA > 500 & nFeature_RNA < 3000 & percent.mito < 0.3)
```

```
malt <- NormalizeData(malt)
malt <- FindVariableFeatures(malt,selection.method = "vst",nfeatures = 1000)
malt <- ScaleData(malt, display.progress = FALSE)
malt <- RunPCA(malt,features = VariableFeatures(object = malt))
ElbowPlot(malt)
malt <- FindNeighbors(object = malt, reduction = "pca", dims = 1:15)
malt <- FindClusters(malt, resolution = 0.2)
malt <- RunTSNE(malt, dims = 1:15)

malt.markers <- FindAllMarkers(object = malt, only.pos = TRUE, min.pct = 0.25, logfc.threshold = 0.25)
malt.markers %>% group_by(cluster) %>% top_n(n = 5, wt = avg_logFC) %>% print(n=50)
filter(malt.markers, cluster == "0") %>% top_n(n=20,wt=avg_logFC)

new.cluster.ids <- c("B", "Follicular B", "CD4 T", "CD8 T","TIGIT+ T", "B1","T", "Plasma","Macro")
names(x = new.cluster.ids) <- levels(x = malt)
malt <- RenameIdents(object = malt, new.cluster.ids)
DimPlot(object = malt, reduction = 'tsne', label = TRUE, pt.size = 0.5,label.size = 8)+NoLegend()
table(malt@active.ident)
malt[["rna_9clusters"]] <- Idents(object = malt)

# load the ADT UMI data
malt.adt <- as.matrix(data_original[33539:33554,])
malt.adt = malt.adt[,names(malt@active.ident)]
malt.cite <- CreateSeuratObject(counts = malt.adt)
malt.cite = NormalizeData(object = malt.cite, method = "CLR")
malt.cite <- ScaleData(malt.cite, features = NULL, display.progress = FALSE)

###### cluster based on ADT data
# Cluster based on PCA
malt.cite <- RunPCA(malt.cite, features = rownames(malt.cite@assays$RNA))
malt.cite <- FindNeighbors(object = malt.cite, reduction = "pca", dims = 1:15)
malt.cite <- FindClusters(malt.cite, resolution = 0.65, Reduction.type = "pca")
malt.cite[["rna_9clusters"]] <- Idents(object = malt)
adt_pca_labels = malt.cite@active.ident
malt.cite <- RunTSNE(malt.cite, dims = 1:15)
DimPlot(object = malt.cite, reduction = 'tsne', label = TRUE, pt.size = 0.5)
malt.small <- SubsetData(malt.cite, max.cells.per.ident = 300)
adt.markers <- FindAllMarkers(malt.small, only.pos = TRUE)
DoHeatmap(malt.small, features = unique(adt.markers$gene),size=3)
DoHeatmap(malt.small, features = unique(adt.markers$gene),size=3,group.by = "rna_9clusters")

# Cluser based on UMAP
malt.cite <- RunUMAP(malt.cite,features = rownames(malt.cite@assays$RNA))
malt.cite <- FindNeighbors(object = malt.cite, reduction = "umap", dims = 1:2)
malt.cite <- FindClusters(malt.cite, resolution = 0.14, Reduction.type = "umap")
DimPlot(object = malt.cite, reduction = 'umap', label = TRUE, pt.size = 0.5)
malt.small <- SubsetData(malt.cite, max.cells.per.ident = 300)
adt.markers <- FindAllMarkers(malt.small, only.pos = TRUE)
```

```
DoHeatmap(malt.small, features = unique(adt.markers$gene),size=3)

# load data after filtering
data=benchmarkDataList$MALT # in normal domain after filtering
colnames(data) <- paste0(colnames(data),"-1")

# Intersection of data and the original data
data = data[, colnames(data) %in% colnames(malt@assays$RNA@data)]

# Run RCA global panel to label cells
data_obj = dataConstruct(as.matrix(data))
data_obj = geneFilt(obj_in = data_obj)
data_obj = cellNormalize(data_obj)
data_obj = dataTransform(data_obj)
data_obj = featureConstruct(data_obj,method = "GlobalPanel",power = 4)

data_obj = cellClust(data_obj,deepSplit_wgcna=0)
RCAPlot(data_obj)

# Run RCA Monaco panel to label cells
data_obj = dataConstruct(as.matrix(data))
data_obj = geneFilt(obj_in = data_obj)
data_obj = cellNormalize(data_obj)
data_obj = dataTransform(data_obj)
source('/mnt/volume1/project/code/featureConstructMonaco.R')
data_obj = featureConstructMonaco(data_obj,method = "MonacoPanel",power = 4)

data_obj = cellClust(data_obj,deepSplit_wgcna=3)
RCAPlot(data_obj)
source('/mnt/volume1/project/plotHeatmap.R')
cellTypeDEPlot(data_obj$fpkm_for_clust,cellTree = data_obj$cellTree,clusterLabels =
data_obj$group_labels_color$groupLabel,dynamicColors = data_obj$dynamicColors,filename =
"heatmap_RCA_Monacopanel_deepsplit3")

# cluster based on ADT data and label using RCA Monaco panel
malt.adt <- as.matrix(data_original[33539:33554,])

# Intersection of data and the original data
malt.adt = malt.adt[, colnames(malt.adt) %in% colnames(data)]

malt.cite <- CreateSeuratObject(counts = malt.adt)
malt.cite = NormalizeData(object = malt.cite, method = "CLR")
malt.cite <- ScaleData(malt.cite, features = NULL, display.progress = FALSE)

###### cluster based on ADT data
# Cluster based on PCA
malt.cite <- RunPCA(malt.cite, features = rownames(malt.cite@assays$RNA))
malt.cite <- FindNeighbors(object = malt.cite, reduction = "pca", dims = 1:15)
```

```
malt.cite <- FindClusters(malt.cite, resolution = 0.65, Reduction.type = "pca")
malt.cite[["rca_monaco"]] <- as.factor(data_obj$group_labels_color$groupLabel)
adt_pca_labels = malt.cite@active.ident
malt.cite <- RunTSNE(malt.cite, dims = 1:15)
DimPlot(object = malt.cite, reduction = 'tsne', label = TRUE, pt.size = 0.5)
malt.small <- SubsetData(malt.cite, max.cells.per.ident = 300)
adt.markers <- FindAllMarkers(malt.small, only.pos = TRUE)
DoHeatmap(malt.small, features = unique(adt.markers$gene),size=3)
DoHeatmap(malt.small, features = unique(adt.markers$gene),size=3,group.by = "rca_monaco")


#get the seurat label for filtered data
data_obj$seurat_labels = malt@active.ident[colnames(data)]
data_obj$adt_umap_labels = malt.cite@active.ident[colnames(data)]
data_obj$adt_pca_labels = adt_pca_labels[colnames(data)]

#Umap using RCA global panel clustering
seed = 42
seurat_obj = CreateSeuratObject(data_obj$fpkm_for_clust)
seurat_obj <- ScaleData(object = seurat_obj, display.progress = FALSE)

seurat_obj = RunUMAP(obj=seurat_obj,features = rownames(seurat_obj@assays$RNA@data))
seurat_obj@active.ident = as.factor(data_obj$group_labels_color$groupLabel)
names(seurat_obj@active.ident) = colnames(data)

colors = as.character(unique(data_obj$group_labels_color$dynamicColors))
labels = unique(data_obj$group_labels_color$groupLabel)
colors_sorted = colors[sort.int(labels,index.return = T)$ix]
DimPlot(object = seurat_obj, reduction = 'umap',group.by = "ident",cols = colors_sorted)

# label using seurat labels
seurat_obj@active.ident = as.factor(data_obj$seurat_labels)
names(seurat_obj@active.ident) = colnames(data)

colors = unique(labels2colors(data_obj$seurat_labels))
colors[6] = "pink"
colors[8] = "blue"
DimPlot(object = seurat_obj, reduction = 'umap',group.by = "ident",cols = colors)

# label using ADT labels
seurat_obj@active.ident = as.factor(data_obj$adt_pca_labels)
names(seurat_obj@active.ident) = colnames(data)

# highlight the marker genes for plasma cells
cells_seurat = colnames(data_obj$fpkm_for_clust[data_obj$seurat_labels == "Plasma"])
cells_rca = colnames(data_obj$fpkm_for_clust[which(data_obj$group_labels_color$groupLabel == 10 |
data_obj$group_labels_color$groupLabel == 12)])
```

```
FeaturePlot(malt,cols = c("lightgrey","darkblue"), features = c("CD27"), cells = cells_seurat)
FeaturePlot(malt,cols = c("lightgrey","darkblue"), features = c("CD27"), cells = cells_rca)

FeaturePlot(malt,cols = c("lightgrey","darkblue"), features = c("PRDM1"), cells = cells_seurat)
FeaturePlot(malt,cols = c("lightgrey","darkblue"), features = c("PRDM1"), cells = cells_rca)

FeaturePlot(malt,cols = c("lightgrey","darkblue"), features = c("DERL3"), cells = cells_seurat)
FeaturePlot(malt,cols = c("lightgrey","darkblue"), features = c("DERL3"), cells = cells_rca)

library(ggplot2)
colors =
c("lightgrey","lightgrey","lightgrey","lightgrey","lightgrey","lightgrey","lightgrey","lightgrey","lightgrey")
plot<-DimPlot(object = seurat_obj, reduction = 'umap',group.by = "ident", cols = colors, do.return=T)
# plot <- plot+geom_point(data=as.data.frame(data=malt@assays$RNA@counts["CD27",cells_seurat]),
# mapping = aes(x = seurat_obj@reductions$umap[[,1]], y =
seurat_obj@reductions$umap[[,2]],color=))+scale_color_gradient(low="lightgrey", high="darkblue")

data_df <- as.data.frame(seurat_obj@reductions$umap@cell.embeddings)

data_df <- cbind(data_df, data.frame(cd27 =
malt@assays$RNA@scale.data["CD27",rownames(data_df)]))
ggplot(data=data_df,
    mapping = aes(x = UMAP_1, y = UMAP_2, color=cd27))+scale_color_gradient(low="lightgrey",
high="darkblue") + geom_point()

data_df <- cbind(data_df, data.frame(PRDM1 =
malt@assays$RNA@scale.data["PRDM1",rownames(data_df)]))
ggplot(data=data_df,
    mapping = aes(x = UMAP_1, y = UMAP_2, color=PRDM1))+scale_color_gradient2(low="lightgrey",
mid = "darkblue", high="black",midpoint = 3) + geom_point()

data_df <- cbind(data_df, data.frame(DERL3 =
malt@assays$RNA@scale.data["DERL3",rownames(data_df)]))
ggplot(data=data_df,
    mapping = aes(x = UMAP_1, y = UMAP_2, color=DERL3))+scale_color_gradient2(low="lightgrey",
mid = "darkblue", high="black",midpoint = 3) + geom_point()

data_df <- cbind(data_df, data.frame(BCL11A =
malt@assays$RNA@scale.data["BCL11A",rownames(data_df)]))
ggplot(data=data_df,
    mapping = aes(x = UMAP_1, y = UMAP_2, color=BCL11A))+scale_color_gradient2(low="lightgrey",
mid = "darkblue", high="black",midpoint = 3) + geom_point()

data_df <- cbind(data_df, data.frame(FCER2 =
malt@assays$RNA@scale.data["FCER2",rownames(data_df)]))
ggplot(data=data_df,
    mapping = aes(x = UMAP_1, y = UMAP_2, color=FCER2))+scale_color_gradient2(low="lightgrey", mid
= "darkblue", high="black",midpoint = 3) + geom_point()
```

```
genes = c("IGHG3", "SSPN", "TNFRSF13B", "SLCO4A1", "RGS16", "IGLC3", "IGHG1", "DERL3", "PNOC")
data_df <- cbind(data_df, data.frame(Markers =
colMeans(malt@assays$RNA@scale.data[genes[which(genes %in%
rownames(malt@assays$RNA@scale.data))] ,rownames(data_df)])))

ggplot(data=data_df,
       mapping = aes(x = UMAP_1, y = UMAP_2, color=Markers))+scale_color_gradient2(low="lightgrey",
mid = "darkblue", high="black",midpoint = 3) + geom_point()

source('/mnt/volume1/project/plotHeatmap.R')
cellTypeDEPlot(data_obj$fpkm_for_clust,cellTree = data_obj$cellTree,clusterLabels =
data_obj$group_labels_color$groupLabel,dynamicColors = data_obj$dynamicColors,filename =
"heatmap_RCA_Monacopanel_deepsplit3")

# Find the consensus groundtruth based on RCA and Seurat clustering
groundtruth = as.data.frame(data_obj$group_labels_color$groupLabel)
rownames(groundtruth) = colnames(data)
colnames(groundtruth) = "RCA"
groundtruth$RNA = data_obj$seurat_labels
groundtruth$ADT_Umap = data_obj$adt_umap_labels
groundtruth$ADT_pca = data_obj$adt_pca_labels

groundtruth$consensus = 0
groundtruth[groundtruth$RCA == 1,]$consensus = "B"
groundtruth[groundtruth$ADT_pca==0 & groundtruth$RCA == 1,]$consensus = "CD14- B"
groundtruth[groundtruth$ADT_pca==1 & groundtruth$RCA == 1,]$consensus = "Activated B"
groundtruth[groundtruth$ADT_pca==3 & groundtruth$RCA == 1,]$consensus = "CD14- activated B"

groundtruth[groundtruth$RCA == 2,]$consensus = "T"
groundtruth[groundtruth$ADT_pca==4 & groundtruth$RCA == 2,]$consensus = "CD127+ Memory CD4
T"
groundtruth[groundtruth$ADT_pca==5 & groundtruth$RCA == 2,]$consensus = "CD8 T Memory"
groundtruth[groundtruth$ADT_pca==6 & groundtruth$RCA == 2,]$consensus = "TIGIT+ CD4 T Memory"
groundtruth[groundtruth$ADT_pca==7 & groundtruth$RCA == 2,]$consensus = "PD1+ CD4 T Memory"
groundtruth[groundtruth$ADT_pca==8 & groundtruth$RCA == 2,]$consensus = "CD4 T Memory"
groundtruth[groundtruth$ADT_pca==9 & groundtruth$RCA == 2,]$consensus = "CD127+ CD8 T"
groundtruth[groundtruth$ADT_pca==10 & groundtruth$RCA == 2,]$consensus = "CD127+ CD4 T"

groundtruth[groundtruth$RCA == 3,]$consensus = "Lymphnode"
groundtruth[groundtruth$ADT_pca==6 & groundtruth$RCA == 3,]$consensus = "TIGIT+ CD4 T Memory"

groundtruth[groundtruth$RCA == 4,]$consensus = "Lymphoma B"

groundtruth[groundtruth$RCA == 5,]$consensus = "Plasma"

groundtruth[groundtruth$RCA == 6,]$consensus = "Macro"
```

```
groundtruth[groundtruth$RCA == 7,]$consensus = "DC"

groundtruth[groundtruth$RCA == 8,]$consensus = "NK"

groundtruth[groundtruth$RCA == 9,]$consensus = "Thymus"

dim(groundtruth[groundtruth$consensus==0,])

table(groundtruth$consensus)

groundtruthDataList = list()
groundtruthDataList[["malt"]] = data[,rownames(groundtruth[groundtruth$consensus!=0,])]
groundtruthGroupList = list()
groundtruthGroupList[["malt"]] = groundtruth[groundtruth$consensus!=0,]$consensus

save(groundtruthDataList,groundtruthGroupList,file="groundtruth_malt.RData")


# Find the consensus groundtruth based on RCA and Seurat clustering
groundtruth = as.data.frame(data_obj$group_labels_color$groupLabel)
rownames(groundtruth) = colnames(data)
colnames(groundtruth) = "RCA"
groundtruth$RNA = data_obj$seurat_labels
groundtruth$ADT_Umap = data_obj$adt_umap_labels
groundtruth$ADT_pca = data_obj$adt_pca_labels

groundtruth$consensus = 0
groundtruth[groundtruth$RCA == 1,]$consensus = "B1"
groundtruth[groundtruth$ADT_pca==0 & groundtruth$consensus == "B1",]$consensus = "B"
groundtruth[groundtruth$ADT_pca==1 & groundtruth$consensus == "B1",]$consensus = "Activated B"
groundtruth[groundtruth$ADT_pca==2 & groundtruth$consensus == "B1",]$consensus = "CD14-
activated B"
groundtruth[groundtruth$ADT_pca==3 & groundtruth$consensus == "B1",]$consensus = "CD14- B"

groundtruth[groundtruth$RCA == 2,]$consensus = "B2"
groundtruth[groundtruth$ADT_pca==0 & groundtruth$consensus == "B2",]$consensus = "B"
groundtruth[groundtruth$ADT_pca==1 & groundtruth$consensus == "B2",]$consensus = "Activated B"
groundtruth[groundtruth$ADT_pca==2 & groundtruth$consensus == "B2",]$consensus = "CD14-
activated B"
groundtruth[groundtruth$ADT_pca==3 & groundtruth$consensus == "B2",]$consensus = "CD14- B"

groundtruth[groundtruth$RCA == 3 | groundtruth$RCA == 22,]$consensus = "T1"
groundtruth[groundtruth$ADT_pca==4 & groundtruth$consensus == "T1",]$consensus = "CD127+
Memory CD4 T"
groundtruth[groundtruth$ADT_pca==5 & groundtruth$consensus == "T1",]$consensus = "TIGIT+ CD4 T
Memory"
groundtruth[groundtruth$ADT_pca==6 & groundtruth$consensus == "T1",]$consensus = "CD8 T
Memory"
```

```
groundtruth[groundtruth$ADT_pca==7 & groundtruth$consensus == "T1",]$consensus = "PD1+ CD4 T
Memory"
groundtruth[groundtruth$ADT_pca==8 & groundtruth$consensus == "T1",]$consensus = "CD127+ CD8
T"

########################## stop here, do not do Monaco panel for MALT

groundtruth[groundtruth$RCA == 4,]$consensus = "Lymphoma B"

groundtruth[groundtruth$RCA == 5,]$consensus = "Plasma"

groundtruth[groundtruth$RCA == 6,]$consensus = "Macro"

groundtruth[groundtruth$RCA == 7,]$consensus = "DC"

groundtruth[groundtruth$RCA == 8,]$consensus = "NK"

groundtruth[groundtruth$RCA == 9,]$consensus = "Thymus"

dim(groundtruth[groundtruth$consensus==0,])

table(groundtruth$consensus)

groundtruthDataList = list()
groundtruthDataList[["malt"]] = data[,rownames(groundtruth[groundtruth$consensus!=0,])]
groundtruthGroupList = list()
groundtruthGroupList[["malt"]] = groundtruth[groundtruth$consensus!=0,]$consensus

save(groundtruthDataList,groundtruthGroupList,file="groundtruth_malt.RData")

###############################PBMC###############################################
# load the original data
data_original = readMM("/mnt/volume1/project/pbmc_10k/matrix.mtx.gz")
data_genes =
read.csv("/mnt/volume1/project/pbmc_10k/features.tsv.gz",header=F,sep="\t",stringsAsFactors = F)
data_cells =
read.csv("/mnt/volume1/project/pbmc_10k/barcodes.tsv.gz",header=F,sep="\t",stringsAsFactors = F)

data_original@Dimnames[[1]] = data_genes$V2
data_original@Dimnames[[2]] = data_cells$V1

pbmc.rna.collapsed <- CollapseSpeciesExpressionMatrix(data_original[1:33538,])
pbmc <- CreateSeuratObject(pbmc.rna.collapsed,min.cells = 100, min.features = 500, project = "pbmc")
mito.features <- grep(pattern = "^MT-", x = rownames(x = pbmc), value = TRUE)
percent.mito <- Matrix::colSums(x = GetAssayData(object = pbmc, slot = 'counts')[mito.features, ]) /
Matrix::colSums(x = GetAssayData(object = pbmc, slot = 'counts'))
pbmc[['percent.mito']] <- percent.mito
VlnPlot(object = pbmc, features = c("nFeature_RNA", "nCount_RNA", "percent.mito"), ncol = 3)
```

```
pbmc <- subset(x = pbmc, subset = nFeature_RNA > 500 & nFeature_RNA < 3000 & percent.mito < 0.15)
pbmc <- NormalizeData(pbmc)
pbmc <- FindVariableFeatures(pbmc,selection.method = "vst",nfeatures = 1000)
pbmc <- ScaleData(pbmc, display.progress = FALSE)
pbmc <- RunPCA(pbmc,features = VariableFeatures(object = pbmc))
ElbowPlot(pbmc)
pbmc <- FindNeighbors(object = pbmc, reduction = "pca", dims = 1:15)
pbmc <- FindClusters(pbmc, resolution = 0.1)
pbmc <- RunTSNE(pbmc, dims = 1:15)

pbmc.markers <- FindAllMarkers(object = pbmc, only.pos = TRUE, min.pct = 0.25, logfc.threshold = 0.25)
pbmc.markers %>% group_by(cluster) %>% top_n(n = 5, wt = avg_logFC) %>% print(n=26)
filter(pbmc.markers, cluster == "0") %>% top_n(n=20,wt=avg_logFC)

new.cluster.ids <- c("CD14 Mono", "CD4 T", "CD4 T","CD8 T", "NK", "B", "Plasma","DC")
names(x = new.cluster.ids) <- levels(x = pbmc)
pbmc <- RenameIdents(object = pbmc, new.cluster.ids)
DimPlot(object = pbmc, reduction = 'tsne', label = TRUE, pt.size = 0.5)
table(pbmc@active.ident)
pbmc[["rna_7clusters"]] <- Idents(object = pbmc)

# load the ADT UMI data
pbmc.adt <- as.matrix(data_original[33539:33555,])
pbmc.adt = pbmc.adt[,names(pbmc@active.ident)]
pbmc.cite <- CreateSeuratObject(counts = pbmc.adt)
pbmc.cite = NormalizeData(object = pbmc.cite, method = "CLR")
pbmc.cite <- ScaleData(pbmc.cite, features = NULL, display.progress = FALSE)

###### cluster based on ADT data
# Cluster based on PCA
seed = 1
pbmc.cite <- RunPCA(pbmc.cite, features = rownames(pbmc.cite@assays$RNA))
pbmc.cite <- FindNeighbors(object = pbmc.cite, reduction = "pca", dims = 1:16)
pbmc.cite <- FindClusters(pbmc.cite, resolution = 0.55, Reduction.type = "pca")
pbmc.cite[["rna_7clusters"]] <- Idents(object = pbmc)
adt_pca_labels = pbmc.cite@active.ident
pbmc.cite <- RunTSNE(pbmc.cite, dims = 1:16, seed.use = seed)
DimPlot(object = pbmc.cite, reduction = 'tsne', label = TRUE, pt.size = 0.5)
pbmc.small <- SubsetData(pbmc.cite, max.cells.per.ident = 300)
adt.markers <- FindAllMarkers(pbmc.small, only.pos = TRUE)
DoHeatmap(pbmc.small, features = unique(adt.markers$gene),size=3)
DoHeatmap(pbmc.small, features = unique(adt.markers$gene),size=3,group.by = "rna_7clusters")

# Cluser based on UMAP
seed = 42
pbmc.cite <- RunUMAP(pbmc.cite,features = rownames(pbmc.cite@assays$RNA))
pbmc.cite <- FindNeighbors(object = pbmc.cite, reduction = "umap", dims = 1:2)
pbmc.cite <- FindClusters(pbmc.cite, resolution = 0.02, Reduction.type = "umap")
```

```
DimPlot(object = pbmc.cite, reduction = 'umap', label = TRUE, pt.size = 0.5)
pbmc.small <- SubsetData(pbmc.cite, max.cells.per.ident = 300)
adt.markers <- FindAllMarkers(pbmc.small, only.pos = TRUE)
DoHeatmap(pbmc.small, features = unique(adt.markers$gene),size=3)

# load data after filtering
data=benchmarkDataList$PBMC # in normal domain after filtering
colnames(data) <- paste0(colnames(data),"-1")

# Intersection of data and the original data
data = data[, colnames(data) %in% colnames(pbmc@assays$RNA@data)]

# Run RCA global panel to label cells
data_obj = dataConstruct(as.matrix(data))
data_obj = geneFilt(obj_in = data_obj)
data_obj = cellNormalize(data_obj)
data_obj = dataTransform(data_obj)
data_obj = featureConstruct(data_obj,method = "GlobalPanel",power = 4)

data_obj = cellClust(data_obj,deepSplit_wgcna=0)
RCAPlot(data_obj)

# Run RCA Monaco panel to label cells
data_obj = dataConstruct(as.matrix(data))
data_obj = geneFilt(obj_in = data_obj)
data_obj = cellNormalize(data_obj)
data_obj = dataTransform(data_obj)
source('/mnt/volume1/project/code/featureConstructMonaco.R')
data_obj = featureConstructMonaco(data_obj,method = "MonacoPanel",power = 4)

data_obj = cellClust(data_obj,deepSplit_wgcna=3)
RCAPlot(data_obj)

#get the seurat label for filtered data
data_obj$seurat_labels = pbmc@active.ident[colnames(data)]
data_obj$adt_umap_labels = pbmc.cite@active.ident[colnames(data)]
data_obj$adt_pca_labels = adt_pca_labels[colnames(data)]

#Umap using RCA global panel clustering
seed = 42
seurat_obj = CreateSeuratObject(data_obj$fpkm_for_clust)
seurat_obj <- ScaleData(object = seurat_obj, display.progress = FALSE)

seurat_obj = RunUMAP(obj=seurat_obj,features = rownames(seurat_obj@assays$RNA@data))
seurat_obj@active.ident = as.factor(data_obj$group_labels_color$groupLabel)
names(seurat_obj@active.ident) = colnames(data)

colors = as.character(unique(data_obj$group_labels_color$dynamicColors))
```

```
labels = unique(data_obj$group_labels_color$groupLabel)
colors_sorted = colors[sort.int(labels,index.return = T)$ix]
DimPlot(object = seurat_obj, reduction = 'umap',group.by = "ident",cols = colors_sorted)

# label using seurat labels
seurat_obj@active.ident = as.factor(data_obj$seurat_labels)
names(seurat_obj@active.ident) = colnames(data)

colors = unique(labels2colors(data_obj$seurat_labels))
DimPlot(object = seurat_obj, reduction = 'umap',group.by = "ident",cols = colors)

# label using ADT labels
seurat_obj@active.ident = as.factor(data_obj$adt_pca_labels)
names(seurat_obj@active.ident) = colnames(data)

colors = unique(labels2colors(data_obj$adt_pca_labels))
DimPlot(object = seurat_obj, reduction = 'umap',group.by = "ident",cols = colors)

source('/mnt/volume1/project/plotHeatmap.R')
cellTypeDEPlot(data_obj$fpkm_for_clust,cellTree = data_obj$cellTree,clusterLabels =
data_obj$group_labels_color$groupLabel,dynamicColors = data_obj$dynamicColors,filename =
"heatmap_RCA_Monacopanel_deepsplit3")

# Find the consensus groundtruth based on RCA and Seurat clustering
groundtruth = as.data.frame(data_obj$group_labels_color$groupLabel)
rownames(groundtruth) = colnames(data)
colnames(groundtruth) = "RCA"
groundtruth$RNA = data_obj$seurat_labels
groundtruth$ADT_Umap = data_obj$adt_umap_labels
groundtruth$ADT_pca = data_obj$adt_pca_labels

groundtruth$consensus = 0
groundtruth[groundtruth$RCA == 1 | groundtruth$RCA == 17 | groundtruth$RCA == 18,]$consensus =
"C_Mono"
groundtruth[groundtruth$ADT_pca==0 & groundtruth$consensus == "C_Mono",]$consensus = "CD14
Mono"
groundtruth[groundtruth$ADT_pca==8 & groundtruth$consensus == "C_Mono",]$consensus = "CD16
Mono"
groundtruth[groundtruth$ADT_pca==10 & groundtruth$consensus == "C_Mono",]$consensus = "CD14
Mono"
groundtruth[groundtruth$ADT_pca==12 & groundtruth$consensus == "C_Mono",]$consensus = "CD16
Mono"

groundtruth[groundtruth$RCA == 2,]$consensus = "T1"
groundtruth[groundtruth$ADT_pca==1 & groundtruth$consensus == "T1",]$consensus = "CD4 T naive"
groundtruth[groundtruth$ADT_pca==3 & groundtruth$consensus == "T1",]$consensus = "CD4 T
Memory"
```

```
groundtruth[groundtruth$ADT_pca==4 & groundtruth$consensus == "T1",]$consensus = "CD8 T
Memory"
groundtruth[groundtruth$ADT_pca==6 & groundtruth$consensus== "T1",]$consensus = "TIGIT+ CD4 T"
groundtruth[groundtruth$ADT_pca==7 & groundtruth$consensus == "T1",]$consensus = "CD8 T Naive"
groundtruth[groundtruth$ADT_pca==9 & groundtruth$consensus == "T1",]$consensus = "TIGIT+ CD8 T"
groundtruth[groundtruth$ADT_pca==11 & groundtruth$consensus == "T1",]$consensus = "CD25+ CD4
T"

groundtruth[groundtruth$RCA == 3,]$consensus = "T2"
groundtruth[groundtruth$ADT_pca==1 & groundtruth$consensus == "T2",]$consensus = "CD4 T naive"
groundtruth[groundtruth$ADT_pca==3 & groundtruth$consensus == "T2",]$consensus = "CD4 T
Memory"
groundtruth[groundtruth$ADT_pca==4 & groundtruth$consensus == "T2",]$consensus = "CD8 T
Memory"
groundtruth[groundtruth$ADT_pca==6 & groundtruth$consensus== "T2",]$consensus = "TIGIT+ CD4 T"
groundtruth[groundtruth$ADT_pca==9 & groundtruth$consensus == "T2",]$consensus = "TIGIT+ CD8 T"
groundtruth[groundtruth$ADT_pca==11 & groundtruth$consensus == "T2",]$consensus = "CD25+ CD4
T"

groundtruth[groundtruth$RCA == 4,]$consensus = "NK"

groundtruth[groundtruth$RCA == 5,]$consensus = "B1"

groundtruth[groundtruth$RCA == 6,]$consensus = "T3"
groundtruth[groundtruth$ADT_pca==1 & groundtruth$consensus == "T3",]$consensus = "CD4 T naive"
groundtruth[groundtruth$ADT_pca==3 & groundtruth$consensus == "T3",]$consensus = "CD4 T
Memory"
groundtruth[groundtruth$ADT_pca==4 & groundtruth$consensus == "T3",]$consensus = "CD8 T
Memory"
groundtruth[groundtruth$ADT_pca==6 & groundtruth$consensus== "T3",]$consensus = "TIGIT+ CD4 T"
groundtruth[groundtruth$ADT_pca==7 & groundtruth$consensus== "T3",]$consensus = "CD8 T Naive"
groundtruth[groundtruth$ADT_pca==9 & groundtruth$consensus == "T3",]$consensus = "TIGIT+ CD8 T"

groundtruth[groundtruth$RCA == 7 | groundtruth$RCA == 19,]$consensus = "MAIT"

groundtruth[groundtruth$RCA == 8,]$consensus = "T4"
groundtruth[groundtruth$ADT_pca==1 & groundtruth$consensus == "T4",]$consensus = "CD4 T naive"
groundtruth[groundtruth$ADT_pca==3 & groundtruth$consensus == "T4",]$consensus = "CD4 T
Memory"
groundtruth[groundtruth$ADT_pca==4 & groundtruth$consensus == "T4",]$consensus = "CD8 T
Memory"
groundtruth[groundtruth$ADT_pca==6 & groundtruth$consensus== "T4",]$consensus = "TIGIT+ CD4 T"
groundtruth[groundtruth$ADT_pca==9 & groundtruth$consensus == "T4",]$consensus = "TIGIT+ CD8 T"
groundtruth[groundtruth$ADT_pca==11 & groundtruth$consensus == "T4",]$consensus = "CD25+ CD4
T"

groundtruth[groundtruth$RCA == 9 | groundtruth$RCA == 16,]$consensus = "T5"
groundtruth[groundtruth$ADT_pca==1 & groundtruth$consensus == "T5",]$consensus = "CD4 T naive"
```

```
groundtruth[groundtruth$ADT_pca==3 & groundtruth$consensus == "T5",]$consensus = "CD4 T
Memory"
groundtruth[groundtruth$ADT_pca==4 & groundtruth$consensus == "T5",]$consensus = "CD8 T
Memory"
groundtruth[groundtruth$ADT_pca==6 & groundtruth$consensus== "T5",]$consensus = "TIGIT+ CD4 T"
groundtruth[groundtruth$ADT_pca==7 & groundtruth$consensus== "T5",]$consensus = "CD8 T Naive"
groundtruth[groundtruth$ADT_pca==9 & groundtruth$consensus == "T5",]$consensus = "TIGIT+ CD8 T"

groundtruth[groundtruth$RCA == 10,]$consensus = "T6"
groundtruth[groundtruth$ADT_pca==1 & groundtruth$consensus == "T6",]$consensus = "CD4 T naive"
groundtruth[groundtruth$ADT_pca==3 & groundtruth$consensus == "T6",]$consensus = "CD4 T
Memory"
groundtruth[groundtruth$ADT_pca==4 & groundtruth$consensus == "T6",]$consensus = "CD8 T
Memory"
groundtruth[groundtruth$ADT_pca==6 & groundtruth$consensus== "T6",]$consensus = "TIGIT+ CD4 T"
groundtruth[groundtruth$ADT_pca==7 & groundtruth$consensus== "T6",]$consensus = "CD8 T Naive"
groundtruth[groundtruth$ADT_pca==9 & groundtruth$consensus == "T6",]$consensus = "TIGIT+ CD8 T"

groundtruth[groundtruth$RCA == 11,]$consensus = "NC_Mono"
groundtruth[groundtruth$ADT_pca==0 & groundtruth$consensus == "NC_Mono",]$consensus = "CD14
Mono"
groundtruth[groundtruth$ADT_pca==8 & groundtruth$consensus == "NC_Mono",]$consensus = "CD16
Mono"
groundtruth[groundtruth$ADT_pca==10 & groundtruth$consensus == "NC_Mono",]$consensus = "CD14
Mono"

groundtruth[groundtruth$RCA == 12,]$consensus = "mDC"

groundtruth[groundtruth$RCA == 13,]$consensus = "B2"

groundtruth[groundtruth$RCA == 14,]$consensus = "pDC"

groundtruth[groundtruth$RCA == 15,]$consensus = "Plasma"

groundtruth[groundtruth$RCA == 20,]$consensus = "Progenitor"

dim(groundtruth[groundtruth$consensus==0,])

sort(table(groundtruth$consensus))

groundtruth[groundtruth$consensus == "T1" | groundtruth$consensus == "T2" |
        groundtruth$consensus == "T3" | groundtruth$consensus == "T4"|
        groundtruth$consensus == "T5" | groundtruth$consensus == "T6",]$consensus = "T"
groundtruth[groundtruth$consensus == "NC_Mono",]$consensus = "CD16 Mono"

groundtruthDataList = list()
groundtruthDataList[["pbmc"]] = data[,rownames(groundtruth[groundtruth$consensus!=0,])]
groundtruthGroupList = list()
```

```
groundtruthGroupList[["pbmc"]] = groundtruth[groundtruth$consensus!=0,]$consensus

save(groundtruthDataList,groundtruthGroupList,file="groundtruth_PBMC_Monaco.RData")

# Find the consensus groundtruth based on RCA and Seurat clustering
groundtruth = as.data.frame(data_obj$group_labels_color$groupLabel)
rownames(groundtruth) = colnames(data)
colnames(groundtruth) = "RCA"
groundtruth$RNA = data_obj$seurat_labels
groundtruth$ADT_Umap = data_obj$adt_umap_labels
groundtruth$ADT_pca = data_obj$adt_pca_labels

groundtruth$consensus = 0
groundtruth[groundtruth$RCA == 1 | groundtruth$RCA == 17 | groundtruth$RCA == 18,]$consensus =
"C_Mono"
groundtruth[groundtruth$ADT_pca==0 & groundtruth$consensus == "C_Mono",]$consensus = ""
groundtruth[groundtruth$ADT_pca==2 & groundtruth$RCA == 1,]$consensus = "CD4 T Memory"
groundtruth[groundtruth$ADT_pca==4 & groundtruth$RCA == 1,]$consensus = "CD8 T Memory"
groundtruth[groundtruth$ADT_pca==6 & groundtruth$RCA == 1,]$consensus = "CD8 T naive"
groundtruth[groundtruth$ADT_pca==7 & groundtruth$RCA == 1,]$consensus = "TIGIT+ CD8 T"
groundtruth[groundtruth$ADT_pca==9 & groundtruth$RCA == 1,]$consensus = "TIGIT+ CD4 T"

groundtruth[groundtruth$RCA == 1 | groundtruth$RCA == 17 | groundtruth$RCA == 18,]$consensus =
"C_Mono"
groundtruth[groundtruth$ADT_pca==0 & groundtruth$consensus == "C_Mono",]$consensus = "CD14
Mono"
groundtruth[groundtruth$ADT_pca==1 & groundtruth$RCA == 2,]$consensus = "CD16 Mono"
groundtruth[groundtruth$ADT_pca==10 & groundtruth$RCA == 2,]$consensus = "CD14 Mono"
groundtruth[groundtruth$ADT_pca==11 & groundtruth$RCA == 2,]$consensus = "CD16 Mono"

groundtruth[groundtruth$RCA == 3,]$consensus = "NK"

groundtruth[groundtruth$RCA == 4,]$consensus = "B"

groundtruth[groundtruth$RCA == 5,]$consensus = "DC"

groundtruth[groundtruth$RCA == 6,]$consensus = "Plasma"

groundtruth[groundtruth$RCA == 7,]$consensus = "precursors"

dim(groundtruth[groundtruth$consensus==0,])

table(groundtruth$consensus)

groundtruthDataList = list()
groundtruthDataList[["pbmc"]] = data[,rownames(groundtruth[groundtruth$consensus!=0,])]
groundtruthGroupList = list()
groundtruthGroupList[["pbmc"]] = groundtruth[groundtruth$consensus!=0,]$consensus
```

```
save(groundtruthDataList,groundtruthGroupList,file="groundtruth_PBMC.RData")

################################VDJ################################################
# load the original data
data_original = readMM("/mnt/volume1/project/vdj_8k/matrix.mtx.gz")
data_genes =
read.csv("/mnt/volume1/project/vdj_8k/features.tsv.gz",header=F,sep="\t",stringsAsFactors = F)
data_cells =
read.csv("/mnt/volume1/project/vdj_8k/barcodes.tsv.gz",header=F,sep="\t",stringsAsFactors = F)

data_original@Dimnames[[1]] = data_genes$V2
data_original@Dimnames[[2]] = data_cells$V1

vdj.rna.collapsed <- CollapseSpeciesExpressionMatrix(data_original[1:33538,])
vdj <- CreateSeuratObject(vdj.rna.collapsed,min.cells = 100, min.features = 500, project = "vdj")
mito.features <- grep(pattern = "^MT-", x = rownames(x = vdj), value = TRUE)
percent.mito <- Matrix::colSums(x = GetAssayData(object = vdj, slot = 'counts')[mito.features, ]) /
Matrix::colSums(x = GetAssayData(object = vdj, slot = 'counts'))
vdj[['percent.mito']] <- percent.mito
VlnPlot(object = vdj, features = c("nFeature_RNA", "nCount_RNA", "percent.mito"), ncol = 3)
vdj <- subset(x = vdj, subset = nFeature_RNA > 500 & nFeature_RNA < 4000 & percent.mito < 0.3)
vdj <- NormalizeData(vdj)
vdj <- FindVariableFeatures(vdj,selection.method = "vst",nfeatures = 1000)
vdj <- ScaleData(vdj, display.progress = FALSE)
vdj <- RunPCA(vdj,features = VariableFeatures(object = vdj))
ElbowPlot(vdj)
vdj <- FindNeighbors(object = vdj, reduction = "pca", dims = 1:15)
vdj <- FindClusters(vdj, resolution = 0.1)
vdj <- RunTSNE(vdj, dims = 1:15)

vdj.markers <- FindAllMarkers(object = vdj, only.pos = TRUE, min.pct = 0.25, logfc.threshold = 0.25)
vdj.markers %>% group_by(cluster) %>% top_n(n = 5, wt = avg_logFC) %>% print(n=26)
filter(vdj.markers, cluster == "0") %>% top_n(n=20,wt=avg_logFC)

new.cluster.ids <- c("CD14 Mono", "CD4 T", "B","CD8 T", "NK", "Unknown", "FCGR3A Mono","DC","CD8
T","Mk")
names(x = new.cluster.ids) <- levels(x = vdj)
vdj <- RenameIdents(object = vdj, new.cluster.ids)
DimPlot(object = vdj, reduction = 'tsne', label = TRUE, pt.size = 0.5)
table(vdj@active.ident)
vdj[["rna_10clusters"]] <- Idents(object = vdj)

# load the ADT UMI data
vdj.adt <- as.matrix(data_original[33539:33555,])
vdj.adt = vdj.adt[,names(vdj@active.ident)]
vdj.cite <- CreateSeuratObject(counts = vdj.adt)
vdj.cite = NormalizeData(object = vdj.cite, method = "CLR")
```

```
vdj.cite <- ScaleData(vdj.cite, features = NULL, display.progress = FALSE)

###### cluster based on ADT data
# Cluster based on PCA
vdj.cite <- RunPCA(vdj.cite, features = rownames(vdj.cite@assays$RNA))
vdj.cite <- FindNeighbors(object = vdj.cite, reduction = "pca", dims = 1:16)
vdj.cite <- FindClusters(vdj.cite, resolution = 0.2, Reduction.type = "pca")
vdj.cite[["rna_10clusters"]] <- Idents(object = vdj)
adt_pca_labels = vdj.cite@active.ident
vdj.cite <- RunTSNE(vdj.cite, dims = 1:16)
DimPlot(object = vdj.cite, reduction = 'tsne', label = TRUE, pt.size = 0.5)
vdj.small <- SubsetData(vdj.cite, max.cells.per.ident = 300)
adt.markers <- FindAllMarkers(vdj.small, only.pos = TRUE)
DoHeatmap(vdj.small, features = unique(adt.markers$gene),size=3)
DoHeatmap(vdj.small, features = unique(adt.markers$gene),size=3,group.by = "rna_10clusters")

# Cluser based on UMAP
vdj.cite <- RunUMAP(vdj.cite,features = rownames(vdj.cite@assays$RNA))
vdj.cite <- FindNeighbors(object = vdj.cite, reduction = "umap", dims = 1:2)
vdj.cite <- FindClusters(vdj.cite, resolution = 0.01, Reduction.type = "umap")
DimPlot(object = vdj.cite, reduction = 'umap', label = TRUE, pt.size = 0.5)
vdj.small <- SubsetData(vdj.cite, max.cells.per.ident = 300)
adt.markers <- FindAllMarkers(vdj.small, only.pos = TRUE)
DoHeatmap(vdj.small, features = unique(adt.markers$gene),size=3)

# load data after filtering
data=benchmarkDataList$VDJ # in normal domain after filtering
colnames(data) <- paste0(colnames(data),"-1")

# Intersection of data and the original data
data = data[, colnames(data) %in% colnames(vdj@assays$RNA@data)]

# Run RCA global panel to label cells
data_obj = dataConstruct(as.matrix(data))
data_obj = geneFilt(obj_in = data_obj)
data_obj = cellNormalize(data_obj)
data_obj = dataTransform(data_obj)
#data_obj = featureConstruct(data_obj,method = "GlobalPanel",power = 4)
source('/mnt/volume1/project/code/featureConstructMonaco.R')
data_obj = featureConstructMonaco(data_obj,method = "MonacoPanel",power = 4)

data_obj = cellClust(data_obj,deepSplit_wgcna=2)
RCAPlot(data_obj)

#get the seurat label for filtered data
data_obj$seurat_labels = vdj@active.ident[colnames(data)]
data_obj$adt_umap_labels = vdj.cite@active.ident[colnames(data)]
data_obj$adt_pca_labels = adt_pca_labels[colnames(data)]
```

```
#Umap using RCA global panel clustering
seed = 42
seurat_obj = CreateSeuratObject(data_obj$fpkm_for_clust)
seurat_obj <- ScaleData(object = seurat_obj, display.progress = FALSE)

seurat_obj = RunUMAP(obj=seurat_obj,features = rownames(seurat_obj@assays$RNA@data))
seurat_obj@active.ident = as.factor(data_obj$group_labels_color$groupLabel)
names(seurat_obj@active.ident) = colnames(data)

colors = as.character(unique(data_obj$group_labels_color$dynamicColors))
labels = unique(data_obj$group_labels_color$groupLabel)
colors_sorted = colors[sort.int(labels,index.return = T)$ix]
DimPlot(object = seurat_obj, reduction = 'umap',group.by = "ident",cols = colors_sorted)

# label using seurat labels
seurat_obj@active.ident = as.factor(data_obj$seurat_labels)
names(seurat_obj@active.ident) = colnames(data)

colors = unique(labels2colors(data_obj$seurat_labels))
DimPlot(object = seurat_obj, reduction = 'umap',group.by = "ident",cols = colors)

# label using ADT labels
seurat_obj@active.ident = as.factor(data_obj$adt_umap_labels)
names(seurat_obj@active.ident) = colnames(data)

colors = unique(labels2colors(data_obj$adt_umap_labels))
DimPlot(object = seurat_obj, reduction = 'umap',group.by = "ident",cols = colors)

source('/mnt/volume1/project/plotHeatmap.R')
cellTypeDEPlot(data_obj$fpkm_for_clust,cellTree = data_obj$cellTree,clusterLabels =
data_obj$group_labels_color$groupLabel,dynamicColors = data_obj$dynamicColors,filename =
"heatmap_RCA_Monacopanel_deepsplit2")

# Find the consensus groundtruth based on RCA and Seurat clustering
groundtruth = as.data.frame(data_obj$group_labels_color$groupLabel)
rownames(groundtruth) = colnames(data)
colnames(groundtruth) = "RCA"
groundtruth$RNA = data_obj$seurat_labels
groundtruth$ADT_Umap = data_obj$adt_umap_labels
groundtruth$ADT_pca = data_obj$adt_pca_labels

groundtruth$consensus = 0
groundtruth[groundtruth$RCA == 1,]$consensus = "Mono"

groundtruth[groundtruth$RCA == 2,]$consensus = "T"
groundtruth[groundtruth$ADT_pca==1 & groundtruth$RCA == 2,]$consensus = "CD4 T"
groundtruth[groundtruth$ADT_pca==3 & groundtruth$RCA == 2,]$consensus = "CD8 T"
```

```
groundtruth[groundtruth$RCA == 3,]$consensus = "B"

groundtruth[groundtruth$RCA == 4,]$consensus = "MAIT"

groundtruth[groundtruth$ADT_pca==5 & groundtruth$RCA == 5,]$consensus = "NK"

groundtruth[groundtruth$ADT_pca!=5 & groundtruth$RCA == 5,]$consensus = "CD8 TE"

groundtruth[groundtruth$RCA == 6,]$consensus = "mDC"

groundtruth[groundtruth$RCA == 7,]$consensus = "pDC"

groundtruth[groundtruth$RCA == 8,]$consensus = "Mono"

groundtruth[groundtruth$RCA == 9,]$consensus = "plasma"

groundtruth[groundtruth$RCA == 10,]$consensus = "basophil"

groundtruth[groundtruth$RCA == 11,]$consensus = "B"

groundtruth[groundtruth$RCA == 12,]$consensus = "neutrophil"

groundtruth[groundtruth$RCA == 15,]$consensus = "progenitor"

dim(groundtruth[groundtruth$consensus==0,])

table(groundtruth$consensus)

groundtruthDataList = list()
groundtruthDataList[["vdj"]] = data[,rownames(groundtruth[groundtruth$consensus!=0,])]
groundtruthGroupList = list()
groundtruthGroupList[["vdj"]] = groundtruth[groundtruth$consensus!=0,]$consensus

save(groundtruthDataList,groundtruthGroupList,file="groundtruth_vdj.RData")
```

Supplementary Note 4 – Preprocessing and scConsensus Execution for FACS-sorted PBMC data

FACS sorted PBMC data from Zheng et al. (2017)

```
# 26k FACS_sorted PBMC object preparation

pipestance_path1 = "~/cd14_monocytes/outs/filtered_gene_bc_matrices_mex/hg19/"
pipestance_path2 = "~/CD19+ B Cells/outs/filtered_gene_bc_matrices_mex/hg19/"
pipestance_path3 = "~/CD34+ Cells/outs/filtered_gene_bc_matrices_mex/hg19/"
pipestance_path4 = "~/CD4+ Helper T Cells/outs/filtered_gene_bc_matrices_mex/hg19/"
pipestance_path5 = "~/CD4+CD25+ Regulatory T Cells/outs/filtered_gene_bc_matrices_mex/hg19/"
pipestance_path6 = "~/CD4+CD45RA+CD25- Naive T cells/outs/filtered_gene_bc_matrices_mex/hg19/"
pipestance_path7 = "~/CD4+CD45RO+ Memory T Cells/outs/filtered_gene_bc_matrices_mex/hg19/"
pipestance_path8 = "~/CD56+ Natural Killer Cells/outs/filtered_gene_bc_matrices_mex/hg19/"
pipestance_path9 = "~/CD8+ Cytotoxic T cells/outs/filtered_gene_bc_matrices_mex/hg19/"
pipestance_path10 = "~/CD8+CD45RA+ Naive Cytotoxic T
Cells/outs/filtered_gene_bc_matrices_mex/hg19/"

set.seed(1)
pbmc1.data <- Read10X(data.dir = pipestance_path1)
pbmc1.data <- pbmc1.data[,sample(1:ncol (pbmc1.data), 2600)]
pbmc1 <- CreateSeuratObject(counts =  pbmc1.data, project = "CD14_Monocytes")
pbmc2.data <- Read10X(data.dir = pipestance_path2)
pbmc2.data <- pbmc2.data[,sample(1:ncol (pbmc2.data), 2600)]
pbmc2 <- CreateSeuratObject(counts = pbmc2.data, project = "B_Cells")
pbmc3.data <- Read10X(data.dir = pipestance_path3)
pbmc3.data <- pbmc3.data[,sample(1:ncol (pbmc3.data), 2600)]
pbmc3 <- CreateSeuratObject(counts = pbmc3.data, project = "CD34_Cells")
pbmc4.data <- Read10X(data.dir = pipestance_path4)
pbmc4.data <- pbmc4.data[,sample(1:ncol (pbmc4.data), 2600)]
pbmc4 <- CreateSeuratObject(counts = pbmc4.data, project = "CD4_T-Helper")
pbmc5.data <- Read10X(data.dir = pipestance_path5)
pbmc5.data <- pbmc5.data[,sample(1:ncol (pbmc5.data), 2600)]
pbmc5 <- CreateSeuratObject(counts = pbmc5.data, project = "T_Reg")
pbmc6.data <- Read10X(data.dir = pipestance_path6)
pbmc6.data <- pbmc6.data[,sample(1:ncol (pbmc6.data), 2600)]
pbmc6 <- CreateSeuratObject(counts = pbmc6.data, project = "CD4_T-Naive")
pbmc7.data <- Read10X(data.dir = pipestance_path7)
pbmc7.data <- pbmc7.data[,sample(1:ncol (pbmc7.data), 2600)]
pbmc7 <- CreateSeuratObject(counts = pbmc7.data, project = "CD4_T-Memory")
pbmc8.data <- Read10X(data.dir = pipestance_path8)
pbmc8.data <- pbmc8.data[,sample(1:ncol (pbmc8.data), 2600)]
pbmc8 <- CreateSeuratObject(counts = pbmc8.data, project = "NK_Cells")
pbmc9.data <- Read10X(data.dir = pipestance_path9)
pbmc9.data <- pbmc9.data[,sample(1:ncol (pbmc9.data), 2600)]
pbmc9 <- CreateSeuratObject(counts = pbmc9.data, project = "CD8_T-Cytotoxic")
```

```
pbmc10.data <- Read10X(data.dir = pipestance_path10)
pbmc10.data <- pbmc10.data[,sample(1:ncol (pbmc10.data), 2600)]
pbmc10 <- CreateSeuratObject(counts = pbmc10.data, project = "CD8_T-Naive")
pbmc_26k_S.v3 <- merge(pbmc1, y = c(pbmc2, pbmc3, pbmc4, pbmc5, pbmc6, pbmc7, pbmc8, pbmc9,
pbmc10), add.cell.ids = c("1", "2", "3", "4", "5", "6", "7", "8", "9", "10"), project = "PBMC26K")
# QC filtering
pbmc_26k_S.v3[["percent.mt"]] <- PercentageFeatureSet(pbmc_26k_S.v3, pattern = "^MT-")
VlnPlot(pbmc_26k_S.v3, features = c("nFeature_RNA", "nCount_RNA", "percent.mt"), ncol = 3, pt.size =
0)
plot1 <- FeatureScatter(pbmc_26k_S.v3, feature1 = "nCount_RNA", feature2 = "percent.mt")
plot2 <- FeatureScatter(pbmc_26k_S.v3, feature1 = "nCount_RNA", feature2 = "nFeature_RNA")
CombinePlots(plots = list(plot1, plot2))
pbmc_26k_S.v3_Filtered <- subset(pbmc_26k_S.v3, subset = nFeature_RNA > 300 & nFeature_RNA <
2000 & percent.mt < 8)
# Unsupervised clustering (seurat pipeline)
pbmc_26k_S.v3_Filtered <- NormalizeData(pbmc_26k_S.v3_Filtered, normalization.method =
"LogNormalize", scale.factor = 10000)
pbmc_26k_S.v3_Filtered <- FindVariableFeatures(pbmc_26k_S.v3_Filtered, selection.method = "vst",
nfeatures = 2000)
top10 <- head(VariableFeatures(pbmc_26k_S.v3_Filtered), 10)
plot1 <- VariableFeaturePlot(pbmc_26k_S.v3_Filtered)
plot2 <- LabelPoints(plot = plot1, points = top10, repel = TRUE)
CombinePlots(plots = list(plot1, plot2))
pbmc_26k_S.v3_Filtered <- ScaleData(pbmc_26k_S.v3_Filtered, vars.to.regress = "percent.mt")
pbmc_26k_S.v3_Filtered <- RunPCA(pbmc_26k_S.v3_Filtered, features = VariableFeatures(object =
pbmc_26k_S.v3_Filtered))
DimHeatmap(pbmc_26k_S.v3_Filtered, dims = 1:15, cells = 500, balanced = TRUE)
pbmc_26k_S.v3_Filtered <- JackStraw(pbmc_26k_S.v3_Filtered, num.replicate = 100)
pbmc_26k_S.v3_Filtered <- ScoreJackStraw(pbmc_26k_S.v3_Filtered, dims = 1:20)
JackStrawPlot(pbmc_26k_S.v3_Filtered, dims = 1:20)
ElbowPlot(pbmc_26k_S.v3_Filtered)
pbmc_26k_S.v3_Filtered <- FindNeighbors(pbmc_26k_S.v3_Filtered, reduction = "pca", dims = 1:20)
pbmc_26k_S.v3_Filtered <- FindClusters(pbmc_26k_S.v3_Filtered, resolution = 0.2, algorithm = 1)
pbmc_26k_S.v3_Filtered <- RunUMAP(pbmc_26k_S.v3_Filtered, dims = 1:20)
DimPlot(pbmc_26k_S.v3_Filtered, reduction = "umap", group.by = "nFeature_RNA")
DimPlot(pbmc_26k_S.v3_Filtered, reduction = "umap", group.by = "orig.ident")
FeaturePlot(pbmc_26k_S.v3_Filtered, features = c("IL7R", "CD8A", "GNLY", "CD79A","CD14","FCER1A",
"FCGR3A", "SERPINF1", "PPBP"))
pbmc.markers <- FindAllMarkers(pbmc_26k_S.v3_Filtered, only.pos = TRUE, min.pct = 0.25,
logfc.threshold = 0.25)

# Save the seurat object:
saveRDS(pbmc_26k_S.v3_Filtered, file = "pbmc_26k_S.v3_Filtered.rds")
```
